# Elucidating the physiological significance of iodate reduction in *Shewanella* species

**DOI:** 10.1002/mlf2.70096

**Published:** 2026-08-23

**Authors:** Ziyi Wang, Lingyu Hou, Yidan Hu, Zhou Jiang, Yiran Dong, Liang Shi, Yongguang Jiang

**Affiliations:** ^1^ Department of Biological Sciences and Technology School of Environmental Studies, China University of Geosciences Wuhan China; ^2^ State Key Laboratory of Geomicrobiology and Environment, China University of Geosciences Wuhan China; ^3^ Hubei Key Laboratory of Wetland Evolution & Eco‐Restoration Wuhan China

**Keywords:** detoxification, extracellular respiration, iodate reduction, oxidative stress, *Shewanella*

## Abstract

The reduction of iodate by *Shewanella* species plays a crucial role in iodine biogeochemical cycling. However, the physiological significance of this reduction process remains poorly understood, because *Shewanella* species are capable of reducing only low concentrations of iodate. This study systematically examined the physiological and transcriptomic responses of *Shewanella oneidensis* MR‐1 to iodate exposure. Our results showed that phenol‐mediated elimination of hypoiodous acid did not increase the upper limit of iodate reduction achieved by *S. oneidensis* MR‐1, and dimethyl sulfoxide was preferentially utilized over iodate by the organism. When iodate served as the sole electron acceptor, no increase in total cellular protein was observed in *S. oneidensis* MR‐1, in contrast to marine *Shewanella* strains, which exhibited increased production of total cellular protein at 0.25 and 0.50 mM iodate. Furthermore, anaerobic growth of *S. oneidensis* MR‐1 and the Δ*dmsEFAB* strain was dose‐dependently inhibited by iodate, which induced significant oxidative stress. Transcriptomic analysis revealed that the mRNA levels of *mtrCAB* and several genes encoding antioxidant enzymes were elevated during iodate reduction. Deletion mutants of *so1563* (glutathione peroxidase), *so1659* (*c*‐type cytochrome), and *so0534* (arsenate permease) exhibited reduced capacities for iodate reduction or H_2_O_2_ degradation. Notably, supplementation of H_2_O_2_ in the medium compromised iodate reduction in both wild‐type and mutant strains. Collectively, these findings provide evidence that iodate reduction in *Shewanella* primarily functions as a detoxification mechanism rather than a process for energy conservation. The results further highlight distinct adaptive strategies employed by *Shewanella* species in freshwater and marine environments with different iodine concentrations.

## Impact statement

*Shewanella* are widespread bacteria that contribute to the recycling of important elements in nature. For example, *Shewanella* can transform iodate into iodide in the ocean. This iodide is either taken up by seaweed or reacts with ozone at the seawater surface to form gaseous iodine. Previous study revealed that *Shewanella* only reduce iodate below 1.0 mM. However, the reason why *Shewanella* perform iodate reduction only at low concentrations has not been fully understood. Our study shows that *Shewanella* reduces iodate as a protective response against oxidative damage. Several key proteins, including peroxidases, *c*‐type cytochromes, and an arsenate transporter, were found to contribute to the neutralization of oxidative stress during iodate reduction. Additionally, marine *Shewanella* were shown to utilize iodate as an electron acceptor to sustain protein production, indicating their adaptation to iodine‐rich environments.

## INTRODUCTION

Microbially mediated iodate (${\text{IO}}_{3}^{-}$) reduction to iodide ($I^{-}$) constitutes a critical biogeochemical process in global iodine cycling^1^, ^2^. Both anaerobic and aerobic ${\text{IO}}_{3}^{-}$ reduction by marine bacteria and phytoplankton drive $I^{-}$ enrichment in surface seawater, where subsequent reactions with ozone facilitate the emission of iodine to the atmosphere^3^, ^4^, ^5^, ^6^, ^7^. Bacterial processes, including ${\text{IO}}_{3}^{-}$ reduction, sulfate reduction, iron reduction, and dehalogenation of organic iodine, may also contribute to $I^{-}$ accumulation in deep groundwater systems^1^, ^8^, ^9^, ^10^, ^11^, ^12^.

Dissimilatory iodate‐reducing microorganisms (DIRMs) utilize ${\text{IO}}_{3}^{-}$ as a terminal electron acceptor, and axenic cultures have been isolated from diverse aquatic environments, including seawater, groundwater, and freshwater systems^6^, ^10^, ^13^. The favorable redox potential of the ${\text{IO}}_{3}^{-}$/$I^{-}$ couple (*E°′* = +0.720 V), which is intermediate between $O_{2}$/$H_{2}O$ (+0.820 V) and ${\text{NO}}_{3}^{-}$/$N_{2}$ (+0.713 V)^13^, ^14^, enables energy conservation through ${\text{IO}}_{3}^{-}$ respiration. This metabolic capability has been experimentally confirmed in multiple DIRM strains (e.g., *Pseudomonas* sp. SCT, *Denitromonas* sp. IR‐12, and *Azoarcus* sp. DN11) grown with acetate as the electron donor, demonstrating the respiratory utilization of 1−8 mM ${\text{IO}}_{3}^{-}$ as the sole electron acceptor^10^, ^13^, ^14^, ^15^, ^16^. *Shewanella* are ubiquitously distributed bacteria in anoxic environments, and several species in this genus exhibit extracellular ${\text{IO}}_{3}^{-}$ reduction capacity^17^, ^18^, ^19^, ^20^. However, in contrast to DIRMs, *Shewanella* spp. are capable of reducing ${\text{IO}}_{3}^{-}$ only at low concentrations^7^, ^21^. For example, the freshwater strain *S. oneidensis* MR‐1 shows a limited ${\text{IO}}_{3}^{-}$ reduction capacity, with reduction observed only below 1.0 mM^21^, ^22^. Moreover, *Shewanella putrefaciens* MR‐4 from the Arabian Sea exhibits limited growth stimulation at low ${\text{IO}}_{3}^{-}$ concentrations^7^. Therefore, compared to DIRMs, the physiological role of ${\text{IO}}_{3}^{-}$ reduction in *Shewanella* spp. remains uncertain.

In DIRMs, ${\text{IO}}_{3}^{-}$ is reduced by an iodate‐reductase complex localized in the periplasm^14^, ^16^. The complex comprises a molybdopterin‐containing enzyme (IdrA) belonging to the dimethyl sulfoxide (DMSO) reductase family, an iron‐sulfur protein (IdrB), and two di‐heme cytochromes (IdrP1 and IdrP2) homologous to cytochrome *c* peroxidase. Current models suggest that IdrAB‐mediated ${\text{IO}}_{3}^{-}$ reduction yields hypoiodous acid (HIO) and hydrogen peroxide (H_2_O_2_), with electrons derived from the quinone pool^16^. HIO undergoes either enzymatic disproportionation to $I^{-}$/O_2_ or spontaneous conversion to $I^{-}$/${\text{IO}}_{3}^{-}$, whereas IdrP1P2 likely degrades H_2_O_2_ ^14^, ^16^.

Although the genome of *S. oneidensis* MR‐1 contains no *idr* genes, it has a metal‐reducing pathway formed by the MtrCAB complex and a DMSO‐reducing conduit composed of the DmsEFAB complex^23^, ^24^, ^25^. DmsA and DmsB are homologous, respectively, to the molybdopterin‐containing and the iron‐sulfur‐cluster‐containing subunits of the DMSO‐reductase complex in *Escherichia coli* ^24^. Our previous study revealed that DmsEFAB mediates ${\text{IO}}_{3}^{-}$ reduction in *S. oneidensis* MR‐1, with MtrCAB facilitating H_2_O_2_ detoxification^20^. Supporting this model, *mtrCAB* deletion strains exhibit H_2_O_2_ accumulation and impaired ${\text{IO}}_{3}^{-}$ reduction^20^. In addition, previous studies demonstrated that DmsA is essential for optimal reduction of ${\text{IO}}_{3}^{-}$ and its analog bromate in *S. oneidensis* MR‐1^26^, ^27^. Our study confirmed that HIO is an intermediate of ${\text{IO}}_{3}^{-}$ reduction by trapping HIO with phenol and detecting the iodophenols produced^20^. Furthermore, phenol‐mediated elimination of HIO was demonstrated to significantly enhance the reduction kinetics of ${\text{IO}}_{3}^{-}$ ^20^. Given the oxidative toxicity of H_2_O_2_ and HIO, these findings suggest that these compounds may likewise constrain the ${\text{IO}}_{3}^{-}$ reduction capacity of *Shewanella*. However, the oxidative stress produced during ${\text{IO}}_{3}^{-}$ reduction and the responses of *Shewanella* cells were overlooked in the previous studies.

The ocean typically exhibits a total iodine concentration ranging from 0.4 to 0.5 μM, which can be utilized by both DIRMs and *Shewanella* ^28^. Consequently, in addition to DIRMs that possess a significant capacity for ${\text{IO}}_{3}^{-}$ reduction, *Shewanella* species also play a crucial role in the cycling of iodine. In this study, the physiological and transcriptomic responses of *S. oneidensis* MR‐1 to ${\text{IO}}_{3}^{-}$ were systematically investigated, with the aim of elucidating the adaptive mechanisms employed by this bacterium during ${\text{IO}}_{3}^{-}$ reduction. To assess the functional roles of several genes that exhibit upregulated transcription during ${\text{IO}}_{3}^{-}$ reduction, mutants lacking these specific genes were constructed and characterized. Additionally, two marine *Shewanella* strains, including *Shewanella livingstonensis* CCTCC AB2020017 and *Shewanella piezotolerans* MCCC 1A01966, were evaluated for their growth utilizing ${\text{IO}}_{3}^{-}$ as the sole electron acceptor.

## RESULTS

### Limited ${\mathrm{IO}}_{3}^{-}$ reduction by *S. oneidensis* MR‐1

**Figure 1:**
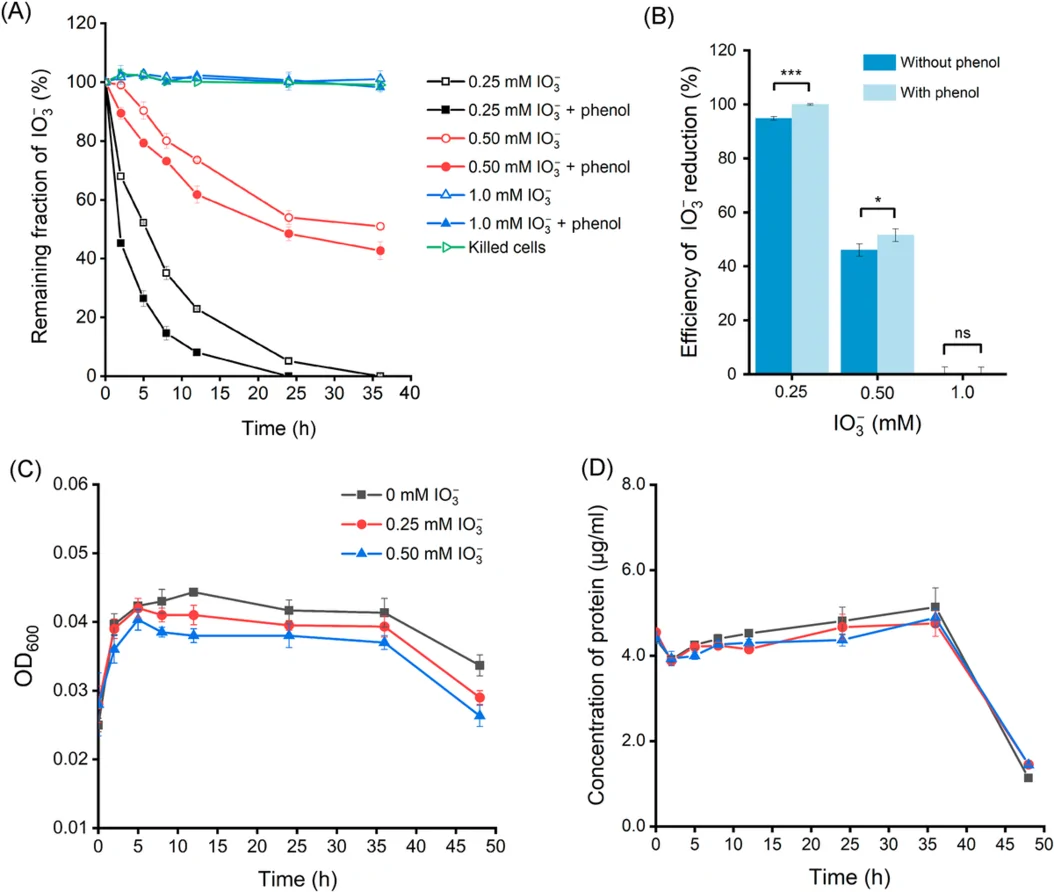
Characterization of *Shewanella oneidensis* MR‐1 with ${\text{IO}}_{3}^{-}$ as the terminal electron acceptor. (A) ${\text{IO}}_{3}^{-}$ reduction traces at 0.25, 0.50, and 1.0 mM of initial ${\text{IO}}_{3}^{-}$ with or without phenol. No reduction was observed in the killed cells. (B) Efficiency of ${\text{IO}}_{3}^{-}$ reduction at 24 h with or without phenol. (C) Optical density of bacterial cultures at 600 nm during ${\text{IO}}_{3}^{-}$ reduction. (D) Concentration of total cellular protein during ${\text{IO}}_{3}^{-}$ reduction. Bacterial cells were inoculated with a starting OD_600_ of 0.02 in (C, D). For points with no error bar, the error is smaller than the size of the symbol. Statistical method: Student's *t*‐test. ns, *p* > 0.05; 0.01 < *p < 0.05; ***p < 0.001. The molar ratio between phenol and ${\text{IO}}_{3}^{-}$ is 4:1.

To explore the effect of phenol on the ${\text{IO}}_{3}^{-}$ reduction by *S. oneidensis* MR‐1, reduction experiments were conducted with or without phenol. *S. oneidensis* MR‐1 exhibited limited ${\text{IO}}_{3}^{-}$ reduction capacity, with effective reduction only observed at concentrations below 1.0 mM, regardless of phenol supplementation (Figure 1A). Quantitative analysis revealed that, under phenol‐free conditions, the strain achieved reduction efficiencies of 94.8% and 46.0% for 0.25 and 0.50 mM ${\text{IO}}_{3}^{-}$, respectively, after 24 h of incubation (Figure 1B). Notably, phenol supplementation significantly elevated the initial 5‐h reduction rate from 23.5 to 36.5 μM h^−1^ for 0.25 mM ${\text{IO}}_{3}^{-}$ (*p* < 0.001) and from 9.2 to 19.9 μM h^−1^ for 0.50 mM ${\text{IO}}_{3}^{-}$ (*p* < 0.01). In the presence of phenol, the 24‐h reduction efficiencies for 0.25 and 0.50 mM ${\text{IO}}_{3}^{-}$ were increased by 5.2% (*p* < 0.001) and 5.5% (*p* < 0.05), respectively. Interestingly, the ${\text{IO}}_{3}^{-}$ reduction yields at 24 h were comparable between 0.25 and 0.50 mM. Subsequent experiments involving replenishment of ${\text{IO}}_{3}^{-}$ to its initial concentration (0.25 mM) demonstrated a marked decline in reduction rate compared to the initial supplementation phase (Figure S1).

Regarding growth dynamics of *S. oneidensis* MR‐1, all the cultures exhibited minor increases in optical density (OD_600_) during the initial phase, followed by a stationary phase and subsequent decline after 36 h post ${\text{IO}}_{3}^{-}$ reduction (Figure 1C). This growth pattern correlated with minimal increases in total protein concentration, which similarly decreased after 36 h (Figure 1D). Notably, compared to electron acceptor‐free cultures, the growth of *S. oneidensis* MR‐1 was not enhanced when ${\text{IO}}_{3}^{-}$ served as the sole electron acceptor. In contrast, cultures utilizing fumarate as the sole electron acceptor demonstrated significant improvements in both OD_600_ values and total protein concentrations (Figure S2).

### Preferential utilization of DMSO over ${\mathrm{IO}}_{3}^{-}$ by *S. oneidensis* MR‐1

**Figure 2:**
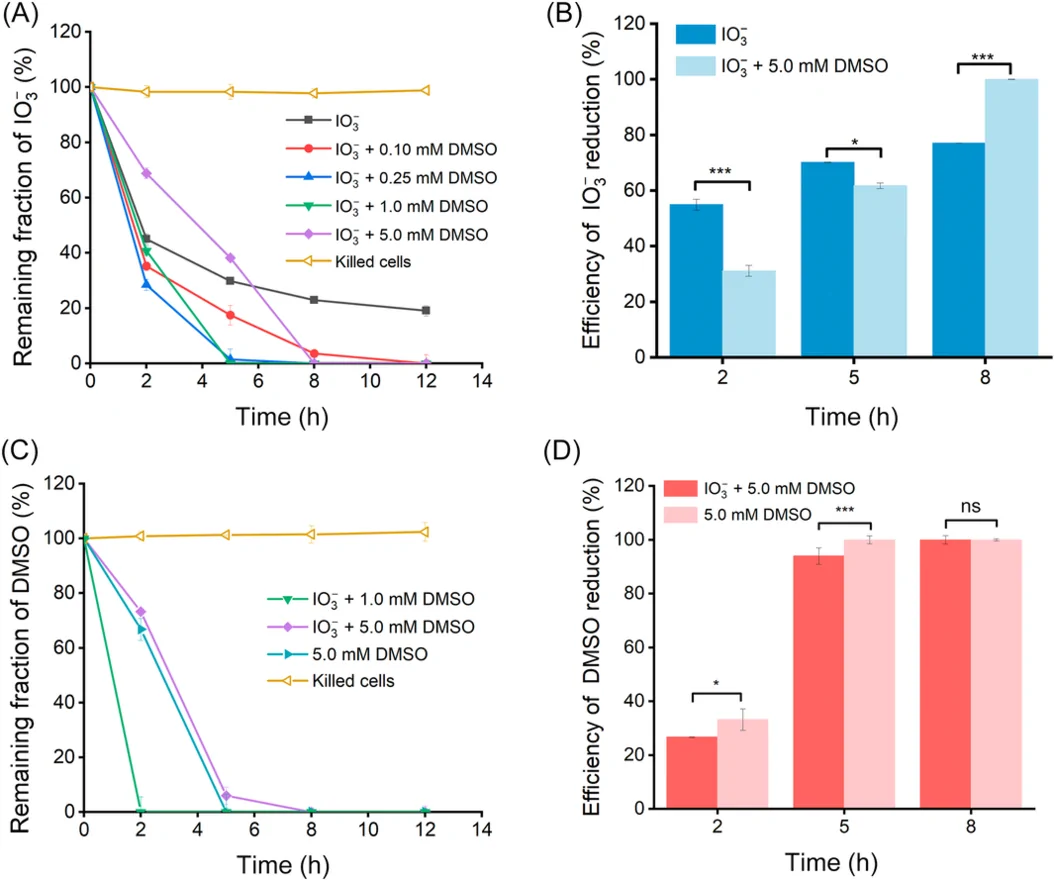
Reduction of ${\text{IO}}_{3}^{-}$ and DMSO by *S. oneidensis* MR‐1. (A, B) ${\text{IO}}_{3}^{-}$ reduction traces (A) and efficiency (B) in the presence of 0, 0.10, 0.25, 1.0, and 5.0 mM of DMSO. (C, D) DMSO reduction traces (C) and efficiency (D) with or without 0.25 mM ${\text{IO}}_{3}^{-}$. One hundred percent of ${\text{IO}}_{3}^{-}$ was equal to 0.25 mM. No reduction was observed in the killed cells. Statistical method: Student's *t*‐test. ns, *p* > 0.05; 0.01 < *p < 0.05; ***p < 0.001. DMSO, dimethyl sulfoxide.

The competitive utilization of DMSO and ${\text{IO}}_{3}^{-}$ by *S. oneidensis* MR‐1 was systematically examined. Experimental data demonstrated that supplementation with 0.10, 0.25, and 1.0 mM DMSO significantly enhanced the reduction kinetics of 0.25 mM ${\text{IO}}_{3}^{-}$ (Figure 2A). Notably, in cultures containing 0.25 mM ${\text{IO}}_{3}^{-}$ and 5.0 mM DMSO, while an initial 2‐h lag phase in ${\text{IO}}_{3}^{-}$ reduction was observed, complete reduction occurred more rapidly than in the DMSO‐free control (Figure 2A,B). Conversely, the presence of ${\text{IO}}_{3}^{-}$ exerted inhibitory effects on DMSO reduction (Figure 2C,D). In systems containing 1.0 mM DMSO and 0.25 mM ${\text{IO}}_{3}^{-}$, complete DMSO reduction was achieved within 2 h (Figure 2C), whereas only 60% of ${\text{IO}}_{3}^{-}$ was reduced during the same period (Figure 2A). These findings collectively suggest that *S. oneidensis* MR‐1 exhibits preferential utilization of DMSO over ${\text{IO}}_{3}^{-}$, and the metabolic energy derived from DMSO reduction appears to facilitate subsequent ${\text{IO}}_{3}^{-}$ reduction.

### ${\mathrm{IO}}_{3}^{-}$ reduction by *S. livingstonensis* and *S. piezotolerans*

**Figure 3:**
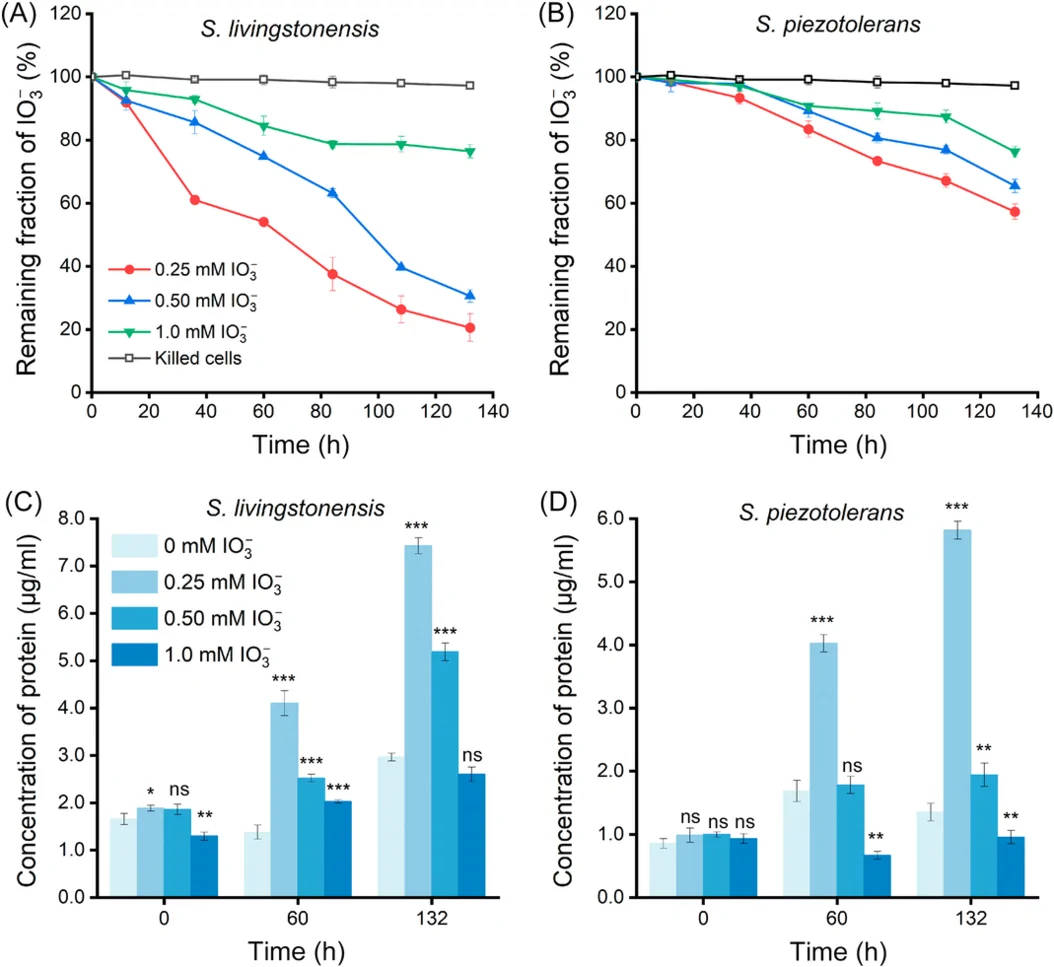
Characterization of *Shewanella livingstonensis* and *Shewanella piezotolerans* with ${\text{IO}}_{3}^{-}$ as the terminal electron acceptor. (A, B) ${\text{IO}}_{3}^{-}$ reduction traces of *S. livingstonensis* (A) and *S. piezotolerans* (B) at 0.25, 0.50, and 1.0 mM of initial ${\text{IO}}_{3}^{-}$. No reduction was observed in the killed cells. (C, D) Concentration of total cellular protein in the cultures of *S. livingstonensis* (C) and *S. piezotolerans* (D) during ${\text{IO}}_{3}^{-}$ reduction. Bacterial cells were inoculated with a starting OD_600_ of 0.02. Statistical method: Student's *t*‐test. ns, *p* > 0.05; 0.01 < *p < 0.05; 0.001 < **p < 0.01; ***p < 0.001.

The ${\text{IO}}_{3}^{-}$ reduction capabilities of two marine *Shewanella* strains were investigated. Details of strains applied in the study are shown in Table S1. In contrast to *S. oneidensis* MR‐1, both *S. livingstonensis* CCTCC AB2020017 and *S. piezotolerans* MCCC 1A01966 demonstrated significant ${\text{IO}}_{3}^{-}$ reduction activity across all tested concentrations (0.25, 0.50, and 1.0 mM) (Figure 3A,B). Quantitative analysis revealed an inverse relationship between ${\text{IO}}_{3}^{-}$ concentration and reduction efficiency. Following 132 h of incubation, *S. livingstonensis* achieved 23.6%−79.4% ${\text{IO}}_{3}^{-}$ reduction, while *S. piezotolerans* exhibited 23.7%−42.7% reduction efficiency across the tested concentration range.

Notably, compared to ${\text{IO}}_{3}^{-}$‐free controls and treatments with 1.0 mM ${\text{IO}}_{3}^{-}$, cultures containing 0.25 and 0.50 mM ${\text{IO}}_{3}^{-}$ displayed significantly increased total protein levels after 132 h (Figure 3C,D). Specifically, *S. livingstonensis* produced 7.63 ± 0.37 μg/ml and 5.19 ± 0.19 μg/ml cellular protein at 0.25 mM and 0.50 mM ${\text{IO}}_{3}^{-}$, respectively, representing 2.7‐fold and 1.9‐fold that of the ${\text{IO}}_{3}^{-}$‐free control (2.78 ± 0.33 μg/ml) after 132 h (Figure 3C). Parallel observations were made for *S. piezotolerans*, with protein yields of 5.82 ± 0.14 μg/ml (0.25 mM ${\text{IO}}_{3}^{-}$) and 1.94 ± 0.18 μg/ml (0.50 mM ${\text{IO}}_{3}^{-}$), corresponding to 4.3‐fold and 1.4‐fold that of the control (1.35 ± 0.14 μg/ml) (Figure 3D). In addition, 1.0 mM ${\text{IO}}_{3}^{-}$ transiently upregulated total protein levels in *S. livingstonensis* at 60 h (Figure 3C), in contrast to its significant inhibitory effect on protein yields in *S. piezotolerans* (Figure 3D).

### Effects of ${\mathrm{IO}}_{3}^{-}$ on the growth of *S. oneidensis* MR‐1 with O_2_ and fumarate

**Figure 4:**
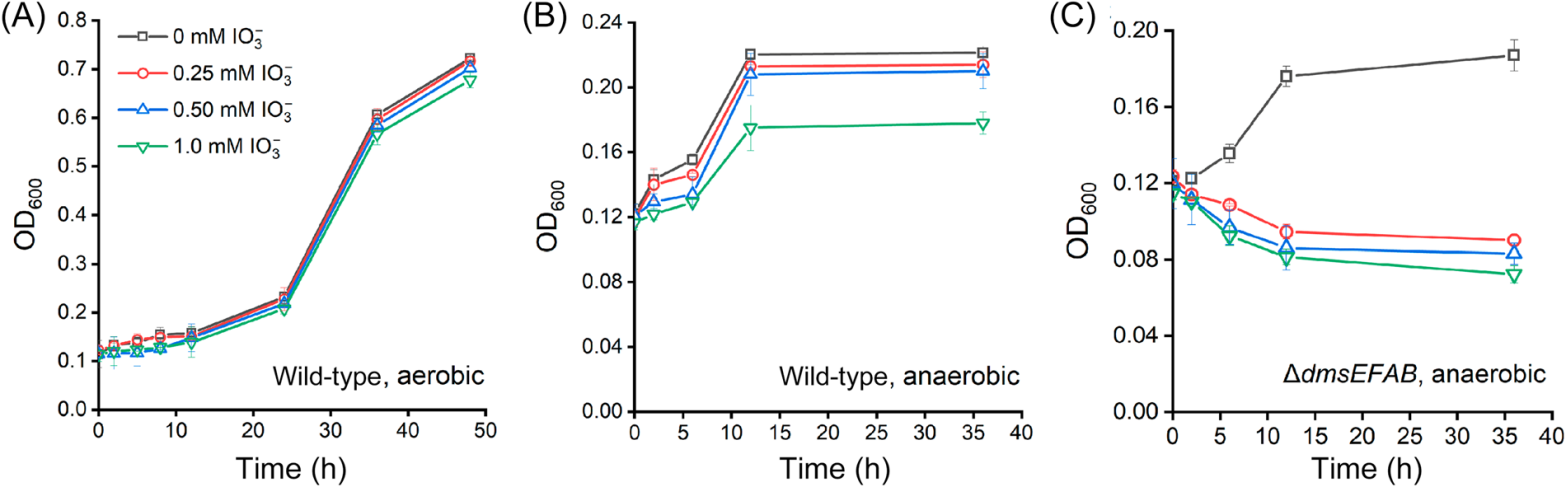
Aerobic and anaerobic growth of *S. oneidensis* MR‐1 cells exposed to different concentrations of ${\text{IO}}_{3}^{-}$. (A) Wild‐type strain grown under aerobic conditions in the presence of 0, 0.25, 0.50, and 1.0 mM of ${\text{IO}}_{3}^{-}$. (B, C) Wild‐type (B) and Δ*dmsEFAB* (C) strains grown under anaerobic conditions in the presence of 0, 0.25, 0.50, and 1.0 mM of ${\text{IO}}_{3}^{-}$. Fumarate was supplemented as the electron acceptor under anaerobic conditions.

Under aerobic conditions, *S. oneidensis* MR‐1 exhibited continuous growth over a 48‐h incubation period regardless of whether ${\text{IO}}_{3}^{-}$ was present (Figure 4A). In contrast, under anaerobic conditions with fumarate as the terminal electron acceptor, the growth of *S. oneidensis* MR‐1 was inhibited by ${\text{IO}}_{3}^{-}$ in a concentration‐dependent manner, with 1.0 mM ${\text{IO}}_{3}^{-}$ resulting in both reduced growth rate and lower final OD_600_ (Figure 4B). Moreover, the Δ*dmsEFAB* strain, which is deficient in ${\text{IO}}_{3}^{-}$ reduction activity, exhibited significant growth inhibition when exposed to ${\text{IO}}_{3}^{-}$ under anaerobic conditions (Figure 4C).

### Effects of ${\mathrm{IO}}_{3}^{-}$ on the intracellular contents of MDA and FSGs

Malondialdehyde (MDA), a terminal product of lipid peroxidation, serves as a biomarker for oxidative damage, while free sulfhydryl groups (FSGs) play a crucial role in scavenging reactive oxygen species (ROS) by cellular antioxidant systems^29^, ^30^. The levels of MDA and FSGs exhibit positive and negative correlations, respectively, with intracellular oxidative stress levels.

**Figure 5:**
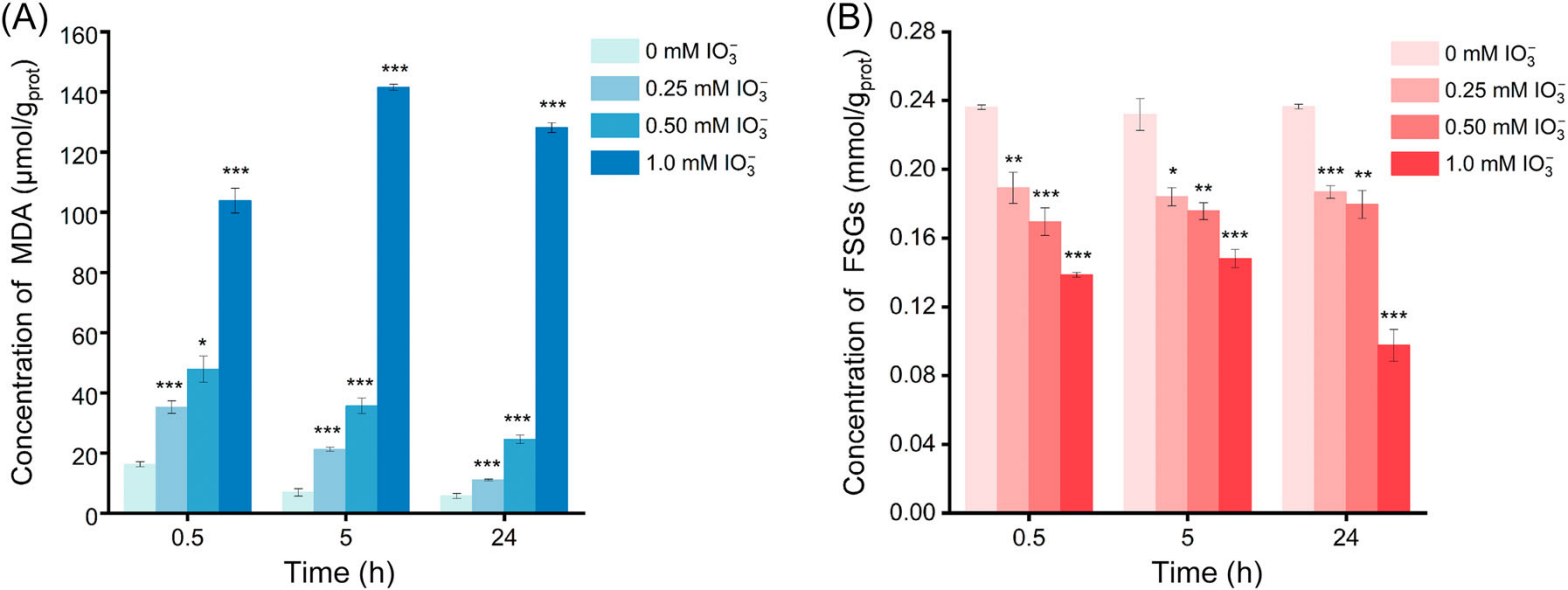
Malondialdehyde (MDA) and free sulfhydryl group (FSG) contents in *S. oneidensis* MR‐1 cells grown anaerobically in the presence of different concentrations of ${\text{IO}}_{3}^{-}$. (A) Normalized concentration of MDA in *S. oneidensis* MR‐1 cells exposed to 0, 0.25, 0.50, and 1.0 mM of ${\text{IO}}_{3}^{-}$. (B) Normalized concentration of FSGs in *S. oneidensis* MR‐1 cells exposed to 0, 0.25, 0.50, and 1.0 mM of ${\text{IO}}_{3}^{-}$. The concentrations of MDA (A) and FSGs (B) were normalized to the total protein amounts. Fumarate was supplemented as the electron acceptor as described in Figure 4B. Prot, protein. Statistical method: Student's *t*‐test. ns, *p* > 0.05; 0.01 < *p < 0.05; 0.001 < **p < 0.01; ***p < 0.001.

Under anaerobic conditions, ${\text{IO}}_{3}^{-}$ exposure induced dose‐dependent increases in MDA accumulation (Figure 5A). However, in cultures containing 0.25 mM and 0.50 mM ${\text{IO}}_{3}^{-}$, the MDA levels showed a gradual decline over a 24‐h period. Correspondingly, FSG levels showed an inverse relationship with ${\text{IO}}_{3}^{-}$ concentration (Figure 5B), where higher ${\text{IO}}_{3}^{-}$ levels resulted in greater depletion of cellular sulfhydryl groups. These results revealed that ${\text{IO}}_{3}^{-}$ exposure caused dose‐dependent oxidative damage in *S. oneidensis* MR‐1 cells.

### Transcriptomic response to ${\mathrm{IO}}_{3}^{-}$ stress in *S. oneidensis* MR‐1

To elucidate the transcriptional response of *S. oneidensis* MR‐1 to ${\text{IO}}_{3}^{-}$ stress, a comparative RNA‐seq analysis was performed on cells harvested during the initial (0 h) and fast (5 h) phases of ${\text{IO}}_{3}^{-}$ reduction (Figure S1). Genome‐wide transcriptomic profiling identified 4302 expressed genes (Table S2), with distinct transcriptional patterns between the two metabolic phases (Figure S3A). Differential expression analysis identified 55 significantly upregulated and 5 downregulated genes during the fast reduction phase compared to the initial phase (fold change > 2, *p* < 0.05; Figure S3B). Notably, the metal‐reducing genes *mtrCAB* and *omcA* showed coordinated upregulation (Table S3). The *dmsFAGH*, *so4357*–*so4358*, and *so4360*−*so4362* genes displayed minimal or non‐significant variations, whereas *dmsB* was significantly upregulated, in contrast to the downregulation of *dmsE* and *so4359* (Table S3). These results exhibited divergent expression patterns for genes in the *dmsEFABGH* operon and its homologous gene cluster (*so4357* − *so4362*).

Among 18 candidate genes selected for quantitative reverse transcription polymerase chain reaction (qRT‐PCR) validation based on their elevated transcript levels, nine genes showed consistent upregulation (*tsaA*, *sodB*, *katG*, *ahpC*, *so0534*, *so1563*, *so1659*, *so2052*, and *so3420*; Tables 1 and S4). The antioxidative functions of *tsaA*, *sodB*, *katG*, *ahpC*, and *so1563* have been well‐established in bacterial oxidative stress responses^31^, ^32^, ^33^. The protein products encoded by *so0534*, *so1659*, *so2052*, and *so3420* were predicted to encode an ACR3‐family arsenate permease, decaheme cytochrome *c* lipoprotein, flavin adenine dinucleotide (FAD)‐dependent oxidoreductase, and monoheme cytochrome *c* (Table S4).

**Table 1:** Transcriptional changes of nine genes during the fast ${\text{IO}}_{3}^{-}$ reduction phase relative to the initial phase.

Gene name	log_2_FC^a^	Ratio of relative expression^b^ (mean ± SD)	Description
*so0534*	2.10	1.65 ± 0.16*	ACR3‐family arsenate permease
*ahpC*	1.20	1.26 ± 0.00*	Alkyl hydroperoxide reductase peroxiredoxin component
*so1563*	7.02***	2.78 ± 0.12**	Cytoplasmic glutathione peroxidase, CgpD
*so1659*	2.26***	2.53 ± 0.35*	Surface localized decaheme cytochrome *c* lipoprotein
*so2052*	5.97**	10.55 ± 0.62***	FAD‐dependent oxidoreductase
*tsaA*	2.73***	3.77 ± 0.46**	Peroxiredoxin
*sodB*	1.55*	3.27 ± 0.12**	Fe/Mn superoxide dismutase
*so3420*	11.51***	3.21 ± 0.13*	Monoheme cytochrome *c*
*katG*	1.70	2.67 ± 0.00**	Catalase/peroxidase HPI

^a^Detected by transcriptomic analysis; ^b^detected by qRT‐PCR. Statistical method: Exact test (edgeR) for transcriptomic analysis; Student's *t*‐test for qRT‐PCR. 0.01 < *p < 0.05; 0.001 < **p < 0.01; ***p < 0.001. FAD, flavin adenine dinucleotide; FC, fold change; HPI, hydroperoxidase I; SD, standard deviation.

### Impaired ${\mathrm{IO}}_{3}^{-}$ reduction and H_2_O_2_ degradation in gene‐deletion mutants

**Figure 6:**
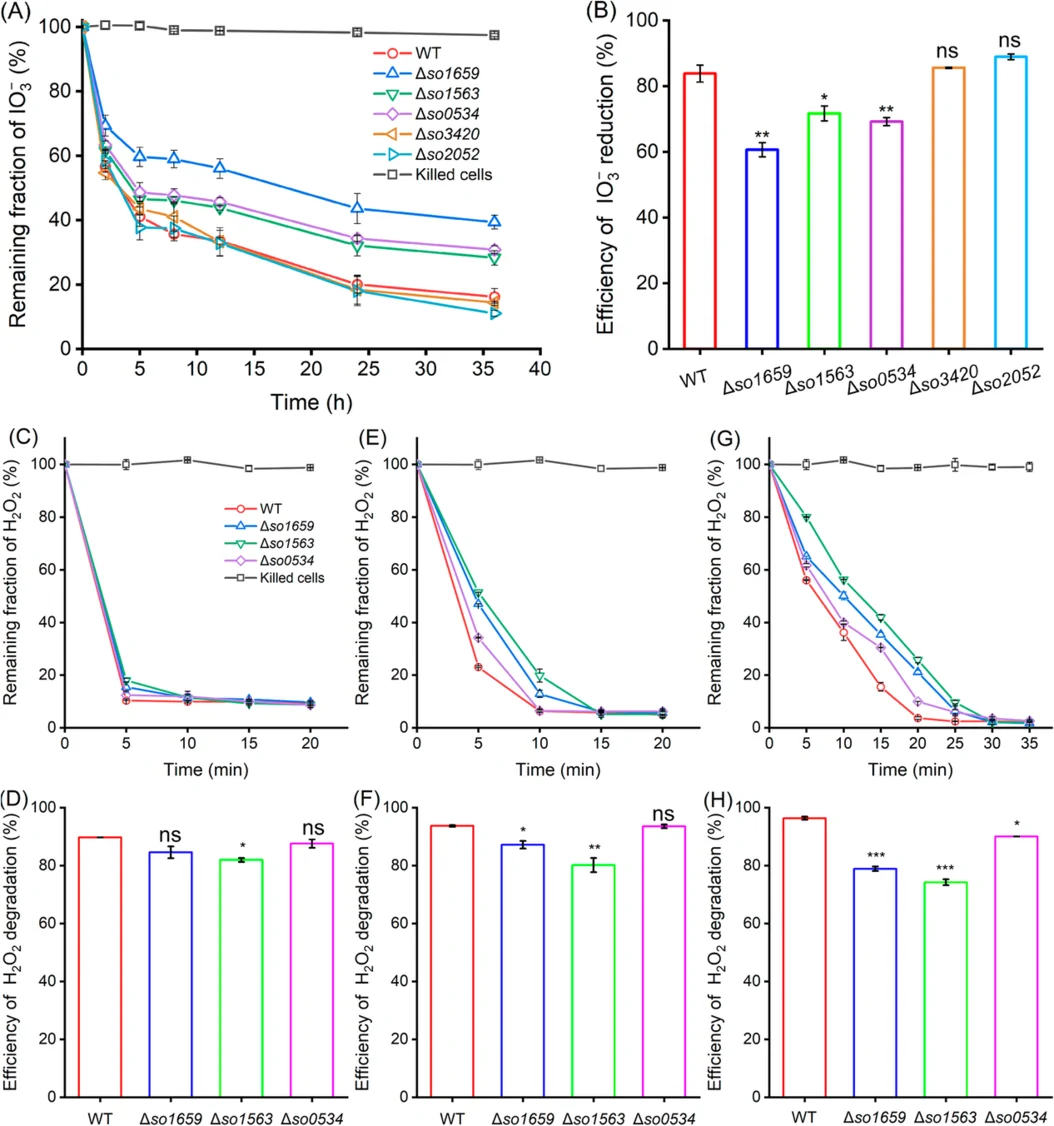
${\text{IO}}_{3}^{-}$ reduction and H_2_O_2_ degradation by mutants and wild type of *S. oneidensis* MR‐1. (A) ${\text{IO}}_{3}^{-}$ reduction traces of Δ*so1659*, Δ*so1563*, Δ*so0534*, Δ*so3420*, and Δ*so2052*. (B) Efficiency of ${\text{IO}}_{3}^{-}$ reduction at 36 h. One hundred percent of ${\text{IO}}_{3}^{-}$ was equal to 0.25 mM. (C, E, G) H_2_O_2_ degradation traces of Δ*so1659*, Δ*so1563*, and Δ*so0534* at 50 μM (C), 100 μM (E), and 200 μM (G) of initial H_2_O_2_. (D, F, H) Efficiency of H_2_O_2_ degradation at 5 min for 50 μM H_2_O_2_ (D), 10 min for 100 μM H_2_O_2_ (F), and 20 min for 200 μM H_2_O_2_ (H). Statistical method: Student's *t*‐test. ns, *p* > 0.05; 0.01 < *p < 0.05; 0.001 < **p < 0.01; ***p < 0.001.

Five targeted deletion mutants of *S. oneidensis* MR‐1, including Δ*so0534*, Δ*so1563*, Δ*so1659*, Δ*so2052*, and Δ*so3420*, were successfully constructed to investigate their functional roles in ${\text{IO}}_{3}^{-}$ reduction. Phenotypic characterization revealed that all the mutants exhibited growth kinetics comparable to the wild‐type strain (WT) under aerobic, fumarate‐respiring, and DMSO‐respiring conditions (Figure S4). Kinetic analysis of ${\text{IO}}_{3}^{-}$ reduction demonstrated that Δ*so2052* and Δ*so3420* maintained reduction rates similar to WT (Figure 6A), whereas Δ*so0534*, Δ*so1563*, and Δ*so1659* showed significantly impaired reduction kinetics. After 36 h, these three mutants achieved only 60.6%−71.7% reduction efficiency compared to WT (83.8%) (Figure 6B). Genetic complementation fully restored the ${\text{IO}}_{3}^{-}$ reduction capacity in all affected mutants (Figure S5).

Quantitative analysis showed significantly lower H_2_O_2_ accumulation during ${\text{IO}}_{3}^{-}$ reduction by WT, Δ*so0534*, and Δ*so1659* compared to that in the Δ*mtrCAB* strain, while Δ*so1563* exhibited moderate accumulation of H_2_O_2_ at 10 min (Figure S6). Subsequent H_2_O_2_ degradation assays demonstrated concentration‐dependent clearance capabilities in all strains (Figure 6C–H). While WT achieved 89.7%–96.3% degradation of 50, 100, and 200 μM H_2_O_2_ within 5, 10, and 20 min, respectively, the mutants displayed slower degradation kinetics with the following order: WT ≥ Δ*so0534* ≥ Δ*so1659* > Δ*so1563*. Specifically, the mutants exhibited significantly lower 20‐min degradation efficiency for 200 μM H_2_O_2_ (*p* < 0.05 or *p* < 0.001, Figure 6G,H).

**Figure 7:**
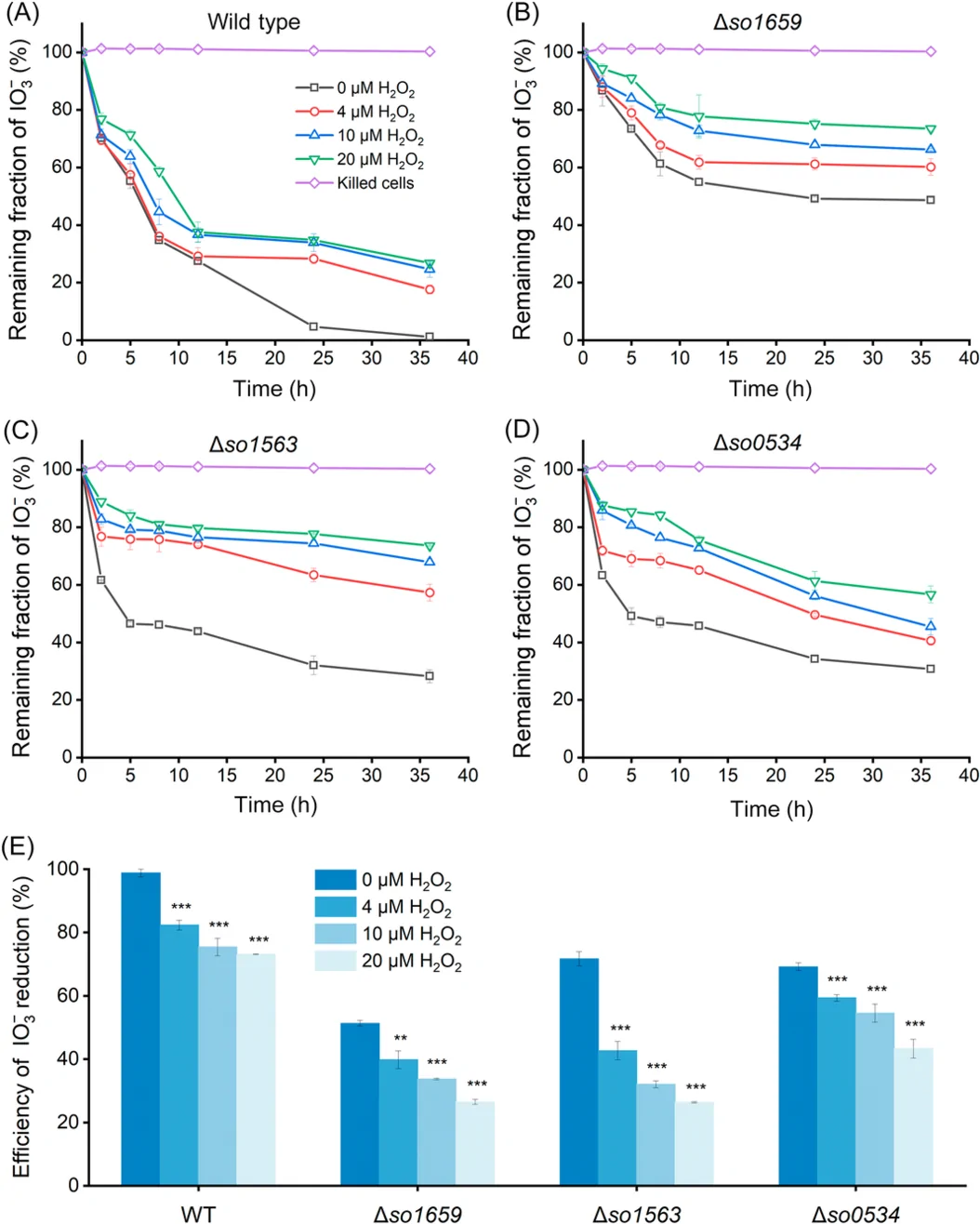
Effects of H_2_O_2_ on ${\text{IO}}_{3}^{-}$ reduction by mutants and wild type of *S. oneidensis* MR‐1. (A) ${\text{IO}}_{3}^{-}$ reduction traces of wild type in the presence of 0, 4, 10, and 20 μM of H_2_O_2_. (B) ${\text{IO}}_{3}^{-}$ reduction traces of Δ*so1659* in the presence of 0, 4, 10, and 20 μM of H_2_O_2_. (C) ${\text{IO}}_{3}^{-}$ reduction traces of Δ*so1563* in the presence of 0, 4, 10, and 20 μM of H_2_O_2_. (D) ${\text{IO}}_{3}^{-}$ reduction traces of Δ*so0534* in the presence of 0, 4, 10, and 20 μM of H_2_O_2_. (E) Efficiency of ${\text{IO}}_{3}^{-}$ reduction at 36 h. One hundred percent of ${\text{IO}}_{3}^{-}$ was equal to 0.25 mM. Statistical method: Student's *t*‐test. **0.001 < *p* < 0.01; ***p < 0.001.

Exogenous H_2_O_2_ supplementation (4−20 μM) dose‐dependently inhibited ${\text{IO}}_{3}^{-}$ reduction in all strains (Figure 7). Notably, Δ*so1563* showed the most pronounced sensitivity to H_2_O_2_, with reduction efficiency declining by 29.0%−45.3% (Figure 7E), further supporting the critical role of SO1563 in oxidative stress management during ${\text{IO}}_{3}^{-}$ reduction. In addition, the Δ*so1659* and Δ*so0534* strains exhibited H_2_O_2_ sensitivity comparable to that of WT, accompanied by a 9.9%−25.9% loss in ${\text{IO}}_{3}^{-}$ reduction efficiency.

## DISCUSSION

*Shewanella*‌ spp. are recognized for their metabolic versatility in utilizing a wide range of organic and inorganic compounds as terminal electron acceptors during anaerobic respiration^34^. Previous investigations revealed that ‌although‌ these bacteria ‌demonstrated the capability to reduce ${\text{IO}}_{3}^{-}$‌, the concentrations‌ they could reduce ‌‌effectively were substantially lower‌ than those utilized by known DIRMs^7^, ^17^. ‌Furthermore‌, ${\text{IO}}_{3}^{-}$ reduction by *S. oneidensis* MR‐1 ‌did not appear to be an inducible metabolic process‌, as evidenced by a ‌significant decrease‌ in the reduction rate following replenishment of ${\text{IO}}_{3}^{-}$ after depletion, and‌ the divergent or even inverse expression changes of *dms* genes ‌during‌ ${\text{IO}}_{3}^{-}$ reduction. ‌ In *S. oneidensis* MR‐1, the DmsEFAB complex ‌constitutes‌ an essential electron transfer pathway for the reduction of both ${\text{IO}}_{3}^{-}$ and DMSO^19^, ^20^, ^24^, ^27^. The expression of *dmsAB* was also not significantly induced during DMSO reduction^35^. These observations suggest that the DmsEFAB complex is constitutively expressed in *S. oneidensis* MR‐1 under anaerobic conditions.

Recently, the DMSO reductase family has been classified as the MopB superfamily, whose members bind molybdopterin or tungstopterin bis (pyranopterin guanine dinucleotide) (Mo/W‐bisPGD) as a cofactor^36^. The MopB enzymes catalyze the transformation of various substrates such as CO_2_, formate, sulfur and nitrogen oxides, arsenate, selenate, chlorate, and ${\text{IO}}_{3}^{-}$. Both DmsA and IdrA are members of the MopB superfamily. Whereas DmsA uses a serine residue to coordinate Mo/W‐*bis*PGD, IdrA does not employ an amino acid ligand for Mo/W‐bisPGD coordination^36^. Phylogenetically, DmsA is part of a serine‐ligated Mo/W‐bisPGD enzyme family with catalytic versatility toward sulfur and nitrogen oxides^37^. The discovery of ${\text{IO}}_{3}^{-}$ reduction activity in DmsA not only broadens the substrate specificity of this family but also adds a new member to the enzyme families involved in halogen biogeochemical cycling.

The redox potential of ${\text{IO}}_{3}^{-}$ (${\text{IO}}_{3}^{-}$/$I^{-}$, *E*°′ = +0.720 V) is higher than that of DMSO (DMSO/DMS, *E*°′ = +0.670 V)^13^, ^38^. ‌Consistent with this difference in redox potential, the calculated Gibbs free energy change (Δ*G*°′) for lactate oxidation coupled to ${\text{IO}}_{3}^{-}$ reduction (−547 kJ/mol lactate, Equation 1) ‌is‌ also ‌more favorable thermodynamically‌ than that for DMSO reduction (−384 kJ/mol lactate, Equation 2) under anaerobic conditions. However, unlike its ‌capability‌ with DMSO^24^, *S. oneidensis* MR‐1 ‌could not grow‌ using ${\text{IO}}_{3}^{-}$ as the ‌sole‌ electron acceptor. Consequently,‌ the ‌inability‌ of *S. oneidensis* MR‐1 to grow ‌via‌ ${\text{IO}}_{3}^{-}$ reduction suggests‌ that *Shewanella* spp. may have initially evolved‌ DMSO‐respiring capability, ‌with the capacity‌ for ${\text{IO}}_{3}^{-}$ reduction arising secondarily, potentially due to‌ catalytic promiscuity of DmsA. ‌Moreover,‌ the reduced tolerance of the ‌Δ*dmsEFAB* strain ‌to‌ ${\text{IO}}_{3}^{-}$ ‌demonstrates‌ that ${\text{IO}}_{3}^{-}$ reduction ‌serves‌ a detoxification ‌role‌ in *S. oneidensis* MR‐1 (Figure 4C), which is further supported by‌ the ‌observed‌ time‐dependent decline in ‌MDA‌ levels during ${\text{IO}}_{3}^{-}$ reduction by the WT (Figure 5A).

(1) $$3C_{3}H_{6}O_{3}+2{\text{IO}}_{3}^{-}\to 3C_{2}H_{4}O_{2}+3{\text{CO}}_{2}+2I^{-}+3H_{2}O(\Delta G{}^{\circ}\prime =-547\;\text{kJ}/\text{mol lactate})$$

(2) $$C_{3}H_{6}O_{3}+2C_{2}H_{6}\text{OS}\to C_{2}H_{4}O_{2}+{\text{CO}}_{2}+2C_{2}H_{6}S+H_{2}O(\Delta G{}^{\circ}\prime =-384\;\text{kJ}/\text{mol lactate})$$

Consistent with the observed growth inhibition by ${\text{IO}}_{3}^{-}$, *S. oneidensis* MR‐1 experienced significant oxidative stress ‌under ${\text{IO}}_{3}^{-}$‐reducing condition‌s, ‌as indicated by‌ elevated ‌MDA‌ levels and reduced ‌FSG content (Figure 5). ‌This observation is consistent with the strong oxidizing nature‌ of both ${\text{IO}}_{3}^{-}$ and its reduction intermediates (‌e.g.,‌ HIO and H_2_O_2_). ‌Notably,‌ *S. oneidensis* MR‐1 ‌is hypersensitive to H_2_O_2_,‌ ‌with‌ concentrations as low as 1.25 mM ‌proving lethal^32^. ‌In contrast,‌ DIRM members ‌such as‌ *Pseudomonas* ‌spp.‌ ‌exhibit tolerance to‌ H_2_O_2_ concentrations ‌exceeding‌ 22 mM^39^. ‌Therefore, the capability for growth using ${\text{IO}}_{3}^{-}$ as an electron acceptor correlates with‌ the bacterial ‌tolerance to‌ oxidative stress.

Previous studies have ‌proposed‌ that the MtrCAB complex ‌participates‌ in the detoxification of extracellular H_2_O_2_ ^20^. ‌This proposal aligns with the observed upregulation‌ of *mtrCAB* transcription ‌during‌ ${\text{IO}}_{3}^{-}$ reduction. ‌Similarly,‌ the transcript levels of *so0534*, *so1563*, and *so1659* ‌were elevated (Table 1), and their‌ deletions significantly impaired‌ both ${\text{IO}}_{3}^{-}$ reduction and H_2_O_2_ degradation in‌ *S. oneidensis* MR‐1 (Figure 6). These results ‌indicate‌ that *so0534*, *so1563*, and *so1659* function in mitigating oxidative damage‌ under ${\text{IO}}_{3}^{-}$ exposure. ‌The proteins encoded by *so0534*, *so1563*, and *so1659* ‌are predicted to localize to the inner membrane‌,‌ cytoplasm‌, and ‌extracellular compartment‌, respectively^40^. ‌The *c*‐type cytochrome SO1659 ‌may facilitate oxidant reduction via extracellular electron transfer, potentially analogous to‌ the mechanism employed by MtrCAB. As uncharged and relatively small molecules, both HIO and H_2_O_2_ can enter bacterial cells by passive diffusion, which is consistent with the observed upregulation of genes encoding intracellular antioxidant components. In *S. oneidensis* MR‐1, the OxyR and OhrR regulons constitute the primary antioxidant system, including multiple enzymes such as catalase, peroxidase, and alkyl hydroperoxide reductase^32^, ^33^, ^41^. The ‌limited‌ or ‌undetectable‌ accumulation of H_2_O_2_ ‌observed in‌ ${\text{IO}}_{3}^{-}$‐reducing cultures of mutant strains ‌likely reflects compensatory activity from alternative antioxidant enzymes.

The *so0534* gene encodes an ACR3‐family ‌arsenate permease‌, which confers resistance to pentavalent ‌arsenate‌ in *S. oneidensis* MR‐1^42^. However, ACR3 family members ‌primarily mediate‌ the extrusion of trivalent ‌arsenite‌ from cells^43^. ‌Consistent with the findings of this study‌, the transcript level of *so0534* was also elevated‌ in cells utilizing oxygen, Fe(III) citrate, Fe(III) oxide, or Co(III)‐EDTA as electron acceptors ‌relative to fumarate‌^35^, as well as during‌ heat shock response^44^. ‌These data suggest‌ that SO0534 ‌participates in diverse cellular processes beyond arsenate resistance‌. ‌Although the ACR3 family is known to be ‌evolutionarily related to the ‌bile acid:Na^+^ symporter and ‌riboflavin transporter families^45^, the functional versatility of SO0534 has not been fully elucidated based on current knowledge.

Most natural iodine at the Earth's surface resides in the ocean, ‌predominantly‌ as ${\text{IO}}_{3}^{-}$‌. ‌In contrast to‌ *S. oneidensis* MR‐1, ‌the seawater‐derived isolates‌ *S. livingstonensis* and *S. piezotolerans* exhibited increased ‌protein ‌yields with ${\text{IO}}_{3}^{-}$ as ‌the‌ electron acceptor (Figure 3C,D). ‌This observation aligns with‌ the ‌reported‌ growth^7^ of ‌the marine strain *S. putrefaciens* MR‐4 utilizing‌ ${\text{IO}}_{3}^{-}$. ‌Collectively, these findings indicate‌ that marine *Shewanella* ‌spp.‌ ‌have evolved adaptations to mitigate‌ the oxidative stress ‌imposed by‌ ${\text{IO}}_{3}^{-}$ and its reduction intermediates, ‌harnessing the energy‌ released from ${\text{IO}}_{3}^{-}$ reduction ‌for biomass production. However, ‌the ${\text{IO}}_{3}^{-}$ concentrations supporting growth of these marine *Shewanella* strains‌ remained below 1.0 mM.

In summary, this study establishes that ${\text{IO}}_{3}^{-}$ reduction in *S. oneidensis* MR‐1 serves as a detoxification mechanism against this oxidative compound. The pronounced oxidative stress induced by ${\text{IO}}_{3}^{-}$ and its reduction intermediates completely arrested bacterial growth when ${\text{IO}}_{3}^{-}$ served as the sole terminal electron acceptor. Anaerobic exposure to ${\text{IO}}_{3}^{-}$ significantly upregulated the transcript levels of both the *mtrCAB* operon and several genes encoding antioxidant defense systems. Functional characterization identified three key proteins, including glutathione peroxidase (SO1563), *c*‐type cytochrome (SO1659), and arsenate permease (SO0534), as essential components for maximum reduction of ${\text{IO}}_{3}^{-}$ and degradation of H_2_O_2_. Notably, exogenous H_2_O_2_ supplementation exhibited concentration‐dependent impairment of ${\text{IO}}_{3}^{-}$ reduction efficiency. Compared to their freshwater counterpart *S. oneidensis* MR‐1, marine‐adapted *Shewanella* species possess superior tolerance to ${\text{IO}}_{3}^{-}$ stress and have evolved the capacity to sustain protein production with low concentrations of ${\text{IO}}_{3}^{-}$ as the sole electron acceptor.

## MATERIALS AND METHODS

### Bacterial strains and culture conditions

The bacterial strains used in this study are listed in Table S1. *S. oneidensis* MR‐1 and its mutants were routinely cultured aerobically in lysogeny broth (LB) medium at 30°C. *S. livingstonensis* CCTCC AB2020017 and *S. piezotolerans* MCCC 1A01966 were obtained from the China Center for Type Culture Collection and the Marine Culture Collection of China, respectively. *S. livingstonensis* and *S. piezotolerans*, originally isolated from seawater, were routinely cultured in marine medium 2216E at 20°C (Hopebio). *E. coli* strains were utilized for plasmid construction, propagation, extraction, and conjugation experiments, and were grown in LB medium at 37°C. *E. coli* WM3064, an auxotrophic strain requiring 2,6‐diaminopimelic acid (DAP), was cultured in LB supplemented with 100 μg/ml DAP. For selection and maintenance of antibiotic‐resistant strains, gentamicin (15 μg/ml) or kanamycin (50 μg/ml) was added to the growth medium. Lactate served as the electron donor. Fumarate and ${\text{IO}}_{3}^{-}$ were employed as electron acceptors. All chemicals used in this study were of analytical or chromatographic grade.

### ${\mathrm{IO}}_{3}^{-}$ reduction assay

The ${\text{IO}}_{3}^{-}$ reduction activity of bacterial strains was assessed in anoxic, modified M1 medium, prepared as previously described^27^. The basal M1 medium contains 30 mM PIPES, 7.5 mM NaOH, 28.04 mM NH_4_Cl, 1.34 mM KCl, 4.35 mM NaH_2_PO_4_, and 1.5 mM Na_2_SO_4_, supplemented with trace minerals, vitamins, and amino acids^46^, ^47^, ^48^. To eliminate HIO, phenol was optionally supplemented to the medium. For the marine *Shewanella* strains, *S. livingstonensis* and *S. piezotolerans*, the medium was further supplemented with 2% NaCl. Lactate (20 mM) was provided as the sole electron donor. ${\text{IO}}_{3}^{-}$ was added as the terminal electron acceptor at final concentrations of 0.25, 0.50, and 1.0 mM, respectively. Bacterial cells were pre‐cultured aerobically in LB medium to mid‐exponential phase, harvested by centrifugation, washed twice with anoxic M1 base (lacking electron donor/acceptor), and resuspended in anoxic M1 medium. Cultures were inoculated in anaerobic bottles, which were sealed with butyl rubber stoppers and aluminum crimp seals. The initial OD_600_ is 0.1 unless otherwise specified. Incubation proceeded at 30°C or 20°C (for marine strains) with gentle shaking. Heat‐killed cells served as negative controls. At designated time points, the concentration of ${\text{IO}}_{3}^{-}$ in the medium was quantified spectrophotometrically using the ${\text{IO}}_{3}^{-}$‐triiodide method^17^, ^27^, ^49^. All cell washing and medium inoculation steps were performed within an anaerobic chamber (Coy Laboratory Products) under an atmosphere of mixed gases (N_2_:CO_2_:H_2_ = 95.5:3:1.5). To investigate the competition between ${\text{IO}}_{3}^{-}$ and DMSO as terminal electron acceptors, the reduction of ${\text{IO}}_{3}^{-}$ (0.25 mM) was monitored in the presence of DMSO at concentrations of 0.10, 0.25, 1.0, and 5.0 mM. Additionally, the impact of H_2_O_2_ stress on ${\text{IO}}_{3}^{-}$ (0.25 mM) reduction was examined by supplementing cultures with 4, 10, and 20 μM H_2_O_2._

### Growth measurements

To investigate the growth of *Shewanella* strains coupled to ${\text{IO}}_{3}^{-}$ reduction, cells were inoculated into anaerobic bottles containing anoxic M1 medium (as performed in the ${\text{IO}}_{3}^{-}$ reduction assay), but with a lower starting OD_600_ of 0.02. ${\text{IO}}_{3}^{-}$ was added as the sole electron acceptor with final concentrations of 0.25 and 0.50 mM. Negative controls lacked any added electron acceptor, while positive controls contained 10 mM fumarate. The bottles were incubated at 30°C with shaking at 150 rpm. Growth was monitored periodically by measuring OD_600_. Cells were harvested by centrifugation at selected time points for the determination of total cellular protein content. Cell pellets were lysed using Cell Lysis Buffer (Beyotime), and the total protein concentrations were determined using the Micro BCA Protein Assay Kit (Thermo Fisher Scientific), following the manufacturer's instructions and using bovine serum albumin as the standard.

The growth of bacterial strains was also assessed in oxic or anoxic conditions using culture tubes containing 15 ml M1 medium supplemented with lactate (20 mM). For anaerobic growth, fumarate (20 mM) was provided as the terminal electron acceptor. Cultures were inoculated to an initial OD_600_ of approximately 0.1 and incubated at 30°C with shaking. OD_600_ was monitored over time. To assess the toxic effects of ${\text{IO}}_{3}^{-}$ on growth, ${\text{IO}}_{3}^{-}$ was added to the cultures (oxic or anoxic) at final concentrations of 0, 0.25, 0.5, and 1.0 mM. These toxicity tests were performed in M1 medium.

### Detection of MDA and FSGs

Wild‐type bacterial cells exposed to ${\text{IO}}_{3}^{-}$ under anaerobic conditions were harvested by centrifugation. Cell pellets were lysed as described for the protein measurement assay. MDA concentrations were determined using a Lipid Peroxidation MDA Assay Kit (Beyotime). FSG content was measured using a Free Sulfhydryl Assay Kit based on the reaction with 5,5′‐Dithiobis(2‐nitrobenzoic acid) (Beyotime).

### DMSO detection

The concentrations of DMSO were quantified using a high‐performance liquid chromatography (HPLC) system LC‐20A (Shimadzu) comprised with an Aminex HPX‐87H column (Bio‐Rad) and an ACQUITY UPLC Photodiode Array (PDA) detector (Waters). Separation was achieved under isocratic conditions using 5 mM H_2_SO_4_ as the mobile phase at a column temperature of 50°C and a flow rate of 0.5 ml/min. DMSO was detected at 210 nm. Quantification was based on a standard curve generated using known concentrations of DMSO.

### H_2_O_2_ degradation assay

The ability of bacterial strains to degrade H_2_O_2_ was tested under anaerobic conditions in phosphate‐buffered saline (PBS). H_2_O_2_ was added to PBS at final concentrations of 50, 100, and 200 μM. Cells were pre‐cultured, washed, and resuspended in anoxic PBS as described for the ${\text{IO}}_{3}^{-}$ reduction assay, with an initial OD_600_ of 0.1. At designated times, samples were withdrawn, immediately filtered (0.22 μm) to remove cells, and the filtrate was assayed for residual H_2_O_2_. The concentration of H_2_O_2_ was determined enzymatically based on the oxidation of 2,2′‐azino‐bis(3‐ethylbenzothiazoline‐6‐sulfate) (ABTS) by H_2_O_2_, catalyzed by horseradish peroxidase (HRP), leading to the formation of the radical cation ABTS^**•+**^. Briefly, 480 μl of filtrate was mixed with 10 μl of 50 mM ABTS and 10 μl of 250 μg/ml HRP. The absorbance of the reaction solution was measured at 405 nm within 5 min using a Microplate Reader (SpectraMax® 190; Molecular Devices). Quantification was based on a standard curve generated using known concentrations of H_2_O_2_. This method was also employed to detect H_2_O_2_ generated during ${\text{IO}}_{3}^{-}$ reduction experiments as previously described^27^.

### Transcriptome sequencing

For transcriptomic analysis, *S. oneidensis* MR‐1 cells were harvested at the initial phase (shortly after inoculation) and the fast ${\text{IO}}_{3}^{-}$ reduction phase of cultures containing 0.25 mM ${\text{IO}}_{3}^{-}$. Total RNA was extracted using the HiPure Universal RNA Mini Kit (Magigene). Purified RNA from three biological replicates per phase (initial and fast reduction) was pooled to create a single transcriptome sample. Two pooled samples per phase were submitted for sequencing, including rRNA depletion, cDNA library construction (stranded), and subsequent bioinformatic data processing (read quality control, alignment, quantification, and differential expression analysis) as previously described^50^. Briefly, rRNA was depleted with the Ribo‐Zero rRNA Removal Kit (Epicentre). Strand‐specific libraries were generated with the NEBNext Ultra II Directional RNA Library Prep Kit for Illumina (New England Biolabs) and sequenced on an Illumina NovaSeq 6000 platform. Low‐quality bases were trimmed with fastp. Bowtie 2 combined with NCBI RefSeq and Rfam was used to further remove rRNA reads from clean reads. The remaining reads were mapped to the *S. oneidensis* MR‐1 reference genome with Bowtie 2. Read counts per gene were quantified by RSEM and tximport in the R environment. Differential expression analysis was performed with edgeR. The analysis pipeline employed in this study is available on the cloud computing platform‌ of the Maigene company. The *S. oneidensis* MR‐1 reference genome (NC_004347) was used for alignment. The transcriptomic data have been deposited in the NCBI Sequence Read Archive (SRA) under BioProject ID PRJNA1273913. An additional file detailing the transcriptional changes of all genes between the fast reduction phase and the initial phase is provided (Table S2).

### Quantitative RT‐PCR

Total RNA was independently isolated from cells sampled during ${\text{IO}}_{3}^{-}$ reduction assays using MiniBEST Universal RNA Extraction Kit (TaKaRa). Contaminating genomic DNA was removed by DNase I treatment, and the reverse transcription was performed using PrimeScript™ 1st Strand cDNA Synthesis Kit (TaKaRa). The resulting cDNA served as the template for quantitative analysis of specific target gene mRNA levels. The reactions were conducted on a QuantStudio^TM^ 3 (Thermo Fisher Scientific) using TB Green Premix Ex Taq II kit (Takara). Amplification reactions were performed in triplicate for each sample. The relative quantification of target gene mRNA levels was calculated using the comparative ΔΔ*C*_t_ method. The 16S rRNA gene was used as the endogenous reference gene for normalization as previously described^51^, ^52^. The induction ratio (fold‐change) in the fast reduction phase relative to the initial phase was calculated as $2^{-∆∆C_{t}}$, where ΔΔ*C*_t_ = (*C*_t, target gene_ − *C*_t, 16S rRNA_) _fast stage_ − (*C*_t, target gene_ − *C*_t, 16S rRNA_) _initial stage_. The sequences of the qRT‐PCR primers are listed in Table S5.

### Gene deletion and complementation

Target gene deletions in *S. oneidensis* MR‐1 were generated using an allelic exchange strategy based on the suicide vector pDS3.2 and a two‐step selection/counter‐selection process as described previously^27^, ^53^. Briefly, the upstream and downstream flanking regions of the target gene were amplified and fused via cross‐over PCR. The resulting fusion fragment was digested with appropriate restriction enzymes and ligated into the similarly digested suicide vector pDS3.2. The recombinant plasmid was first transformed into the donor strain *E. coli* WM3064 and introduced into *S. oneidensis* MR‐1 by conjugation. Transconjugants resulting from single‐crossover integration of the suicide plasmid into the *S. oneidensis* MR‐1 chromosome were selected on LB plates containing gentamicin. And subsequent resolution of the integrated plasmid was performed by sucrose counter‐selection. Putative deletion mutants were screened by colony PCR using primers flanking the deletion site and verified by DNA sequencing. Primers used for mutant construction are listed in Table S5.

To complement the mutants, the full‐length coding sequences of the target genes, including the ribosomal binding sites, were amplified by PCR and cloned into the broad‐host‐range expression vector pBBR1MCS‐2^54^. The recombinant plasmid was first transformed into *E. coli* DH5α and verified by PCR. Positive plasmid DNA was purified and introduced into the respective mutants via electroporation using a Bio‐Rad MicroPulser^TM^. Electroporation conditions were: 0.75 kV, 25 µF capacitance, 400 Ω resistance, using 0.1 cm gap electroporation cuvettes. The empty pBBR1MCS‐2 vector was also transformed into the WT and the mutants as controls for complementation experiments.

### Statistical analysis

All experiments described in this study were performed with at least three independent biological replicates. The values are presented as means ± standard deviations. Statistical significance of differences between experimental groups was evaluated using the Student's *t*‐test. A *p* < 0.05 was considered statistically significant.

## AUTHOR CONTRIBUTIONS

**Ziyi Wang**: Investigation; formal analysis; writing—review and editing. **Lingyu Hou**: Validation; formal analysis. **Yidan Hu**: Writing—review and editing. **Zhou Jiang**: Conceptualization; writing—review and editing. **Yiran Dong**: Conceptualization; resources. **Liang Shi**: Resources; supervision. **Yongguang Jiang**: Conceptualization; funding acquisition; writing—original draft.

## ETHICS STATEMENT

No animals or humans were involved in this study.

## CONFLICT OF INTERESTS

The authors declare no conflict of interests.

## Supporting information

Supporting File 1.

Supporting File 2.

## Data Availability

The transcriptomic data from this investigation were submitted to the NCBI BioProject Database with BioProject ID PRJNA1273913.
