# Supplementary material for: Elucidating the physiological significance of iodate reduction in Shewanella species
Source: mLife. 2026 Aug 23;5(4):507–19. doi: 10.1002/mlf2.70096 (PMC13501091; doi:10.1002/mlf2.70096)
Supplement: Supplementary file 1 [file MLF2-5-507-s001.pdf]

Table S2 Transcriptional changes of genes during the fast iodate-reduction phase relative to the initial phase. FC, fold change; FDR, false discovery rate. Statistical method: Exact test (edgeR).

Locus tag (GenBank No. NC_004347)	Old locus tag	$\log_2$FC	p value	FDR	Locus tag (GenBank No. NC_004347)	Old locus tag	$\log_2$FC	p value	FDR
SO_RS00005	SO_0001	0.0000	1.0000	1.0000	SO_RS11555	SO_2510	0.1015	0.9204	1.0000
SO_RS00010	SO_0002	-2.0619	0.1589	0.3618	SO_RS11560	SO_2511	1.5884	0.3184	0.5465
SO_RS00015	SO_0003	4.6935	0.0142	0.1084	SO_RS11565	SO_2512	0.8342	0.6344	0.8273
SO_RS00020	SO_0004	-0.7529	0.2890	0.5114	SO_RS11570	SO_2513	3.2842	0.0731	0.2403
SO_RS00025	SO_0005	0.0000	1.0000	1.0000	SO_RS11575	SO_2514	2.5843	0.1574	0.3596
SO_RS00030	SO_0006	4.1041	0.2293	0.4506	SO_RS11580	SO_2515	3.8033	0.2325	0.4533
SO_RS00035	SO_0007	0.0000	1.0000	1.0000	SO_RS11590	SO_2517	5.0700	0.0602	0.2185
SO_RS00040	SO_0008	-0.6949	0.5985	0.7930	SO_RS11595	SO_2518	7.8292	0.0468	0.1889
SO_RS00045	SO_0009	0.0097	0.9891	1.0000	SO_RS11600	SO_2519	6.8917	0.0657	0.2276
SO_RS00050	SO_0010	0.1817	0.8112	0.9606	SO_RS11605	SO_2520	-6.2255	0.0152	0.1113
SO_RS00055	SO_0011	-0.0849	0.9054	1.0000	SO_RS11610	SO_2523	-6.0210	0.0080	0.0844
SO_RS00060	SO_0012	5.2502	0.0105	0.0944	SO_RS11615	SO_2524	-4.9538	0.0058	0.0707
SO_RS00065	SO_0013	-1.0521	0.2371	0.4578	SO_RS11620	SO_2525	1.6077	0.3151	0.5425
SO_RS00070	SO_0014	-0.6299	0.1650	0.3705	SO_RS11630	SO_4784	-3.0389	0.1250	0.3159
SO_RS00075	SO_0015	1.1857	0.1365	0.3315	SO_RS11635	SO_2527	5.8223	0.1230	0.3141
SO_RS00080	SO_0016	-0.3109	0.8727	1.0000	SO_RS11640	SO_2528	0.0000	1.0000	1.0000
SO_RS00085	SO_0017	4.6290	0.0100	0.0934	SO_RS11645	SO_2529	-5.3164	0.0437	0.1811
SO_RS00090	SO_0018	6.1110	0.0297	0.1467	SO_RS11650	SO_2530	1.9032	0.1455	0.3437
SO_RS00095	SO_0019	-5.9312	0.0282	0.1436	SO_RS11655	SO_2532	3.2972	0.0808	0.2487
SO_RS00100	SO_0020	3.5224	0.0040	0.0596	SO_RS11660	SO_2533	-0.5082	0.5022	0.7147
SO_RS00105	SO_0021	2.1349	0.0068	0.0772	SO_RS11665	SO_2535	0.7916	0.6708	0.8548
SO_RS00110	SO_0022	2.5119	0.0105	0.0944	SO_RS11670	SO_2536	2.9718	0.0009	0.0304
SO_RS00115	SO_0023	-0.8895	0.6405	0.8317	SO_RS11675	SO_2537	-2.2426	0.0287	0.1442
SO_RS00120	SO_0024	-1.3894	0.3093	0.5360	SO_RS11680	SO_2538	3.0978	0.0699	0.2342
SO_RS00125	SO_0025	4.3647	0.1745	0.3824	SO_RS11685	SO_2539	-0.2800	0.7976	0.9502
SO_RS00130	SO_0026	4.3969	0.0226	0.1291	SO_RS11690	SO_2540	0.3918	0.7426	0.9078
SO_RS00135	SO_0027	-1.7979	0.0212	0.1268	SO_RS11695	SO_2541	0.5316	0.6231	0.8148
SO_RS00140	SO_0028	-2.5754	0.1423	0.3403	SO_RS11700	SO_2542	-0.6008	0.5752	0.7714
SO_RS00145	SO_0029	1.2788	0.1851	0.3961	SO_RS11705	SO_2543	4.1131	0.1437	0.3429
SO_RS00150	SO_0030	2.0312	0.2238	0.4440	SO_RS11710	SO_2544	-0.5062	0.8167	0.9637
SO_RS00155	SO_0031	-0.7240	0.5704	0.7690	SO_RS11715	SO_2545	-3.6695	0.1361	0.3314
SO_RS00160	SO_0032	0.6914	0.5685	0.7674	SO_RS11720	SO_2546	-2.5185	0.1790	0.3879
SO_RS00165	SO_0033	1.5289	0.1629	0.3679	SO_RS11725	SO_2547	7.8553	0.0051	0.0670
SO_RS00170	SO_0034	1.6037	0.0111	0.0973	SO_RS11735	SO_2549	-6.0232	0.0595	0.2172
SO_RS00175	SO_0035	2.6119	0.0001	0.0084	SO_RS11740	SO_2550	-3.3368	0.2477	0.4702
SO_RS00180	SO_0036	1.9907	0.0130	0.1037	SO_RS11745	SO_2551	-3.4189	0.1536	0.3532
SO_RS00185	SO_0037	4.0235	0.0119	0.0991	SO_RS11750	SO_2552	0.0000	1.0000	1.0000
SO_RS00190	SO_0038	4.9338	0.0082	0.0854	SO_RS11755	SO_2553	0.0000	1.0000	1.0000
SO_RS00195	SO_0039	-7.0803	0.0303	0.1471	SO_RS11760	SO_2554	0.0000	1.0000	1.0000
SO_RS00200	SO_0040	2.7559	0.1106	0.2953	SO_RS11780	SO_2557	1.2978	0.1990	0.4120
SO_RS00205	SO_0041	-1.2460	0.5723	0.7693	SO_RS11785	SO_2559	-3.0250	0.0678	0.2313
SO_RS00210	SO_0042	-3.1898	0.1252	0.3162	SO_RS11790	SO_2560	8.3611	0.0055	0.0688
SO_RS00215	SO_r001	-5.1782	0.0418	0.1773	SO_RS11795	SO_2561	-3.5931	0.0302	0.1471
SO_RS00220	SO_r002	-4.1343	0.1478	0.3467	SO_RS11800	SO_2562	1.1267	0.2751	0.4974
SO_RS00235	SO_0044	3.8111	0.3032	0.5280	SO_RS11805	SO_2563	-3.3739	0.0650	0.2260
SO_RS00240	SO_0045	-0.6820	0.7027	0.8790	SO_RS11810	SO_2564	-1.5146	0.1231	0.3141
SO_RS00245	SO_0046	-3.2319	0.1453	0.3437	SO_RS11815	SO_2565	-2.2651	0.1522	0.3524
SO_RS00250	SO_0047	-1.7822	0.1936	0.4062	SO_RS11820	SO_2566	-2.7653	0.0229	0.1291
SO_RS00255	SO_0048	-5.1951	0.0393	0.1731	SO_RS11825	SO_2567	0.4390	0.7292	0.8991
SO_RS00260	SO_0049	0.4152	0.4385	0.6639	SO_RS11830	SO_2569	-4.3917	0.0220	0.1279
SO_RS00265	SO_0050	0.5738	0.7245	0.8949	SO_RS11835	SO_2570	9.6049	0.0005	0.0214
SO_RS00270	SO_0052	0.9751	0.3314	0.5598	SO_RS11840	SO_2571	-5.5205	0.0285	0.1436
SO_RS00275	SO_0053	3.3467	0.0022	0.0463	SO_RS11845	SO_2572	-10.5696	0.0102	0.0938
SO_RS00280	SO_0054	0.1352	0.9438	1.0000	SO_RS11850	SO_2573	0.0058	0.9949	1.0000
SO_RS00285	SO_0055	0.0360	0.9759	1.0000	SO_RS11855	SO_2575	7.0966	0.0624	0.2215
SO_RS00290	SO_0056	0.1450	0.9300	1.0000	SO_RS11860	SO_2576	9.7306	0.0031	0.0544
SO_RS00295	SO_0057	8.2537	0.0451	0.1850	SO_RS11865	SO_2577	1.2669	0.2323	0.4533
SO_RS00300	SO_0058	7.6240	0.0508	0.1964	SO_RS11870	SO_2578	2.5029	0.2148	0.4338
SO_RS00305	SO_0059	-4.1338	0.1018	0.2828	SO_RS11875	SO_2580	-2.5078	0.2025	0.4170
SO_RS00310	SO_0060	0.0000	1.0000	1.0000	SO_RS11880	SO_2581	-0.1375	0.9252	1.0000
SO_RS00315	SO_0061	1.7959	0.3421	0.5704	SO_RS11885	SO_2582	1.8991	0.1772	0.3864
SO_RS00320	SO_0062	2.6141	0.2376	0.4582	SO_RS11890	SO_2583	1.4630	0.1397	0.3367

SO_RS00325	SO_0063	-0.4258	0.6914	0.8707
SO_RS00330	SO_0065	1.3393	0.4081	0.6371
SO_RS00335	SO_0066	-0.5403	0.4334	0.6602
SO_RS00340	SO_0067	0.3918	0.7596	0.9229
SO_RS00345	SO_0068	-3.3361	0.2303	0.4516
SO_RS00350	SO_0069	8.1265	0.0151	0.1109
SO_RS00355	SO_0070	-0.6273	0.7586	0.9218
SO_RS00360	SO_0071	-3.8235	0.0879	0.2614
SO_RS00365	SO_0072	2.9080	0.3611	0.5896
SO_RS00370	SO_0073	0.0000	1.0000	1.0000
SO_RS00375	SO_0074	-2.9577	0.2108	0.4290
SO_RS00380	SO_0075	3.7303	0.0001	0.0079
SO_RS00385	SO_0076	0.2098	0.7835	0.9418
SO_RS00390	SO_0077	5.7255	0.0488	0.1928
SO_RS00395	SO_0078	-9.8466	0.0028	0.0502
SO_RS00400	SO_0079	2.0861	0.5087	0.7208
SO_RS00405	SO_0080	2.0859	0.4341	0.6604
SO_RS00410	SO_0081	-7.4456	0.0247	0.1329
SO_RS00415	SO_0082	0.0000	1.0000	1.0000
SO_RS00420	SO_0083	-2.0854	0.1288	0.3203
SO_RS00425	SO_0084	1.2227	0.4318	0.6594
SO_RS00430	SO_0085	0.0000	1.0000	1.0000
SO_RS00435		2.0861	0.5180	0.7285
SO_RS00440	SO_0086	0.0000	1.0000	1.0000
SO_RS00445	SO_0088	2.3544	0.1616	0.3662
SO_RS00450	SO_0090	-4.0680	0.0549	0.2077
SO_RS00455	SO_0091	4.1041	0.1975	0.4103
SO_RS00460	SO_0092	0.0000	1.0000	1.0000
SO_RS00465	SO_0093	0.0000	1.0000	1.0000
SO_RS00470	SO_0095	3.3521	0.0857	0.2574
SO_RS00475	SO_0096	0.0000	1.0000	1.0000
SO_RS00480	SO_0097	1.3223	0.1162	0.3046
SO_RS00485	SO_0098	2.8634	0.0152	0.1113
SO_RS00490	SO_0101	1.3174	0.0059	0.0720
SO_RS00495	SO_0102	1.2036	0.0431	0.1792
SO_RS00500	SO_0103	2.8779	0.0024	0.0480
SO_RS00505	SO_0104	2.4777	0.0003	0.0174
SO_RS00510	SO_0105	0.2486	0.8279	0.9694
SO_RS00525	SO_0107	1.4620	0.3124	0.5397
SO_RS00530	SO_0108	-0.5974	0.5416	0.7453
SO_RS00535	SO_0109	2.0861	0.5202	0.7304
SO_RS00540	SO_0110	2.1771	0.1957	0.4075
SO_RS00545	SO_0111	3.4319	0.3315	0.5598
SO_RS00550	SO_0112	-0.9793	0.3264	0.5550
SO_RS00555	SO_0113	2.8914	0.0298	0.1468
SO_RS00560	SO_0114	7.7511	0.0047	0.0654
SO_RS00565	SO_0115	-8.5730	0.0098	0.0920
SO_RS00570	SO_0116	3.8137	0.2336	0.4540
SO_RS00575	SO_0118	-0.6981	0.3248	0.5535
SO_RS00580	SO_0119	-1.3461	0.0957	0.2733
SO_RS00585	SO_0120	2.3363	0.1981	0.4103
SO_RS00590	SO_0121	-0.3864	0.6935	0.8719
SO_RS00595	SO_0122	2.9041	0.3189	0.5471
SO_RS00600	SO_0123	1.0953	0.5161	0.7273
SO_RS00605	SO_0125	-0.9175	0.4123	0.6405
SO_RS00610	SO_0126	0.4510	0.6853	0.8654
SO_RS00615	SO_0127	-1.3287	0.1240	0.3148
SO_RS00620	SO_0129	2.2012	0.3043	0.5294
SO_RS00625	SO_0130	1.7298	0.0032	0.0552
SO_RS00630	SO_0131	2.8449	0.1104	0.2953
SO_RS00635	SO_0132	1.3505	0.1866	0.3983
SO_RS00640	SO_0134	0.3929	0.7642	0.9263
SO_RS00645	SO_0135	3.1784	0.1040	0.2860
SO_RS00650	SO_0136	1.0112	0.5746	0.7712
SO_RS00655	SO_0137	-2.1195	0.1353	0.3304
SO_RS00660	SO_0138	4.9232	0.0004	0.0200
SO_RS00665	SO_0139	0.6614	0.3446	0.5724
SO_RS00670	SO_0141	-3.2675	0.0696	0.2339

SO_RS11895	SO_2584	-5.0166	0.0443	0.1830
SO_RS11900	SO_2585	-7.6152	0.0129	0.1035
SO_RS11905	SO_2586	4.1510	0.0301	0.1469
SO_RS11910	SO_2587	0.5883	0.6737	0.8565
SO_RS11915	SO_4785	-6.2504	0.0216	0.1275
SO_RS11920	SO_2588	2.6452	0.0003	0.0149
SO_RS11925	SO_2589	-3.0843	0.1879	0.3999
SO_RS11930	SO_2590	0.6027	0.5329	0.7386
SO_RS11935	SO_2591	-0.4267	0.7214	0.8919
SO_RS11940	SO_2592	-0.1730	0.9123	1.0000
SO_RS11945	SO_2593	0.9482	0.0086	0.0868
SO_RS11950	SO_2594	-0.9618	0.3977	0.6269
SO_RS11955	SO_2595	-0.2298	0.8999	1.0000
SO_RS11960	SO_2596	0.4718	0.5602	0.7611
SO_RS11965	SO_2597	2.1358	0.0050	0.0667
SO_RS11970	SO_2598	0.0000	1.0000	1.0000
SO_RS11975	SO_2600	1.0419	0.2366	0.4575
SO_RS11980	SO_2601	0.3811	0.4392	0.6641
SO_RS11985	SO_2602	0.2708	0.6671	0.8516
SO_RS11990	SO_2603	-1.2138	0.1637	0.3685
SO_RS11995	SO_2604	-0.9361	0.4139	0.6418
SO_RS12000	SO_4786	2.0933	0.2519	0.4733
SO_RS12005	SO_2605	-0.6979	0.6872	0.8665
SO_RS12010	SO_2606	0.0806	0.9150	1.0000
SO_RS12015	SO_2607	-0.0371	0.9661	1.0000
SO_RS12020	SO_2609	-0.5847	0.7071	0.8818
SO_RS12025	SO_2610	-0.8832	0.5654	0.7649
SO_RS12030	SO_2611	-6.3383	0.0277	0.1423
SO_RS12035	SO_2612	-9.1977	0.0155	0.1122
SO_RS12040	SO_2613	6.9152	0.0634	0.2231
SO_RS12045	SO_2614	-2.2840	0.1234	0.3141
SO_RS12050	SO_2615	1.1032	0.5002	0.7138
SO_RS12055	SO_2616	-1.1236	0.2596	0.4814
SO_RS12060	SO_2617	0.4609	0.7682	0.9291
SO_RS12065	SO_2618	2.2107	0.0247	0.1329
SO_RS12070	SO_2619	1.7007	0.0176	0.1187
SO_RS12075	SO_2620	-4.8707	0.0669	0.2293
SO_RS12080	SO_2621	-0.9098	0.3450	0.5726
SO_RS12085	SO_2622	0.7943	0.5386	0.7433
SO_RS12090	SO_2623	3.7374	0.0222	0.1283
SO_RS12095	SO_2624	2.2782	0.2232	0.4435
SO_RS12100	SO_2625	3.4289	0.1922	0.4047
SO_RS12105	SO_2626	2.5965	0.0002	0.0132
SO_RS12110	SO_2627	1.4506	0.2411	0.4613
SO_RS12115	SO_2628	-4.1344	0.1107	0.2954
SO_RS12120	SO_2629	1.6453	0.0035	0.0568
SO_RS12125	SO_2630	1.7017	0.3627	0.5911
SO_RS12130	SO_2631	7.9823	0.0492	0.1938
SO_RS12135	SO_2633	-0.4226	0.7203	0.8913
SO_RS12140	SO_2634	3.0354	0.1186	0.3079
SO_RS12145	SO_2635	0.3536	0.6530	0.8414
SO_RS12150	SO_2636	9.2409	0.0019	0.0435
SO_RS12155	SO_2637	0.6570	0.4308	0.6589
SO_RS12160	SO_2638	1.6923	0.0188	0.1205
SO_RS12165	SO_2639	-0.2009	0.9033	1.0000
SO_RS12170	SO_2640	-0.6419	0.5979	0.7929
SO_RS12175	SO_2642	1.3139	0.5225	0.7313
SO_RS12180	SO_2643	0.4861	0.2988	0.5225
SO_RS12185	SO_2644	1.2007	0.0071	0.0781
SO_RS12190	SO_2645	1.0589	0.4173	0.6461
SO_RS12195	SO_2646	1.0265	0.1075	0.2903
SO_RS12200	SO_2647	0.0000	1.0000	1.0000
SO_RS12205	SO_2648	1.4955	0.1219	0.3128
SO_RS12210	SO_2649	0.2065	0.7891	0.9445
SO_RS12215	SO_2650	-1.1720	0.4604	0.6801
SO_RS12220	SO_2652	-0.2143	0.8667	0.9996
SO_RS12225	SO_2653	0.0000	1.0000	1.0000
SO_RS12230	SO_2654	-5.5541	0.0056	0.0697

SO_RS00675	SO_0142	-3.1468	0.0735	0.2405
SO_RS00680	SO_0144	5.6302	0.0021	0.0458
SO_RS00685	SO_0145	5.6307	0.1298	0.3219
SO_RS00690	SO_0146	0.0000	1.0000	1.0000
SO_RS00695	SO_0147	-2.1986	0.1363	0.3314
SO_RS00700	SO_0148	-2.3306	0.1380	0.3341
SO_RS00705	SO_0149	0.0000	1.0000	1.0000
SO_RS00710	SO_0150	-2.2610	0.0488	0.1928
SO_RS00715	SO_0151	-2.1862	0.2889	0.5114
SO_RS00720	SO_0152	-1.1819	0.3592	0.5882
SO_RS00725	SO_0154	1.8987	0.1929	0.4055
SO_RS00730	SO_0157	6.0056	0.0044	0.0623
SO_RS00735	SO_0159	1.5167	0.4844	0.6997
SO_RS00740	SO_0160	1.4359	0.4647	0.6831
SO_RS00745	SO_0162	2.0810	0.0427	0.1786
SO_RS00750	SO_0163	1.1602	0.2480	0.4703
SO_RS00755	SO_0164	-5.2048	0.0399	0.1740
SO_RS00760	SO_0165	-0.1751	0.9209	1.0000
SO_RS00765	SO_0166	-1.0751	0.2132	0.4316
SO_RS00770	SO_0167	-1.5083	0.1321	0.3257
SO_RS00775	SO_0168	-0.1343	0.9248	1.0000
SO_RS00780	SO_0169	-0.6791	0.5354	0.7405
SO_RS00785	SO_0170	-1.6806	0.1723	0.3802
SO_RS00790	SO_0171	-0.5923	0.6970	0.8743
SO_RS00795	SO_0172	-1.1816	0.4899	0.7041
SO_RS00800	SO_0173	-0.3773	0.7297	0.8994
SO_RS00805	SO_0174	-0.1978	0.8223	0.9666
SO_RS00810	SO_0175	0.2005	0.8895	1.0000
SO_RS00815	SO_0176	-0.9496	0.3671	0.5965
SO_RS00820	SO_0177	0.2533	0.8971	1.0000
SO_RS00825	SO_0179	0.0000	1.0000	1.0000
SO_RS00830	SO_0180	0.0000	1.0000	1.0000
SO_RS00835	SO_0181	2.9118	0.3095	0.5360
SO_RS00845	SO_0183	-6.8569	0.0212	0.1268
SO_RS00850	SO_0184	-2.6989	0.1598	0.3631
SO_RS00855	SO_0185	6.2974	0.0913	0.2662
SO_RS00860	SO_0186	2.9043	0.3576	0.5874
SO_RS00865	SO_0187	5.8886	0.0962	0.2745
SO_RS00870	SO_0188	8.6632	0.0171	0.1175
SO_RS00875	SO_0189	1.9378	0.0019	0.0435
SO_RS00880	SO_0190	1.5031	0.3930	0.6218
SO_RS00885	SO_0191	0.6690	0.5621	0.7619
SO_RS00890	SO_0192	-2.7201	0.1296	0.3219
SO_RS00895	SO_0193	0.1060	0.9484	1.0000
SO_RS00900	SO_0194	1.5013	0.2329	0.4533
SO_RS00905	SO_0195	-4.2926	0.0226	0.1291
SO_RS00910	SO_0196	3.0458	0.0168	0.1169
SO_RS00915	SO_0197	2.0240	0.0057	0.0706
SO_RS00920	SO_0198	-0.1279	0.9465	1.0000
SO_RS00925	SO_0201	-5.8221	0.0052	0.0673
SO_RS00930	SO_0202	-4.8060	0.0219	0.1279
SO_RS00935	SO_0203	2.5237	0.1707	0.3777
SO_RS00940	SO_0204	0.6439	0.4085	0.6371
SO_RS00945	SO_0205	-1.8851	0.2942	0.5170
SO_RS00950	SO_0206	0.2819	0.7644	0.9263
SO_RS00955	SO_0207	-3.7032	0.0931	0.2700
SO_RS00960	SO_0208	1.8089	0.2532	0.4741
SO_RS00965	SO_r004	-2.7700	0.2109	0.4290
SO_RS00980	SO_r005	-3.1695	0.2465	0.4688
SO_RS00990	SO_0211	6.5768	0.0175	0.1182
SO_RS00995	SO_0213	0.6417	0.5776	0.7738
SO_RS01000	SO_0214	0.5056	0.4499	0.6703
SO_RS01005	SO_0215	1.0504	0.2717	0.4934
SO_RS01010	SO_t005	4.3568	0.1704	0.3773
SO_RS01015	SO_t006	-4.2486	0.1030	0.2844
SO_RS01030	SO_0217	2.5199	0.0000	0.0035
SO_RS01035	SO_0218	-0.9559	0.4551	0.6753
SO_RS01040	SO_0219	-0.9215	0.1479	0.3467

SO_RS12235	SO_2655	7.5592	0.0562	0.2106
SO_RS12240	SO_2656	0.0000	1.0000	1.0000
SO_RS12245	SO_2657	5.8223	0.1322	0.3257
SO_RS12255	SO_2659	0.0000	1.0000	1.0000
SO_RS12260	SO_2660	6.0381	0.0999	0.2800
SO_RS12265	SO_2661	0.0000	1.0000	1.0000
SO_RS12270	SO_4788	0.0000	1.0000	1.0000
SO_RS12275	SO_2663	8.0545	0.0196	0.1217
SO_RS12285	SO_2664	0.0000	1.0000	1.0000
SO_RS12290	SO_2665	-3.2352	0.2422	0.4631
SO_RS12295	SO_2666	-1.3090	0.4880	0.7036
SO_RS12300	SO_2667	-1.9148	0.2739	0.4961
SO_RS12305	SO_4789	-0.4681	0.7812	0.9414
SO_RS12310	SO_2668	0.0000	1.0000	1.0000
SO_RS12320	SO_2670	1.0776	0.5183	0.7286
SO_RS12325	SO_2671	0.0000	1.0000	1.0000
SO_RS12330	SO_2672	0.0000	1.0000	1.0000
SO_RS12335	SO_2673	0.0000	1.0000	1.0000
SO_RS12345	SO_2675	0.0000	1.0000	1.0000
SO_RS12350	SO_2676	0.0000	1.0000	1.0000
SO_RS12355	SO_2677	5.0740	0.2133	0.4317
SO_RS12365	SO_2679	-4.5690	0.0600	0.2185
SO_RS12370	SO_2680	-3.5362	0.1026	0.2842
SO_RS12375	SO_2681	3.7262	0.1666	0.3720
SO_RS12380	SO_2682	4.7577	0.0994	0.2791
SO_RS12385	SO_2683	-3.0397	0.1316	0.3249
SO_RS12390	SO_2684	0.0000	1.0000	1.0000
SO_RS12395	SO_2685	5.0605	0.1275	0.3188
SO_RS12400	SO_2686	0.0000	1.0000	1.0000
SO_RS12405	SO_2687	0.0000	1.0000	1.0000
SO_RS12410	SO_2688	5.0466	0.0242	0.1313
SO_RS12415	SO_2689	7.7121	0.0079	0.0830
SO_RS12420	SO_2690	9.6953	0.0049	0.0665
SO_RS12425	SO_2691	6.6217	0.0246	0.1325
SO_RS12435	SO_2693	4.8230	0.0182	0.1199
SO_RS12440	SO_2694	0.0000	1.0000	1.0000
SO_RS12445	SO_2695	3.4229	0.3345	0.5629
SO_RS12450	SO_2697	0.8466	0.5944	0.7901
SO_RS12455	SO_2698	3.1429	0.1303	0.3226
SO_RS12460	SO_2699	7.4573	0.0264	0.1382
SO_RS12465	SO_2700	-2.4052	0.1518	0.3524
SO_RS12470	SO_2701	-8.3669	0.0322	0.1520
SO_RS12475	SO_2702	4.5648	0.1753	0.3834
SO_RS12480	SO_2703	0.0000	1.0000	1.0000
SO_RS12490	SO_2705	-1.0470	0.0367	0.1649
SO_RS12495	SO_2706	-1.8342	0.2492	0.4709
SO_RS12500	SO_2707	9.4296	0.0020	0.0453
SO_RS12505	SO_2708	-0.8132	0.3720	0.6014
SO_RS12510	SO_2710	1.1472	0.1084	0.2907
SO_RS12515	SO_2711	-2.3848	0.0578	0.2143
SO_RS12520	SO_2712	-3.4029	0.1200	0.3101
SO_RS12525	SO_2713	3.4259	0.2858	0.5091
SO_RS12530	SO_2714	2.0861	0.4827	0.6985
SO_RS12535	SO_2715	-5.8989	0.0081	0.0845
SO_RS12540	SO_2717	2.1528	0.2397	0.4605
SO_RS12545	SO_2718	8.0575	0.0159	0.1136
SO_RS12550	SO_2719	-2.6407	0.2666	0.4885
SO_RS12555	SO_2720	1.9515	0.0844	0.2554
SO_RS12560	SO_2721	4.9977	0.0143	0.1084
SO_RS12565	SO_2722	-1.5408	0.2719	0.4935
SO_RS12570	SO_2723	6.4670	0.0281	0.1436
SO_RS12575	SO_2724	3.5135	0.0043	0.0615
SO_RS12580	SO_2725	-0.2242	0.8455	0.9836
SO_RS12585	SO_2726	1.9160	0.1958	0.4075
SO_RS12590	SO_2727	-0.2500	0.7018	0.8786
SO_RS12595	SO_2728	0.9815	0.0289	0.1448
SO_RS12600	SO_2730	0.3496	0.7665	0.9284
SO_RS12605	SO_2731	2.2198	0.1353	0.3304

SO_RS01045	SO_0220	-0.2825	0.5018	0.7144
SO_RS01050	SO_0221	0.6518	0.0800	0.2470
SO_RS01055	SO_0222	0.6761	0.1366	0.3316
SO_RS01060	SO_0223	-0.3000	0.5248	0.7328
SO_RS01065	SO_0224	1.7381	0.0037	0.0583
SO_RS01070	SO_0225	1.9997	0.0001	0.0096
SO_RS01075	SO_0226	0.2775	0.6734	0.8565
SO_RS01080	SO_0227	0.7937	0.1244	0.3151
SO_RS01085	SO_0228	0.0708	0.8261	0.9683
SO_RS01090	SO_0229	1.6212	0.0008	0.0281
SO_RS01095	SO_0230	2.1886	0.0324	0.1522
SO_RS01100	SO_0231	1.2173	0.0227	0.1291
SO_RS01105	SO_0232	0.3707	0.3911	0.6202
SO_RS01110	SO_0233	-1.0751	0.2505	0.4717
SO_RS01115	SO_0234	1.7258	0.0006	0.0238
SO_RS01120	SO_0235	-0.1593	0.9056	1.0000
SO_RS01125	SO_0236	1.2079	0.1920	0.4046
SO_RS01130	SO_0237	1.3841	0.0027	0.0502
SO_RS01135	SO_0238	2.8857	0.0022	0.0463
SO_RS01140	SO_0239	-1.8251	0.2194	0.4393
SO_RS01145	SO_0240	-0.7296	0.5608	0.7613
SO_RS01150	SO_0241	-0.4451	0.5435	0.7467
SO_RS01155	SO_0242	-1.5967	0.1197	0.3095
SO_RS01160	SO_0243	-0.9902	0.0260	0.1373
SO_RS01165	SO_0244	-1.9390	0.1548	0.3552
SO_RS01170	SO_0245	-0.7438	0.3545	0.5835
SO_RS01175	SO_0246	0.1497	0.7530	0.9171
SO_RS01180	SO_0247	-1.2986	0.1441	0.3434
SO_RS01185	SO_0248	0.8762	0.1525	0.3524
SO_RS01190	SO_0249	1.8058	0.3842	0.6128
SO_RS01195	SO_0250	0.5030	0.2516	0.4728
SO_RS01200	SO_0251	0.6812	0.0424	0.1784
SO_RS01205	SO_0252	0.0000	1.0000	1.0000
SO_RS01210	SO_0253	1.0494	0.0999	0.2800
SO_RS01215	SO_0254	0.4372	0.4379	0.6639
SO_RS01220	SO_0255	0.2489	0.4412	0.6659
SO_RS01225	SO_0256	1.2888	0.0215	0.1274
SO_RS01230	SO_0257	1.2173	0.1978	0.4103
SO_RS01235	SO_0258	-4.9010	0.0618	0.2207
SO_RS01240	SO_0259	2.9182	0.0018	0.0425
SO_RS01245	SO_0260	0.0952	0.9640	1.0000
SO_RS01250	SO_0261	0.5625	0.6051	0.7987
SO_RS01255	SO_0262	2.6868	0.1042	0.2860
SO_RS01260	SO_0263	-4.1950	0.0034	0.0564
SO_RS01265	SO_0264	1.9384	0.0982	0.2771
SO_RS01270	SO_0265	-0.3333	0.7920	0.9462
SO_RS01275	SO_0266	-0.2600	0.5278	0.7339
SO_RS01280	SO_0267	-1.4762	0.1323	0.3258
SO_RS01285	SO_0268	1.0400	0.4854	0.7009
SO_RS01290	SO_0269	-4.8612	0.0048	0.0657
SO_RS01295	SO_r007	-4.1158	0.1248	0.3158
SO_RS01300	SO_r008	0.4602	0.8627	0.9974
SO_RS01310	SO_0272	2.9093	0.0239	0.1305
SO_RS01315	SO_0273	-1.7299	0.3791	0.6078
SO_RS01320	SO_0274	0.9518	0.5263	0.7333
SO_RS01325	SO_0275	4.9208	0.0736	0.2405
SO_RS01330	SO_0276	-4.1353	0.0412	0.1766
SO_RS01335	SO_0277	7.6790	0.0192	0.1206
SO_RS01340	SO_0278	2.1709	0.0470	0.1899
SO_RS01345	SO_0279	9.0810	0.0025	0.0490
SO_RS01350	SO_0280	-0.7454	0.2881	0.5113
SO_RS01355	SO_0281	-2.5936	0.0018	0.0433
SO_RS01360	SO_0282	0.4891	0.6727	0.8559
SO_RS01365	SO_0283	0.8028	0.5878	0.7843
SO_RS01370	SO_0284	5.4041	0.0043	0.0615
SO_RS01375	SO_0285	1.0552	0.0758	0.2422
SO_RS01380	SO_0286	-1.7813	0.1784	0.3876
SO_RS01385	SO_0287	-0.1967	0.8603	0.9954

SO_RS12610	SO_2732	-2.8595	0.0616	0.2205
SO_RS12620	SO_2736	-5.2429	0.0034	0.0568
SO_RS12625	SO_2737	5.5363	0.0482	0.1919
SO_RS12630	SO_2738	-10.1255	0.0117	0.0988
SO_RS12635	SO_2739	-1.4104	0.3582	0.5876
SO_RS12640	SO_2740	1.4429	0.3987	0.6277
SO_RS12645	SO_2741	-3.9308	0.0086	0.0869
SO_RS12650	SO_2742	-0.2704	0.7309	0.8999
SO_RS12655	SO_2743	0.9870	0.1755	0.3836
SO_RS12660	SO_2744	1.7734	0.3538	0.5827
SO_RS12665	SO_2745	0.2689	0.8638	0.9982
SO_RS12715	SO_2746	3.4890	0.0116	0.0987
SO_RS12720	SO_2747	1.1906	0.0779	0.2444
SO_RS12725	SO_2748	0.8085	0.1029	0.2844
SO_RS12730	SO_2749	-0.9444	0.1744	0.3824
SO_RS12735	SO_2750	0.2957	0.8381	0.9779
SO_RS12740	SO_2751	-2.3118	0.0177	0.1187
SO_RS12745	SO_2752	-3.6088	0.0756	0.2420
SO_RS12750	SO_2753	0.1337	0.8223	0.9666
SO_RS12755	SO_2754	1.3610	0.0028	0.0502
SO_RS12760	SO_2755	-1.9385	0.4020	0.6309
SO_RS12765	SO_2756	2.7287	0.0000	0.0044
SO_RS12770	SO_2757	-3.2641	0.0189	0.1205
SO_RS12775	SO_2758	-0.0513	0.9777	1.0000
SO_RS12780	SO_2759	0.0752	0.9158	1.0000
SO_RS12785	SO_2760	3.3801	0.0031	0.0546
SO_RS12790	SO_2761	0.4673	0.7873	0.9432
SO_RS12795	SO_2762	9.4793	0.0003	0.0176
SO_RS12800	SO_2763	2.3626	0.1569	0.3587
SO_RS12805	SO_2764	0.0000	1.0000	1.0000
SO_RS12810	SO_2766	8.3602	0.0066	0.0765
SO_RS12815	SO_2767	1.6037	0.0022	0.0463
SO_RS12820	SO_2768	2.3311	0.0066	0.0765
SO_RS12825	SO_2769	1.4755	0.1564	0.3578
SO_RS12830	SO_2771	1.2120	0.2896	0.5120
SO_RS12835	SO_2772	-0.0121	0.9891	1.0000
SO_RS12840	SO_2773	-6.4214	0.0155	0.1122
SO_RS12845	SO_2774	-0.5330	0.4396	0.6643
SO_RS12850	SO_2775	3.6867	0.0088	0.0869
SO_RS12855	SO_2776	0.1439	0.7567	0.9209
SO_RS12860	SO_2777	-0.2833	0.7676	0.9287
SO_RS12865	SO_2778	-2.1937	0.1994	0.4125
SO_RS12870	SO_2779	-0.6746	0.6817	0.8630
SO_RS12875	SO_2780	2.0861	0.5095	0.7212
SO_RS12880	SO_2781	-0.7304	0.1680	0.3741
SO_RS12885	SO_2782	-3.1195	0.0631	0.2230
SO_RS12890	SO_2783	0.0000	1.0000	1.0000
SO_RS12895	SO_2784	1.0294	0.2481	0.4703
SO_RS12900	SO_2785	-1.4451	0.0197	0.1217
SO_RS12910	SO_2787	-1.3864	0.0299	0.1468
SO_RS12915	SO_2788	-0.2454	0.8532	0.9893
SO_RS12920	SO_2789	0.3079	0.7170	0.8899
SO_RS12925	SO_2790	2.8030	0.0257	0.1364
SO_RS12930	SO_2791	2.2889	0.0409	0.1758
SO_RS12935	SO_2792	2.0907	0.4793	0.6966
SO_RS12940	SO_2793	-0.4779	0.7815	0.9414
SO_RS12945	SO_2794	-0.8369	0.3339	0.5624
SO_RS12950	SO_2795	-1.9523	0.2943	0.5170
SO_RS12955	SO_2796	-2.8129	0.0114	0.0981
SO_RS12960	SO_2797	0.0000	1.0000	1.0000
SO_RS12965	SO_2798	-0.7668	0.7201	0.8913
SO_RS12970	SO_2799	3.9600	0.1185	0.3078
SO_RS12975	SO_2800	0.0000	1.0000	1.0000
SO_RS12980	SO_2801	-1.2553	0.4865	0.7019
SO_RS12985	SO_2802	-2.8317	0.0236	0.1303
SO_RS12990	SO_2803	3.2714	0.1297	0.3219
SO_RS12995	SO_2804	0.0000	1.0000	1.0000
SO_RS13000	SO_2805	-9.0142	0.0147	0.1100

SO_RS01390	SO_0288	0.0667	0.9424	1.0000
SO_RS01395	SO_0289	-1.3265	0.3492	0.5769
SO_RS01400	SO_0290	0.0000	1.0000	1.0000
SO_RS01405	SO_0291	1.6282	0.3316	0.5598
SO_RS01410	SO_0292	0.9194	0.5040	0.7161
SO_RS01415	SO_0293	-0.6326	0.5991	0.7932
SO_RS01420	SO_0294	-0.0413	0.9526	1.0000
SO_RS01425	SO_0295	-9.2386	0.0019	0.0435
SO_RS01430	SO_0296	4.9266	0.1381	0.3341
SO_RS01435	SO_0297	-0.1674	0.8961	1.0000
SO_RS01440	SO_0298	5.4394	0.0003	0.0158
SO_RS01445	SO_0299	8.0915	0.0248	0.1334
SO_RS01450	SO_0300	0.6162	0.3939	0.6225
SO_RS01455	SO_0301	-2.1748	0.1888	0.4014
SO_RS01460	SO_m001	-2.3728	0.0010	0.0322
SO_RS01465	SO_0302	-3.5849	0.1208	0.3112
SO_RS01470	SO_0304	1.1690	0.2873	0.5105
SO_RS01475	SO_0306	-0.6939	0.6840	0.8646
SO_RS01480	SO_0308	1.2875	0.5613	0.7613
SO_RS01485	SO_0309	-1.3914	0.1745	0.3824
SO_RS01490	SO_0311	-2.0157	0.0551	0.2079
SO_RS01495	SO_0312	2.9042	0.3698	0.5987
SO_RS01500	SO_0313	9.1553	0.0223	0.1286
SO_RS01505	SO_0314	-2.4397	0.2292	0.4506
SO_RS01515	SO_0316	10.0157	0.0014	0.0377
SO_RS01520	SO_0317	-1.7716	0.3817	0.6106
SO_RS01525	SO_0318	7.9101	0.0186	0.1205
SO_RS01530	SO_0319	0.8938	0.3207	0.5489
SO_RS01535	SO_0320	-1.3956	0.3980	0.6272
SO_RS01540	SO_0321	-1.4813	0.2497	0.4709
SO_RS01545	SO_0322	-0.1434	0.8254	0.9683
SO_RS01550	SO_0323	1.3333	0.4083	0.6371
SO_RS01555	SO_0324	0.0000	1.0000	1.0000
SO_RS01560	SO_0325	-5.4191	0.0174	0.1182
SO_RS01565	SO_0326	2.1005	0.0158	0.1135
SO_RS01570	SO_0329	0.0000	1.0000	1.0000
SO_RS01575	SO_0330	-0.5254	0.7661	0.9282
SO_RS01580	SO_0331	0.0000	1.0000	1.0000
SO_RS01585	SO_0332	-0.7904	0.4128	0.6409
SO_RS01590	SO_0333	0.3478	0.7817	0.9415
SO_RS01595	SO_0334	-2.5278	0.0282	0.1436
SO_RS01600	SO_0335	0.6275	0.7084	0.8826
SO_RS01605	SO_0336	-1.7491	0.1324	0.3258
SO_RS01610	SO_0337	1.3862	0.4067	0.6362
SO_RS01615	SO_0338	4.5702	0.0897	0.2640
SO_RS01620	SO_0339	0.0000	1.0000	1.0000
SO_RS01625	SO_0340	4.5349	0.0007	0.0249
SO_RS01630	SO_0341	-1.1161	0.1799	0.3894
SO_RS01635	SO_0342	4.3151	0.0042	0.0605
SO_RS01640	SO_0343	0.8389	0.3232	0.5515
SO_RS01645	SO_0344	0.8365	0.1957	0.4075
SO_RS01650	SO_0345	1.7791	0.1461	0.3446
SO_RS01655	SO_0346	0.0000	1.0000	1.0000
SO_RS01660	SO_0347	2.7443	0.1187	0.3079
SO_RS01665	SO_0348	1.8656	0.3676	0.5969
SO_RS01670	SO_0349	2.0859	0.4389	0.6641
SO_RS01675	SO_0350	0.0689	0.9328	1.0000
SO_RS01680	SO_0351	-1.6980	0.4634	0.6823
SO_RS01685	SO_0352	4.3567	0.1004	0.2808
SO_RS01690	SO_0353	-2.4454	0.2030	0.4176
SO_RS01695	SO_0354	-2.6202	0.0924	0.2687
SO_RS01700	SO_0355	2.0281	0.0300	0.1469
SO_RS01705	SO_0356	1.1738	0.5435	0.7467
SO_RS01710		-1.1475	0.3992	0.6282
SO_RS01715	SO_0358	0.5663	0.7272	0.8977
SO_RS01720	SO_0359	-1.5533	0.0951	0.2732
SO_RS01725	SO_0360	-2.1726	0.1724	0.3802
SO_RS01730	SO_0361	-0.4252	0.8115	0.9607

SO_RS13005	SO_2806	-1.7244	0.3790	0.6078
SO_RS13010	SO_2807	2.8304	0.1031	0.2844
SO_RS13015	SO_2808	1.9392	0.2078	0.4248
SO_RS13020	SO_2809	-6.0577	0.0169	0.1172
SO_RS13025		0.0000	1.0000	1.0000
SO_RS13030	SO_2811	0.6703	0.6716	0.8556
SO_RS13035		-2.1620	0.2776	0.5002
SO_RS13040	SO_2813	0.7835	0.3339	0.5624
SO_RS13045	SO_2814	0.0000	1.0000	1.0000
SO_RS13050	SO_2815	-0.9981	0.3803	0.6093
SO_RS13060		0.0000	1.0000	1.0000
SO_RS13070		-1.7088	0.3162	0.5438
SO_RS13075	SO_2821	2.0909	0.4434	0.6665
SO_RS13080	SO_2822	2.1580	0.2862	0.5093
SO_RS13085	SO_2823	2.0908	0.4286	0.6570
SO_RS13090	SO_2824	2.6515	0.0756	0.2420
SO_RS13095	SO_2827	-1.6671	0.3610	0.5896
SO_RS13100	SO_2829	0.1812	0.8385	0.9781
SO_RS13105	SO_2830	-3.2239	0.0749	0.2414
SO_RS13110	SO_2831	1.5490	0.4731	0.6904
SO_RS13115	SO_2832	2.5128	0.1259	0.3176
SO_RS13120	SO_2833	-0.5132	0.6690	0.8538
SO_RS13125	SO_2834	2.4932	0.0037	0.0581
SO_RS13130	SO_2835	0.0893	0.8940	1.0000
SO_RS13135	SO_2836	1.3401	0.1717	0.3791
SO_RS13140	SO_2838	0.3261	0.8214	0.9660
SO_RS13145	SO_2839	1.4298	0.3780	0.6070
SO_RS13150	SO_2840	0.0000	1.0000	1.0000
SO_RS13155	SO_2841	5.5489	0.0165	0.1161
SO_RS13160	SO_2842	-1.7288	0.2852	0.5089
SO_RS13165	SO_2843	-0.2550	0.8458	0.9837
SO_RS13170	SO_2844	6.9650	0.0146	0.1098
SO_RS13175	SO_2846	6.8856	0.0142	0.1081
SO_RS13180	SO_2847	-0.6825	0.5845	0.7809
SO_RS13185	SO_2848	1.8996	0.2888	0.5114
SO_RS13190	SO_2849	1.8513	0.3762	0.6057
SO_RS13195	SO_2850	0.8015	0.3548	0.5835
SO_RS13225	SO_2851	-0.3410	0.7313	0.8999
SO_RS13230	SO_2852	0.0468	0.9721	1.0000
SO_RS13235	SO_2853	-3.4409	0.0297	0.1468
SO_RS13240	SO_2854	-7.1603	0.0201	0.1233
SO_RS13245	SO_2855	0.0000	1.0000	1.0000
SO_RS13250	SO_2856	9.1168	0.0041	0.0600
SO_RS13255	SO_2857	1.7463	0.0712	0.2359
SO_RS13260	SO_2858	0.0000	1.0000	1.0000
SO_RS13265	SO_2860	0.2289	0.8776	1.0000
SO_RS13270	SO_2861	1.5894	0.3969	0.6259
SO_RS13275	SO_2862	-3.3718	0.0045	0.0630
SO_RS13280	SO_2863	1.4567	0.3373	0.5651
SO_RS13285	SO_2865	8.8098	0.0131	0.1042
SO_RS13290	SO_2866	-1.8296	0.3594	0.5883
SO_RS13295	SO_2867	-0.4057	0.6570	0.8442
SO_RS13300	SO_2868	0.0000	1.0000	1.0000
SO_RS13305	SO_2869	-2.0044	0.0671	0.2296
SO_RS13310	SO_2871	7.1303	0.0707	0.2352
SO_RS13315	SO_2872	4.9267	0.1355	0.3306
SO_RS13330	SO_2876	5.1821	0.0089	0.0873
SO_RS13335	SO_2877	0.6235	0.6937	0.8719
SO_RS13340	SO_2878	-0.3759	0.7835	0.9418
SO_RS13345	SO_2879	1.9537	0.2308	0.4518
SO_RS13350	SO_2880	0.0568	0.9588	1.0000
SO_RS13355	SO_2881	1.5457	0.0157	0.1135
SO_RS13360	SO_2882	2.5868	0.0000	0.0042
SO_RS13365	SO_2883	1.4872	0.0970	0.2754
SO_RS13370	SO_2884	1.3188	0.0689	0.2324
SO_RS13375	SO_2885	1.2022	0.4103	0.6388
SO_RS13380	SO_2886	1.1953	0.1638	0.3686
SO_RS13385	SO_2887	-6.2591	0.0021	0.0460

SO_RS01735	SO_0362	1.5192	0.1001	0.2801
SO_RS01740	SO_0363	-1.7576	0.4004	0.6292
SO_RS01745	SO_0364	-0.8227	0.6161	0.8083
SO_RS01750	SO_0365	-4.3748	0.0562	0.2106
SO_RS01755	SO_0366	8.5676	0.0420	0.1778
SO_RS01760	SO_0367	-4.2619	0.0766	0.2432
SO_RS01765	SO_0368	-2.4544	0.0352	0.1601
SO_RS01770	SO_0369	-1.9688	0.3264	0.5550
SO_RS01775	SO_0370	6.9579	0.0197	0.1217
SO_RS01780	SO_0371	6.6778	0.1063	0.2889
SO_RS01785	SO_0372	3.7946	0.0484	0.1921
SO_RS01790		0.6101	0.4601	0.6800
SO_RS01795	SO_0375	-2.2884	0.0375	0.1675
SO_RS01800		2.1808	0.2091	0.4267
SO_RS01805	SO_0377	-1.8956	0.2890	0.5114
SO_RS01810	SO_0378	-1.4902	0.0592	0.2170
SO_RS01815	SO_0379	-1.9388	0.2541	0.4750
SO_RS01820	SO_0380	-2.7421	0.0101	0.0934
SO_RS01825	SO_0381	3.0985	0.0338	0.1565
SO_RS01830	SO_0382	-2.1953	0.2127	0.4314
SO_RS01835	SO_0383	1.2450	0.5119	0.7234
SO_RS01840	SO_0384	-6.0231	0.0405	0.1753
SO_RS01845	SO_4831	0.0000	1.0000	1.0000
SO_RS01850	SO_0386	0.0000	1.0000	1.0000
SO_RS01855	SO_0387	0.0000	1.0000	1.0000
SO_RS01860	SO_0388	-4.7872	0.0085	0.0868
SO_RS01865	SO_0389	0.9068	0.4674	0.6852
SO_RS01870	SO_0390	0.0000	1.0000	1.0000
SO_RS01875	SO_4762	0.0000	1.0000	1.0000
SO_RS01880	SO_0391	4.7497	0.1520	0.3524
SO_RS01885	SO_0392	-0.9717	0.6480	0.8389
SO_RS01890	SO_0393	-2.0579	0.2513	0.4726
SO_RS01895	SO_0394	-2.4042	0.0069	0.0772
SO_RS01900	SO_0395	1.4127	0.2326	0.4533
SO_RS01905	SO_0396	-2.0762	0.2240	0.4441
SO_RS01910	SO_0397	1.7831	0.0066	0.0765
SO_RS01915	SO_0398	1.2091	0.0171	0.1175
SO_RS01920	SO_0399	-0.3467	0.7204	0.8913
SO_RS01925	SO_0400	3.4259	0.2820	0.5044
SO_RS01930	SO_0401	1.6107	0.1631	0.3679
SO_RS01935	SO_0402	4.8709	0.0087	0.0869
SO_RS01940		-7.6269	0.0177	0.1187
SO_RS01945	SO_0403	-6.4401	0.0129	0.1035
SO_RS01950	SO_0404	-2.1575	0.0047	0.0646
SO_RS01955	SO_0405	-1.5105	0.0051	0.0670
SO_RS01960	SO_0406	1.0412	0.0475	0.1910
SO_RS01965	SO_0407	-0.0760	0.8796	1.0000
SO_RS01970	SO_0408	-0.3057	0.8524	0.9892
SO_RS01975	SO_0409	4.2800	0.0220	0.1279
SO_RS01980	SO_0410	-3.2859	0.1182	0.3073
SO_RS01985	SO_0411	-5.1763	0.0729	0.2402
SO_RS01990	SO_0412	1.6173	0.2379	0.4584
SO_RS01995	SO_0413	0.9424	0.5918	0.7888
SO_RS02000	SO_0414	-0.3774	0.8213	0.9660
SO_RS02005	SO_0415	0.1514	0.8840	1.0000
SO_RS02010	SO_0416	-1.7694	0.0740	0.2405
SO_RS02015	SO_0417	2.6656	0.0032	0.0546
SO_RS02020	SO_0419	9.8314	0.0002	0.0127
SO_RS02025	SO_0421	0.7489	0.5197	0.7304
SO_RS02030	SO_0422	0.3701	0.8240	0.9680
SO_RS02035	SO_0423	0.7239	0.5291	0.7352
SO_RS02040	SO_0424	1.7870	0.0004	0.0188
SO_RS02045	SO_0425	1.6284	0.0058	0.0707
SO_RS02050	SO_0426	1.6487	0.0005	0.0214
SO_RS02055	SO_0427	0.5485	0.3406	0.5692
SO_RS02060	SO_0428	0.3105	0.6046	0.7984
SO_RS02065	SO_0429	2.3852	0.0003	0.0158
SO_RS02070	SO_0430	-1.6732	0.4253	0.6542

SO_RS13390		0.0000	1.0000	1.0000
SO_RS13395	SO_2889	-1.3831	0.1662	0.3720
SO_RS13400	SO_2890	4.4218	0.0112	0.0980
SO_RS13405	SO_2891	0.8244	0.5393	0.7437
SO_RS13410	SO_2893	2.5403	0.0344	0.1580
SO_RS13415	SO_2894	-7.7879	0.0181	0.1196
SO_RS13420	SO_2895	3.2635	0.0292	0.1457
SO_RS13425	SO_2896	2.6809	0.0260	0.1372
SO_RS13430	SO_2897	-0.5803	0.4611	0.6808
SO_RS13435	SO_2898	0.2303	0.8213	0.9660
SO_RS13440	SO_2899	-0.7069	0.4987	0.7124
SO_RS13445	SO_2900	0.3806	0.8501	0.9872
SO_RS13450	SO_2901	1.8309	0.0403	0.1751
SO_RS13455	SO_2902	-3.1829	0.0415	0.1767
SO_RS13460	SO_2903	3.5171	0.0000	0.0028
SO_RS13465	SO_2905	-3.2179	0.0495	0.1947
SO_RS13470	SO_2906	0.5880	0.6904	0.8697
SO_RS13475	SO_2907	3.3283	0.0000	0.0045
SO_RS13480		-7.9577	0.0015	0.0383
SO_RS13490		-5.7087	0.0095	0.0906
SO_RS13495	SO_2911	0.8781	0.1329	0.3263
SO_RS13500	SO_2912	2.2533	0.0001	0.0059
SO_RS13505	SO_2913	1.3564	0.1664	0.3720
SO_RS13510	SO_2914	-2.4839	0.1945	0.4070
SO_RS13515	SO_2915	-0.3434	0.4504	0.6706
SO_RS13520	SO_2916	-0.6282	0.1261	0.3176
SO_RS13525	SO_2917	5.9653	0.0898	0.2640
SO_RS13535	SO_2919	1.2757	0.4385	0.6639
SO_RS13540	SO_2920	2.4951	0.0408	0.1758
SO_RS13545	SO_2921	5.1990	0.0545	0.2074
SO_RS13550	SO_2922	-0.2531	0.5239	0.7321
SO_RS13555	SO_2923	2.7879	0.1416	0.3395
SO_RS13560	SO_2924	-2.7454	0.0009	0.0304
SO_RS13565	SO_2926	-2.1310	0.0383	0.1702
SO_RS13570	SO_2927	-0.2327	0.7933	0.9474
SO_RS13575	SO_2928	-0.6595	0.5902	0.7869
SO_RS13580	SO_2929	1.7141	0.0050	0.0667
SO_RS13585	SO_2930	-0.0431	0.9798	1.0000
SO_RS13590	SO_2931	0.0000	1.0000	1.0000
SO_RS13595	SO_2932	0.5120	0.6157	0.8082
SO_RS13600	SO_2933	-4.9716	0.0102	0.0938
SO_RS13605	SO_2934	4.4258	0.0000	0.0030
SO_RS13610	SO_2935	0.1842	0.8717	1.0000
SO_RS13615	SO_2936	1.9589	0.0418	0.1771
SO_RS13620	SO_2937	-0.2195	0.7325	0.9003
SO_RS13625	SO_2938	1.4615	0.1691	0.3755
SO_RS13630	SO_2939	-0.1358	0.9445	1.0000
SO_RS13635	SO_2940	-1.3436	0.3429	0.5706
SO_RS13640	SO_2941	2.0860	0.5072	0.7192
SO_RS13645	SO_2942	-2.1920	0.3128	0.5400
SO_RS13650	SO_2943	0.0000	1.0000	1.0000
SO_RS13660	SO_2945	-0.9838	0.4742	0.6910
SO_RS13665	SO_2946	-3.4494	0.1168	0.3056
SO_RS13670	SO_2947	0.0000	1.0000	1.0000
SO_RS13675	SO_2948	2.4967	0.2783	0.5009
SO_RS13680	SO_2949	-7.1599	0.0227	0.1291
SO_RS13685	SO_2950	-0.1470	0.9362	1.0000
SO_RS13690	SO_2951	-1.2396	0.0666	0.2293
SO_RS13695	SO_2952	5.9034	0.1169	0.3056
SO_RS13700	SO_2953	-0.0052	0.9973	1.0000
SO_RS13705	SO_2955	0.0000	1.0000	1.0000
SO_RS13710	SO_2956	0.4427	0.7914	0.9462
SO_RS13715	SO_2957	3.8136	0.2488	0.4709
SO_RS13720	SO_2958	4.9193	0.0872	0.2600
SO_RS13725	SO_2959	-3.3365	0.1855	0.3966
SO_RS13730	SO_2960	-4.6748	0.0739	0.2405
SO_RS13735	SO_2961	3.8136	0.2648	0.4860
SO_RS13740	SO_2962	0.0000	1.0000	1.0000

SO_RS02075	SO_0431	3.2130	0.0634	0.2231
SO_RS02080	SO_0432	3.0237	0.0002	0.0136
SO_RS02085	SO_0433	1.3871	0.0153	0.1113
SO_RS02095	SO_0435	0.8351	0.2171	0.4375
SO_RS02105	SO_0437	-0.8018	0.3433	0.5711
SO_RS02110	SO_0438	-1.1048	0.3168	0.5443
SO_RS02115	SO_0439	-3.2351	0.1845	0.3954
SO_RS02120	SO_0440	2.8292	0.1134	0.3007
SO_RS02125	SO_0441	0.0793	0.9089	1.0000
SO_RS02130	SO_0442	-0.5467	0.4277	0.6562
SO_RS02135	SO_0443	-0.1178	0.8728	1.0000
SO_RS02140	SO_0444	1.8544	0.0028	0.0502
SO_RS02145	SO_0445	-1.2918	0.4937	0.7075
SO_RS02150	SO_0447	2.9043	0.3896	0.6185
SO_RS02155	SO_0448	-5.8756	0.0306	0.1481
SO_RS02160	SO_0449	-0.0734	0.9171	1.0000
SO_RS02165	SO_0450	-0.0606	0.9388	1.0000
SO_RS02170	SO_0452	2.0245	0.1066	0.2890
SO_RS02175	SO_0453	-3.2785	0.1452	0.3437
SO_RS02180	SO_0454	4.1130	0.1692	0.3756
SO_RS02185	SO_0455	2.7232	0.0688	0.2324
SO_RS02190	SO_0456	1.9827	0.0918	0.2672
SO_RS02195	SO_0459	4.2025	0.0229	0.1291
SO_RS02200	SO_0461	0.0000	1.0000	1.0000
SO_RS02205	SO_0462	-5.2217	0.0148	0.1100
SO_RS02210	SO_0463	-0.6456	0.7073	0.8818
SO_RS02215	SO_0464	-4.2926	0.0971	0.2754
SO_RS02225	SO_0466	0.2668	0.6181	0.8100
SO_RS02230	SO_4763	-0.3179	0.8754	1.0000
SO_RS02235	SO_0467	0.2836	0.7882	0.9441
SO_RS02240	SO_0468	4.7520	0.0175	0.1182
SO_RS02245	SO_4764	0.0000	1.0000	1.0000
SO_RS02250	SO_0470	0.0000	1.0000	1.0000
SO_RS02255	SO_0471	0.8069	0.4633	0.6823
SO_RS02260	SO_0472	3.0351	0.0071	0.0781
SO_RS02265	SO_0473	-6.7505	0.0448	0.1844
SO_RS02270	SO_0474	-2.6545	0.2086	0.4261
SO_RS02275	SO_0476	-0.7586	0.6583	0.8449
SO_RS02280	SO_0477	3.8034	0.2923	0.5156
SO_RS02285	SO_0478	-2.5862	0.1445	0.3437
SO_RS02290	SO_0479	0.3366	0.7583	0.9218
SO_RS02295	SO_0480	0.0000	1.0000	1.0000
SO_RS02300	SO_0481	-0.1590	0.9305	1.0000
SO_RS02305	SO_0482	9.6898	0.0041	0.0600
SO_RS02310	SO_0483	2.0124	0.2810	0.5035
SO_RS02315	SO_0484	7.2723	0.0458	0.1868
SO_RS02320	SO_0485	4.7497	0.1917	0.4046
SO_RS02325	SO_0486	-0.8081	0.5175	0.7283
SO_RS02330	SO_0487	2.8884	0.0226	0.1291
SO_RS02335	SO_0488	-0.0235	0.9872	1.0000
SO_RS02340	SO_0490	-2.3908	0.0071	0.0781
SO_RS02345	SO_0491	4.5114	0.0004	0.0185
SO_RS02350	SO_0492	1.2146	0.5538	0.7551
SO_RS02355	SO_4765	-1.1420	0.3271	0.5560
SO_RS02360	SO_4766	-0.3201	0.8760	1.0000
SO_RS02365	SO_0494	-3.9602	0.0001	0.0094
SO_RS02370	SO_4767	0.0000	1.0000	1.0000
SO_RS02375	SO_0496	-6.6071	0.0104	0.0944
SO_RS02380	SO_0497	-0.2408	0.8461	0.9837
SO_RS02385	SO_0499	0.0000	1.0000	1.0000
SO_RS02390	SO_0500	10.2418	0.0003	0.0154
SO_RS02395	SO_0501	0.9402	0.3600	0.5888
SO_RS02400	SO_0502	4.1041	0.2119	0.4300
SO_RS02405	SO_0503	1.2364	0.4530	0.6730
SO_RS02410	SO_0504	-0.1889	0.8868	1.0000
SO_RS02415	SO_0505	-2.5356	0.1951	0.4073
SO_RS02420	SO_0506	-0.8960	0.4079	0.6371
SO_RS02425	SO_0508	0.9482	0.2186	0.4385

SO_RS13745	SO_2963	-0.2089	0.8161	0.9637
SO_RS13750	SO_2964	-1.1882	0.3200	0.5482
SO_RS13755	SO_2965	-0.0114	0.9947	1.0000
SO_RS13760	SO_2968	-6.8070	0.0191	0.1206
SO_RS13765	SO_2969	4.3529	0.2016	0.4156
SO_RS13770	SO_2970	-5.8723	0.0329	0.1541
SO_RS13775	SO_2971	-5.0650	0.0586	0.2159
SO_RS13780	SO_2972	5.2028	0.0733	0.2405
SO_RS13785	SO_2973	4.1154	0.1950	0.4073
SO_RS13790	SO_2974	0.0000	1.0000	1.0000
SO_RS13795	SO_4791	-0.8938	0.6594	0.8460
SO_RS13800	SO_2975	2.3080	0.1670	0.3729
SO_RS13805	SO_2976	7.3038	0.0611	0.2202
SO_RS13810	SO_2977	0.0000	1.0000	1.0000
SO_RS13815	SO_2978	-5.8406	0.0025	0.0490
SO_RS13820	SO_2979	-4.6840	0.0662	0.2288
SO_RS13825	SO_2980	-9.1784	0.0179	0.1189
SO_RS13830	SO_2981	5.5332	0.1049	0.2872
SO_RS13835	SO_2982	-4.5395	0.0171	0.1175
SO_RS13840	SO_2983	3.8059	0.1583	0.3610
SO_RS13845	SO_2984	1.8918	0.2805	0.5030
SO_RS13850	SO_2985	6.1791	0.0235	0.1303
SO_RS13855	SO_2986	-1.8286	0.3479	0.5760
SO_RS13860	SO_2987	0.0000	1.0000	1.0000
SO_RS13865	SO_2988	1.4860	0.4191	0.6478
SO_RS13870	SO_2989	0.0000	1.0000	1.0000
SO_RS13875	SO_2990	-2.2183	0.2603	0.4819
SO_RS13880	SO_2991	2.8136	0.2186	0.4385
SO_RS13885	SO_2992	-8.7270	0.0201	0.1233
SO_RS13890	SO_4793	0.0000	1.0000	1.0000
SO_RS13895	SO_2993	-0.0396	0.9804	1.0000
SO_RS13900		6.7943	0.0956	0.2733
SO_RS13905	SO_2997	-1.1864	0.4686	0.6861
SO_RS13910	SO_2998	4.1152	0.2623	0.4836
SO_RS13915	SO_3000	0.4317	0.7395	0.9058
SO_RS13920	SO_3001	7.6173	0.0096	0.0918
SO_RS13925	SO_3002	3.9300	0.0557	0.2100
SO_RS13930	SO_3003	2.0908	0.4606	0.6803
SO_RS13935	SO_3004	1.4497	0.3365	0.5647
SO_RS13940	SO_3005	-6.1845	0.0498	0.1948
SO_RS13945	SO_3006	-1.0688	0.2275	0.4491
SO_RS13950	SO_3008	3.8136	0.2593	0.4813
SO_RS13955	SO_3009	4.9266	0.1716	0.3791
SO_RS13960	SO_3010	0.0000	1.0000	1.0000
SO_RS13965	SO_4795	0.0000	1.0000	1.0000
SO_RS13970	SO_3012	0.0000	1.0000	1.0000
SO_RS13975	SO_3013	-1.5844	0.3314	0.5598
SO_RS13980	SO_3014	-1.2949	0.3656	0.5948
SO_RS13985	SO_3015	-0.8666	0.3630	0.5913
SO_RS13990	SO_3016	-1.4956	0.0755	0.2420
SO_RS13995	SO_3017	3.2209	0.0197	0.1217
SO_RS14000	SO_3019	0.8594	0.5123	0.7239
SO_RS14005	SO_3020	8.4053	0.0049	0.0661
SO_RS14010	SO_3021	-3.2699	0.1158	0.3038
SO_RS14015	SO_3022	1.0515	0.5239	0.7321
SO_RS14020	SO_3023	1.2475	0.4228	0.6515
SO_RS14025	SO_3024	1.7942	0.2708	0.4926
SO_RS14030	SO_3025	1.7619	0.2011	0.4149
SO_RS14035	SO_3027	4.1154	0.1961	0.4080
SO_RS14040	SO_3029	3.8136	0.2296	0.4506
SO_RS14045	SO_3030	-0.3057	0.7855	0.9423
SO_RS14050	SO_3031	6.0344	0.0065	0.0762
SO_RS14055	SO_3032	-0.2993	0.6352	0.8273
SO_RS14060	SO_3033	-0.8016	0.2577	0.4791
SO_RS14065	SO_3034	-0.1940	0.9180	1.0000
SO_RS14070	SO_3035	-0.6445	0.7128	0.8860
SO_RS14075	SO_3036	2.0907	0.4922	0.7063
SO_RS14080	SO_3037	-1.4136	0.2292	0.4506

SO_RS02430	SO_0510	3.0589	0.2201	0.4402
SO_RS02435	SO_0511	-1.3037	0.4798	0.6966
SO_RS02440	SO_0512	-0.4734	0.7855	0.9423
SO_RS02445	SO_0513	5.7346	0.0503	0.1956
SO_RS02450	SO_0514	3.2376	0.1153	0.3030
SO_RS02455	SO_0515	2.8471	0.1527	0.3524
SO_RS02460	SO_0516	-0.5368	0.6877	0.8668
SO_RS02465	SO_0518	0.0000	1.0000	1.0000
SO_RS02470	SO_0519	-5.4708	0.0333	0.1545
SO_RS02475	SO_0520	0.3812	0.6983	0.8755
SO_RS02480	SO_0521	1.3429	0.2802	0.5027
SO_RS02485	SO_0522	-3.7848	0.0963	0.2745
SO_RS02490	SO_0523	6.6155	0.0689	0.2324
SO_RS02495	SO_0524	-3.1400	0.0408	0.1758
SO_RS02500	SO_0525	-0.5643	0.5335	0.7390
SO_RS02505	SO_0526	3.2634	0.0710	0.2357
SO_RS02510	SO_0527	0.6102	0.7428	0.9078
SO_RS02515	SO_0528	0.0714	0.9503	1.0000
SO_RS02520	SO_0529	8.8411	0.0035	0.0568
SO_RS02525	SO_0530	-1.6039	0.3386	0.5666
SO_RS02530	SO_0532	3.5015	0.0409	0.1758
SO_RS02535	SO_0533	4.1032	0.0587	0.2160
SO_RS02540	SO_0534	2.0990	0.1923	0.4047
SO_RS02545	SO_0535	-0.9573	0.5653	0.7649
SO_RS02550	SO_0536	-0.6099	0.7304	0.8999
SO_RS02555	SO_0538	1.1300	0.4895	0.7039
SO_RS02560	SO_0539	0.3466	0.8019	0.9538
SO_RS02565	SO_0540	-0.9240	0.6869	0.8665
SO_RS02570	SO_0541	1.7095	0.0140	0.1073
SO_RS02575	SO_0542	-0.5315	0.5260	0.7331
SO_RS02580	SO_0544	-0.0405	0.9770	1.0000
SO_RS02585	SO_4768	6.7536	0.0319	0.1513
SO_RS02590	SO_0545	-0.2317	0.8010	0.9530
SO_RS02595	SO_0546	-0.6935	0.5395	0.7437
SO_RS02600	SO_0547	-3.9308	0.0097	0.0918
SO_RS02605	SO_0548	-3.8226	0.0257	0.1362
SO_RS02610	SO_0549	7.8035	0.0318	0.1510
SO_RS02615	SO_0550	0.0000	1.0000	1.0000
SO_RS02620	SO_0551	7.1023	0.0126	0.1025
SO_RS02625	SO_0552	-0.5063	0.5317	0.7378
SO_RS02630	SO_0553	0.0000	1.0000	1.0000
SO_RS02635	SO_0554	-0.6757	0.1266	0.3176
SO_RS02640	SO_0555	-3.7716	0.0623	0.2215
SO_RS02645	SO_0556	-1.6759	0.1908	0.4038
SO_RS02650	SO_0557	-1.3532	0.4055	0.6351
SO_RS02655	SO_0558	9.0363	0.0149	0.1102
SO_RS02660	SO_0559	2.8218	0.0910	0.2657
SO_RS02665	SO_0560	-1.9121	0.1031	0.2844
SO_RS02670	SO_0561	-1.0818	0.4946	0.7086
SO_RS02675	SO_0562	2.0859	0.4373	0.6636
SO_RS02685	SO_0564	1.3914	0.1733	0.3813
SO_RS02690	SO_0565	1.5398	0.3301	0.5587
SO_RS02695	SO_0566	-1.6491	0.1911	0.4040
SO_RS02700	SO_0567	-1.3061	0.5132	0.7244
SO_RS02705	SO_0568	-2.1745	0.1637	0.3685
SO_RS02710	SO_0569	-0.2335	0.8797	1.0000
SO_RS02715	SO_0570	-3.4697	0.0409	0.1758
SO_RS02720	SO_0572	-5.1141	0.0044	0.0623
SO_RS02725	SO_0573	-2.5469	0.1332	0.3266
SO_RS02730	SO_0575	1.2959	0.4025	0.6314
SO_RS02735	SO_0576	2.5554	0.0017	0.0424
SO_RS02740	SO_0577	0.2049	0.8150	0.9630
SO_RS02745	SO_0578	-0.0234	0.9676	1.0000
SO_RS02750	SO_0579	-0.8462	0.3164	0.5438
SO_RS02755	SO_0581	0.1015	0.9313	1.0000
SO_RS02760	SO_0582	7.0360	0.0008	0.0277
SO_RS02765	SO_0583	-3.2322	0.2227	0.4427
SO_RS02770	SO_0584	-0.2190	0.7037	0.8796
SO_RS14085	SO_3038	-3.3361	0.2403	0.4612
SO_RS14090	SO_4797	-3.2551	0.1974	0.4102
SO_RS14095	SO_3039	7.0779	0.0676	0.2307
SO_RS14100	SO_3040	-5.5613	0.0138	0.1068
SO_RS14105	SO_3041	0.7559	0.6212	0.8132
SO_RS14110	SO_3042	0.0000	1.0000	1.0000
SO_RS14115	SO_3043	-1.8065	0.2361	0.4569
SO_RS14120	SO_3044	-4.5021	0.0669	0.2293
SO_RS14125	SO_3045	5.0686	0.0683	0.2316
SO_RS14130	SO_3046	-0.0541	0.9703	1.0000
SO_RS14135	SO_3047	4.3381	0.0814	0.2497
SO_RS14140	SO_3048	0.7161	0.6150	0.8079
SO_RS14145	SO_3049	6.9538	0.0392	0.1728
SO_RS14150	SO_3050	0.0000	1.0000	1.0000
SO_RS14155	SO_3051	2.7910	0.0196	0.1217
SO_RS14160	SO_3052	0.9106	0.0592	0.2170
SO_RS14165	SO_3054	-0.1666	0.8258	0.9683
SO_RS14170	SO_3055	2.1375	0.0773	0.2438
SO_RS14175	SO_3056	0.0000	1.0000	1.0000
SO_RS14180	SO_3057	5.1356	0.0130	0.1035
SO_RS14185	SO_3058	11.0572	0.0019	0.0436
SO_RS14190	SO_3059	0.8153	0.5007	0.7139
SO_RS14195	SO_3060	2.9041	0.3134	0.5404
SO_RS14200	SO_3061	1.6923	0.1239	0.3147
SO_RS14205	SO_3062	0.0000	1.0000	1.0000
SO_RS14210	SO_3063	-2.4151	0.2178	0.4380
SO_RS14215	SO_3064	1.0776	0.0337	0.1562
SO_RS14220	SO_3065	5.7228	0.0697	0.2339
SO_RS14225	SO_3066	0.6440	0.4666	0.6847
SO_RS14230	SO_3067	0.0489	0.9554	1.0000
SO_RS14235	SO_3068	0.0016	0.9986	1.0000
SO_RS14240	SO_3069	-0.2353	0.5716	0.7693
SO_RS14245	SO_3070	-0.8812	0.2489	0.4709
SO_RS14250	SO_3071	-0.4074	0.7099	0.8839
SO_RS14255	SO_3072	-0.6734	0.2427	0.4639
SO_RS14260	SO_3073	-1.6331	0.3104	0.5369
SO_RS14265	SO_3075	7.2099	0.0307	0.1484
SO_RS14270	SO_3076	-6.4678	0.0275	0.1418
SO_RS14275	SO_3077	-1.9642	0.1005	0.2809
SO_RS14280	SO_3078	0.0780	0.9719	1.0000
SO_RS14285	SO_3079	-0.1096	0.9013	1.0000
SO_RS14290	SO_3080	2.3526	0.0173	0.1179
SO_RS14295	SO_3081	-2.1670	0.2267	0.4481
SO_RS14300	SO_3082	2.4233	0.0693	0.2331
SO_RS14305	SO_3083	1.4636	0.1848	0.3957
SO_RS14310	SO_3084	-1.0519	0.5886	0.7852
SO_RS14315	SO_3085	2.4660	0.0006	0.0244
SO_RS14320	SO_3088	2.2908	0.0010	0.0322
SO_RS14325	SO_3089	9.4007	0.0000	0.0022
SO_RS14330	SO_3090	1.8165	0.1701	0.3769
SO_RS14335	SO_3091	-1.3850	0.4668	0.6847
SO_RS14340	SO_3092	7.4831	0.0614	0.2202
SO_RS14345	SO_3093	0.0838	0.9643	1.0000
SO_RS14350	SO_3094	-2.1105	0.2624	0.4837
SO_RS14355	SO_3095	-2.4419	0.0182	0.1199
SO_RS14360	SO_3096	-1.4580	0.1624	0.3675
SO_RS14365	SO_3097	2.2105	0.0023	0.0465
SO_RS14370	SO_3098	-1.8337	0.2092	0.4268
SO_RS14375	SO_3099	1.2194	0.1373	0.3327
SO_RS14380	SO_3100	0.0000	1.0000	1.0000
SO_RS14385	SO_3101	0.3097	0.5707	0.7692
SO_RS14390	SO_3102	4.4922	0.0115	0.0986
SO_RS14395	SO_3103	3.0243	0.0003	0.0158
SO_RS14400	SO_3104	2.0861	0.4794	0.6966
SO_RS14405	SO_3105	1.5533	0.2497	0.4709
SO_RS14410	SO_3106	10.1876	0.0020	0.0453
SO_RS14415	SO_3107	-1.5442	0.2435	0.4644
SO_RS14420	SO_3108	5.7258	0.0003	0.0149

SO_RS02775	SO_0585	5.8154	0.0328	0.1538
SO_RS02780	SO_0586	4.1041	0.2799	0.5023
SO_RS02785	SO_0587	3.5227	0.0440	0.1819
SO_RS02790	SO_0588	0.5036	0.6247	0.8166
SO_RS02795	SO_0589	4.3529	0.2096	0.4271
SO_RS02800	SO_0590	2.5892	0.0939	0.2709
SO_RS02805	SO_0591	4.7405	0.0114	0.0981
SO_RS02810	SO_0592	-1.9618	0.0430	0.1792
SO_RS02835	SO_0595	1.7530	0.0090	0.0875
SO_RS02840	SO_0596	8.5466	0.0358	0.1618
SO_RS02845	SO_0598	-0.0375	0.9616	1.0000
SO_RS02850	SO_0599	-0.8371	0.5985	0.7930
SO_RS02855	SO_0600	-3.4712	0.0084	0.0868
SO_RS02860	SO_0601	-2.2740	0.0799	0.2470
SO_RS02865	SO_0602	-1.0211	0.0835	0.2536
SO_RS02870	SO_0603	-2.2318	0.1585	0.3610
SO_RS02875	SO_0604	-1.3787	0.0038	0.0583
SO_RS02880	SO_0605	-1.0582	0.0582	0.2154
SO_RS02885	SO_0606	1.2567	0.1259	0.3176
SO_RS02890	SO_0608	1.1688	0.0727	0.2397
SO_RS02895	SO_0609	2.5772	0.0001	0.0087
SO_RS02900	SO_0610	0.7383	0.1084	0.2907
SO_RS02905	SO_0611	-0.4211	0.4985	0.7124
SO_RS02910	SO_0612	0.4941	0.6015	0.7958
SO_RS02915	SO_0613	8.8628	0.0382	0.1698
SO_RS02920	SO_0614	0.1488	0.8245	0.9680
SO_RS02925	SO_0615	1.6277	0.2368	0.4575
SO_RS02930	SO_0616	7.2879	0.0548	0.2076
SO_RS02935	SO_0617	1.9236	0.0014	0.0369
SO_RS02940	SO_0618	1.6430	0.1058	0.2888
SO_RS02945	SO_0619	0.6128	0.2696	0.4919
SO_RS02950	SO_0620	2.7732	0.0183	0.1201
SO_RS02955	SO_0621	0.7894	0.6127	0.8057
SO_RS02960	SO_0622	-1.6026	0.1182	0.3073
SO_RS02965	SO_0623	0.0000	1.0000	1.0000
SO_RS02970	SO_0624	-0.1467	0.7860	0.9426
SO_RS02975	SO_0625	3.4894	0.0340	0.1569
SO_RS02980	SO_0627	0.7773	0.7192	0.8908
SO_RS02985	SO_0628	-1.8685	0.2088	0.4263
SO_RS02990	SO_0630	5.3174	0.0741	0.2405
SO_RS02995	SO_0632	8.7562	0.0034	0.0564
SO_RS03000	SO_0633	8.6098	0.0021	0.0463
SO_RS03005	SO_0635	2.0861	0.5202	0.7304
SO_RS03010	SO_0636	-2.4831	0.1969	0.4094
SO_RS03015	SO_0637	0.7445	0.6084	0.8020
SO_RS03020	SO_0639	-0.1245	0.9507	1.0000
SO_RS03025	SO_0640	4.1339	0.0153	0.1113
SO_RS03030	SO_0641	-1.0839	0.2844	0.5080
SO_RS03035	SO_4769	0.0000	1.0000	1.0000
SO_RS03040	SO_0643	2.5756	0.2500	0.4712
SO_RS03045	SO_0644	5.2287	0.0301	0.1469
SO_RS03050	SO_0645	0.0000	1.0000	1.0000
SO_RS03055	SO_0646	0.0000	1.0000	1.0000
SO_RS03065	SO_0648	3.8085	0.1788	0.3878
SO_RS03070	SO_0649	0.0000	1.0000	1.0000
SO_RS03080	SO_0650	0.0000	1.0000	1.0000
SO_RS03085	SO_0651	-8.7787	0.0172	0.1179
SO_RS03090	SO_0652	0.0000	1.0000	1.0000
SO_RS03095	SO_0653	-0.6348	0.5665	0.7659
SO_RS03100	SO_0654	0.2693	0.8750	1.0000
SO_RS03105	SO_0655	-1.8345	0.2196	0.4394
SO_RS03115	SO_0658	0.0000	1.0000	1.0000
SO_RS03120	SO_0659	-0.8887	0.7190	0.8908
SO_RS03125	SO_0660	0.0000	1.0000	1.0000
SO_RS03135	SO_0662	0.0000	1.0000	1.0000
SO_RS03140	SO_0663	0.0000	1.0000	1.0000
SO_RS03150	SO_0665	0.0000	1.0000	1.0000
SO_RS03155	SO_0666	1.1605	0.5074	0.7192

SO_RS14425	SO_3109	-0.8473	0.6958	0.8734
SO_RS14430	SO_3110	0.4173	0.4363	0.6630
SO_RS14435	SO_3111	-0.4813	0.4543	0.6744
SO_RS14440	SO_3112	-0.1658	0.9019	1.0000
SO_RS14445	SO_3113	-1.1164	0.1245	0.3151
SO_RS14450	SO_3114	-3.2711	0.0403	0.1751
SO_RS14455	SO_3115	2.0907	0.4834	0.6989
SO_RS14460	SO_3116	7.8036	0.0394	0.1731
SO_RS14465	SO_3117	4.1108	0.1194	0.3091
SO_RS14470	SO_3118	0.6688	0.7073	0.8818
SO_RS14475	SO_3119	1.7517	0.0011	0.0328
SO_RS14480	SO_3120	-1.2486	0.1996	0.4126
SO_RS14485	SO_3121	-2.1158	0.1585	0.3610
SO_RS14490	SO_3122	0.4571	0.5718	0.7693
SO_RS14495	SO_3123	1.1808	0.2245	0.4448
SO_RS14500	SO_3124	2.5482	0.1064	0.2890
SO_RS14505	SO_3125	0.3820	0.6844	0.8647
SO_RS14510	SO_3126	9.0918	0.0038	0.0583
SO_RS14515	SO_3127	9.7413	0.0013	0.0359
SO_RS14520	SO_3128	1.8617	0.0755	0.2420
SO_RS14565	SO_3130	1.9316	0.0888	0.2630
SO_RS14595	SO_3132	0.0000	1.0000	1.0000
SO_RS14600	SO_3134	-0.9301	0.6339	0.8268
SO_RS14605	SO_3135	7.3895	0.0216	0.1275
SO_RS14610	SO_3136	0.2607	0.8865	1.0000
SO_RS14615	SO_3137	-1.9316	0.1411	0.3389
SO_RS14620	SO_3138	-2.0579	0.2913	0.5145
SO_RS14625	SO_3139	1.6400	0.0730	0.2403
SO_RS14630	SO_3140	-3.7630	0.0829	0.2529
SO_RS14635	SO_3141	-0.9472	0.4004	0.6292
SO_RS14640	SO_3142	0.4986	0.5596	0.7609
SO_RS14645	SO_3143	6.9679	0.0361	0.1625
SO_RS14650	SO_3144	0.7487	0.5606	0.7612
SO_RS14655	SO_3145	1.1189	0.3216	0.5501
SO_RS14660	SO_3146	0.5181	0.4525	0.6726
SO_RS14665	SO_3148	1.4556	0.3958	0.6244
SO_RS14670	SO_3149	6.3894	0.0239	0.1306
SO_RS14675	SO_3150	3.5390	0.0586	0.2159
SO_RS14680	SO_3151	-5.3198	0.0180	0.1189
SO_RS14685	SO_3152	2.9043	0.3480	0.5760
SO_RS14695	SO_3154	0.0890	0.8532	0.9893
SO_RS14700	SO_3155	-1.0796	0.3472	0.5754
SO_RS14705	SO_4798	1.5633	0.0647	0.2259
SO_RS14710	SO_3157	-0.4123	0.7952	0.9482
SO_RS14715	SO_3158	2.3016	0.1698	0.3768
SO_RS14720	SO_3159	3.2164	0.0019	0.0436
SO_RS14725	SO_3160	-5.6085	0.0394	0.1731
SO_RS14730	SO_3161	0.0000	1.0000	1.0000
SO_RS14735	SO_3162	2.6120	0.0293	0.1457
SO_RS14740	SO_3163	8.7482	0.0018	0.0435
SO_RS14745	SO_3164	0.3910	0.8103	0.9601
SO_RS14750	SO_3165	4.1040	0.1908	0.4038
SO_RS14755	SO_3166	-7.2367	0.0219	0.1279
SO_RS14760	SO_3167	-1.3958	0.2889	0.5114
SO_RS14765	SO_3168	0.6637	0.4433	0.6665
SO_RS14770	SO_3169	1.0144	0.5516	0.7528
SO_RS14775	SO_3170	0.2242	0.7338	0.9012
SO_RS14780	SO_3171	0.9216	0.0629	0.2225
SO_RS14785	SO_3172	2.1885	0.1884	0.4008
SO_RS14790	SO_3173	0.5346	0.3729	0.6025
SO_RS14795	SO_3174	0.2855	0.8053	0.9563
SO_RS14800	SO_3175	1.2078	0.1266	0.3176
SO_RS14805	SO_3176	0.0217	0.9808	1.0000
SO_RS14810	SO_3177	2.3508	0.1160	0.3044
SO_RS14815	SO_3178	5.8078	0.0029	0.0513
SO_RS14820	SO_3179	1.7918	0.0674	0.2302
SO_RS14825	SO_4799	0.9777	0.5828	0.7791
SO_RS14830	SO_3180	0.1403	0.8672	0.9998

SO_RS03160	SO_0667	3.8111	0.2557	0.4770
SO_RS03165	SO_0668	-9.1398	0.0120	0.0991
SO_RS03175	SO_0669	6.6624	0.0710	0.2357
SO_RS03180	SO_0670	-5.3685	0.0387	0.1712
SO_RS03185	SO_0671	-2.7469	0.1321	0.3257
SO_RS03190	SO_0672	0.0895	0.9401	1.0000
SO_RS03195	SO_0673	8.3883	0.0022	0.0463
SO_RS03200	SO_0674	-2.3772	0.2269	0.4483
SO_RS03205	SO_0675	1.9952	0.2687	0.4910
SO_RS03210	SO_0676	0.4054	0.8467	0.9841
SO_RS03215	SO_0677	2.9733	0.2313	0.4523
SO_RS03220	SO_0678	0.0000	1.0000	1.0000
SO_RS03225	SO_0679	-2.3968	0.2507	0.4717
SO_RS03230	SO_0680	5.7228	0.0764	0.2429
SO_RS03235	SO_0681	0.0000	1.0000	1.0000
SO_RS03240	SO_0682	-3.7816	0.1135	0.3008
SO_RS03245		6.7077	0.0828	0.2529
SO_RS03255		0.0000	1.0000	1.0000
SO_RS03260	SO_0687	0.0000	1.0000	1.0000
SO_RS03265	SO_0688	0.3241	0.8137	0.9622
SO_RS03275	SO_0690	1.7039	0.3314	0.5598
SO_RS03280	SO_0691	-0.1005	0.9154	1.0000
SO_RS03285	SO_0693	0.6433	0.4584	0.6787
SO_RS03290	SO_0694	2.4477	0.0069	0.0772
SO_RS03295	SO_0695	5.4295	0.0067	0.0771
SO_RS03300	SO_0696	4.4893	0.0097	0.0918
SO_RS03305	SO_0697	1.1613	0.4960	0.7104
SO_RS03310	SO_0698	-0.3421	0.6798	0.8617
SO_RS03315	SO_0699	-2.3464	0.1032	0.2844
SO_RS03320	SO_0701	-0.7366	0.6856	0.8654
SO_RS03325	SO_0702	8.4256	0.0408	0.1758
SO_RS03330	SO_0703	0.4995	0.7037	0.8796
SO_RS03335	SO_0704	2.7119	0.0000	0.0022
SO_RS03340	SO_0705	0.0000	1.0000	1.0000
SO_RS03345	SO_0706	1.1624	0.3219	0.5502
SO_RS03350		2.0918	0.3483	0.5760
SO_RS03355	SO_0708	-0.1630	0.9238	1.0000
SO_RS03360		-0.0079	0.9959	1.0000
SO_RS03365	SO_0710	-1.7414	0.1785	0.3876
SO_RS03370		2.7010	0.1951	0.4073
SO_RS03380	SO_0714	0.0000	1.0000	1.0000
SO_RS03385	SO_0715	5.4779	0.0132	0.1043
SO_RS03390	SO_0716	-9.0294	0.0196	0.1217
SO_RS03395	SO_0717	0.0000	1.0000	1.0000
SO_RS03400	SO_0718	-0.5046	0.7862	0.9426
SO_RS03405	SO_0719	-1.4224	0.1145	0.3021
SO_RS03410	SO_0720	8.7244	0.0278	0.1427
SO_RS03415	SO_0721	0.0000	1.0000	1.0000
SO_RS03420	SO_0725	-2.2378	0.1504	0.3507
SO_RS03425	SO_0726	2.3848	0.1505	0.3507
SO_RS03430	SO_0727	4.1153	0.1951	0.4073
SO_RS03435	SO_0728	9.3845	0.0315	0.1500
SO_RS03440	SO_0730	-1.0743	0.3987	0.6277
SO_RS03450	SO_0732	3.4321	0.2537	0.4748
SO_RS03455	SO_0733	5.8172	0.0331	0.1541
SO_RS03460	SO_0734	-8.4409	0.0137	0.1061
SO_RS03465	SO_0735	1.1234	0.5096	0.7212
SO_RS03470	SO_0736	7.4042	0.0212	0.1268
SO_RS03475	SO_0737	9.1203	0.0023	0.0465
SO_RS03480	SO_0738	0.0000	1.0000	1.0000
SO_RS03485	SO_0739	3.8136	0.2358	0.4566
SO_RS03490	SO_0740	0.1181	0.7916	0.9462
SO_RS03495	SO_0741	2.9989	0.0087	0.0869
SO_RS03500	SO_0742	-1.7720	0.1235	0.3142
SO_RS03505	SO_0743	-2.2018	0.2738	0.4961
SO_RS03510	SO_0744	0.4836	0.6569	0.8442
SO_RS03515	SO_0745	-2.0127	0.3794	0.6080
SO_RS03520	SO_0746	0.9509	0.3196	0.5478

SO_RS14835	SO_3181	0.1493	0.8661	0.9992
SO_RS14840	SO_3182	-0.4066	0.8355	0.9765
SO_RS14845	SO_3183	1.1755	0.2264	0.4478
SO_RS14850	SO_3184	0.1224	0.8797	1.0000
SO_RS14855	SO_3185	0.5335	0.5594	0.7608
SO_RS14860	SO_3186	0.3586	0.7327	0.9003
SO_RS14870	SO_3188	0.0366	0.9537	1.0000
SO_RS14875	SO_3189	-2.3350	0.0351	0.1594
SO_RS14880	SO_3190	1.2275	0.1748	0.3828
SO_RS14885	SO_3191	-0.0292	0.9789	1.0000
SO_RS14890	SO_3192	0.9455	0.5341	0.7395
SO_RS14895	SO_3193	-1.0262	0.0906	0.2653
SO_RS14900	SO_3194	-1.6911	0.3681	0.5975
SO_RS14905	SO_3195	0.0832	0.8904	1.0000
SO_RS14910	SO_3196	-2.0338	0.1222	0.3132
SO_RS14915	SO_3197	-1.6374	0.0568	0.2121
SO_RS14920	SO_3198	0.6462	0.5356	0.7405
SO_RS14925	SO_3199	4.7624	0.1471	0.3461
SO_RS14930	SO_3200	-0.0190	0.9864	1.0000
SO_RS14935	SO_3202	3.3654	0.0026	0.0494
SO_RS14940	SO_3203	1.3074	0.2745	0.4966
SO_RS14945	SO_3204	-0.0983	0.9161	1.0000
SO_RS14950	SO_3205	-0.3060	0.7676	0.9287
SO_RS14955	SO_3206	-0.5222	0.4337	0.6602
SO_RS14960	SO_3207	-0.2871	0.4047	0.6344
SO_RS14965	SO_3208	0.4442	0.4043	0.6340
SO_RS14970	SO_3209	-0.1834	0.8271	0.9688
SO_RS14975	SO_3210	0.3458	0.5463	0.7486
SO_RS14980	SO_3211	1.3617	0.0190	0.1205
SO_RS14985	SO_3212	1.6434	0.0028	0.0502
SO_RS14990	SO_3213	2.0390	0.0004	0.0186
SO_RS14995	SO_3215	3.6801	0.0333	0.1545
SO_RS15000	SO_3216	-1.7702	0.3611	0.5896
SO_RS15005	SO_3217	-8.7708	0.0027	0.0502
SO_RS15010	SO_3218	-2.9453	0.1408	0.3384
SO_RS15015	SO_3219	8.2586	0.0085	0.0868
SO_RS15020	SO_3220	4.4123	0.0283	0.1436
SO_RS15025	SO_3221	-0.1334	0.8263	0.9683
SO_RS15030	SO_3222	1.2139	0.2786	0.5010
SO_RS15035	SO_3223	3.1243	0.0001	0.0087
SO_RS15040	SO_3224	1.5765	0.4313	0.6591
SO_RS15045	SO_3225	0.5985	0.5963	0.7917
SO_RS15050	SO_3226	2.5559	0.0789	0.2453
SO_RS15055	SO_3227	1.3304	0.0699	0.2342
SO_RS15060	SO_3228	3.6006	0.0038	0.0583
SO_RS15065	SO_3229	9.5060	0.0019	0.0439
SO_RS15070	SO_3230	3.3268	0.0000	0.0045
SO_RS15075	SO_3231	1.9726	0.0013	0.0359
SO_RS15080	SO_3232	1.4168	0.0300	0.1469
SO_RS15085	SO_3233	1.8252	0.0044	0.0621
SO_RS15090	SO_3234	1.5315	0.2340	0.4544
SO_RS15095	SO_3235	1.6823	0.0006	0.0236
SO_RS15100	SO_3236	2.7438	0.0012	0.0341
SO_RS15105	SO_3237	2.7984	0.0000	0.0034
SO_RS15110	SO_3238	4.2672	0.0000	0.0012
SO_RS15115	SO_3239	3.9754	0.0025	0.0490
SO_RS15120	SO_3240	2.7984	0.0001	0.0088
SO_RS15125	SO_3241	2.5416	0.0609	0.2197
SO_RS15130	SO_3242	5.2461	0.0006	0.0238
SO_RS15135	SO_3243	5.3786	0.0040	0.0596
SO_RS15140	SO_3244	2.8877	0.0001	0.0096
SO_RS15145	SO_3245	5.4719	0.0000	0.0034
SO_RS15150	SO_3247	4.8755	0.0000	0.0018
SO_RS15155	SO_3248	4.5422	0.0021	0.0459
SO_RS15160	SO_3249	3.4646	0.0014	0.0370
SO_RS15165	SO_3250	8.5936	0.0000	0.0017
SO_RS15170	SO_3251	1.5091	0.0636	0.2232
SO_RS15175	SO_3252	2.6099	0.0054	0.0682

SO_RS03525	SO_0747	0.6850	0.4921	0.7063
SO_RS03530	SO_0748	0.4608	0.2981	0.5218
SO_RS03535	SO_0749	-3.3387	0.1456	0.3438
SO_RS03540	SO_0750	5.0850	0.0088	0.0869
SO_RS03545	SO_0752	-2.0420	0.1842	0.3948
SO_RS03550	SO_0753	1.0075	0.5626	0.7621
SO_RS03555	SO_0754	0.4464	0.7979	0.9503
SO_RS03560	SO_0756	1.8403	0.0402	0.1750
SO_RS03565	SO_0757	-2.1805	0.3551	0.5837
SO_RS03570	SO_0758	-1.4123	0.3338	0.5624
SO_RS03575	SO_0760	-1.6404	0.3734	0.6029
SO_RS03580	SO_0761	5.0605	0.1452	0.3437
SO_RS03585	SO_0762	2.1885	0.2256	0.4467
SO_RS03590	SO_0764	-5.1556	0.0017	0.0422
SO_RS03595	SO_0765	4.5772	0.1675	0.3735
SO_RS03600	SO_0766	-1.1669	0.5660	0.7654
SO_RS03605	SO_4771	-1.7505	0.3693	0.5985
SO_RS03610	SO_0767	-3.3152	0.1553	0.3560
SO_RS03615	SO_0768	3.7097	0.0600	0.2185
SO_RS03620	SO_0769	0.9811	0.5601	0.7611
SO_RS03625	SO_0770	2.4695	0.0000	0.0052
SO_RS03630	SO_0771	-0.1943	0.8205	0.9660
SO_RS03635	SO_0772	2.4940	0.1675	0.3735
SO_RS03640	SO_0773	-4.1271	0.0627	0.2224
SO_RS03645	SO_0774	-2.1645	0.1420	0.3398
SO_RS03650	SO_m002	-7.0693	0.0000	0.0012
SO_RS03655	SO_0775	1.2516	0.4622	0.6818
SO_RS03660	SO_0776	5.9178	0.0013	0.0358
SO_RS03665	SO_0777	-3.9978	0.0175	0.1182
SO_RS03670	SO_0778	3.1699	0.0683	0.2316
SO_RS03675	SO_0779	1.5933	0.1244	0.3151
SO_RS03680	SO_0780	0.3653	0.5332	0.7388
SO_RS03685	SO_0781	0.3546	0.3591	0.5882
SO_RS03690	SO_0782	1.9217	0.3994	0.6283
SO_RS03695	SO_0783	-3.0576	0.0027	0.0502
SO_RS03700	SO_0784	3.4228	0.2568	0.4777
SO_RS03710	SO_0786	0.0000	1.0000	1.0000
SO_RS03715	SO_0787	2.9043	0.3872	0.6162
SO_RS03720	SO_0788	0.0000	1.0000	1.0000
SO_RS03725	SO_0789	0.0130	0.9928	1.0000
SO_RS03735	SO_0790	-4.6752	0.0659	0.2280
SO_RS03740	SO_0792	0.0000	1.0000	1.0000
SO_RS03745		0.0000	1.0000	1.0000
SO_RS03750	SO_0795	2.8511	0.0128	0.1035
SO_RS03755	SO_0796	5.9685	0.0340	0.1569
SO_RS03760	SO_0797	-5.6662	0.0344	0.1580
SO_RS03765	SO_0798	-1.9065	0.0957	0.2733
SO_RS03770	SO_0799	1.5285	0.4007	0.6293
SO_RS03775	SO_0801	0.3305	0.8401	0.9788
SO_RS03780	SO_0802	-2.8421	0.1831	0.3931
SO_RS03785	SO_0804	4.5719	0.0794	0.2465
SO_RS03790	SO_0805	2.4967	0.0502	0.1953
SO_RS03795	SO_0806	4.1145	0.0257	0.1362
SO_RS03800	SO_0807	-4.0381	0.0210	0.1268
SO_RS03805	SO_0808	-2.0013	0.0933	0.2701
SO_RS03810	SO_0809	1.6644	0.2474	0.4699
SO_RS03815	SO_0810	2.9042	0.3063	0.5320
SO_RS03820	SO_0811	3.2412	0.0515	0.1980
SO_RS03825	SO_0812	-2.1433	0.2383	0.4588
SO_RS03830	SO_0813	-0.8522	0.3118	0.5389
SO_RS03835	SO_0815	1.1301	0.1913	0.4041
SO_RS03840	SO_0816	0.1083	0.9460	1.0000
SO_RS03845	SO_0817	-9.5718	0.0040	0.0600
SO_RS03850	SO_0818	-1.1576	0.3903	0.6194
SO_RS03855	SO_0819	-2.8936	0.1015	0.2828
SO_RS03860	SO_0820	0.3625	0.7625	0.9256
SO_RS03865	SO_0821	-1.5846	0.3589	0.5882
SO_RS03870	SO_0822	1.0104	0.5837	0.7801

SO_RS15180	SO_3253	-3.9018	0.0177	0.1187
SO_RS15185	SO_3254	-0.8630	0.3422	0.5704
SO_RS15190	SO_3255	1.4541	0.2856	0.5091
SO_RS15195	SO_3256	1.6893	0.0259	0.1369
SO_RS15200	SO_3257	1.6595	0.0056	0.0697
SO_RS15205	SO_3258	4.6743	0.0096	0.0918
SO_RS15215	SO_3259	0.6628	0.4248	0.6536
SO_RS15220	SO_3260	2.0860	0.4416	0.6661
SO_RS15225	SO_3261	0.0261	0.9784	1.0000
SO_RS15230	SO_3262	2.0152	0.0066	0.0765
SO_RS15235	SO_3263	0.3383	0.7177	0.8903
SO_RS15240	SO_3264	1.8176	0.0302	0.1471
SO_RS15245	SO_3265	3.4121	0.0027	0.0502
SO_RS15250	SO_3266	2.2714	0.0680	0.2315
SO_RS15255	SO_3267	1.0232	0.1264	0.3176
SO_RS15260	SO_3268	-0.2832	0.7621	0.9254
SO_RS15265	SO_3269	-0.4142	0.6095	0.8031
SO_RS15270	SO_3270	0.9683	0.4280	0.6565
SO_RS15275	SO_3271	2.7682	0.0051	0.0670
SO_RS15280	SO_3272	8.2010	0.0066	0.0765
SO_RS15285	SO_3273	2.7631	0.0133	0.1047
SO_RS15290	SO_3274	2.0229	0.2396	0.4605
SO_RS15295	SO_3275	3.1469	0.0667	0.2293
SO_RS15300	SO_3276	-1.9906	0.0236	0.1303
SO_RS15305	SO_3277	4.5964	0.0069	0.0772
SO_RS15310	SO_3278	-7.2366	0.0238	0.1304
SO_RS15315	SO_3279	-2.3444	0.2132	0.4316
SO_RS15320	SO_3280	0.0000	1.0000	1.0000
SO_RS15325	SO_3282	0.0627	0.9419	1.0000
SO_RS15330	SO_3284	0.0000	1.0000	1.0000
SO_RS15335	SO_3285	4.9171	0.0115	0.0986
SO_RS15340	SO_3286	1.4711	0.0017	0.0422
SO_RS15345	SO_3287	0.4341	0.2262	0.4476
SO_RS15350	SO_3288	-0.8641	0.6046	0.7984
SO_RS15355	SO_3291	-4.1005	0.0998	0.2800
SO_RS15360	SO_3292	-0.7148	0.2745	0.4966
SO_RS15365	SO_3293	1.6085	0.2499	0.4710
SO_RS15370	SO_3294	1.4312	0.4424	0.6665
SO_RS15375	SO_3296	-10.3252	0.0193	0.1210
SO_RS15380	SO_3297	-9.6811	0.0238	0.1305
SO_RS15385	SO_3298	-2.8452	0.0736	0.2405
SO_RS15390	SO_3299	0.6587	0.4495	0.6703
SO_RS15395	SO_3300	0.0000	1.0000	1.0000
SO_RS15400	SO_3301	0.3270	0.8654	0.9990
SO_RS15410	SO_3303	-6.5873	0.0241	0.1310
SO_RS15415	SO_3305	0.0000	1.0000	1.0000
SO_RS15420	SO_3306	1.8774	0.1995	0.4126
SO_RS15425	SO_3308	0.5327	0.5926	0.7890
SO_RS15430	SO_3309	-1.2004	0.0915	0.2666
SO_RS15435	SO_3310	-2.7938	0.0141	0.1076
SO_RS15440	SO_3311	-1.1972	0.1483	0.3473
SO_RS15445	SO_3312	-0.0478	0.9682	1.0000
SO_RS15450	SO_3313	-0.9188	0.2401	0.4611
SO_RS15455	SO_3314	-0.7731	0.3368	0.5647
SO_RS15460	SO_3315	-0.2514	0.7941	0.9474
SO_RS15465	SO_3316	2.0793	0.2209	0.4408
SO_RS15470	SO_3317	-1.3382	0.3818	0.6106
SO_RS15475	SO_3318	7.0406	0.0204	0.1248
SO_RS15480	SO_3319	5.4288	0.1511	0.3513
SO_RS15485		3.4319	0.2682	0.4903
SO_RS15495		0.0000	1.0000	1.0000
SO_RS15500	SO_3323	2.8506	0.1332	0.3266
SO_RS15505	SO_3324	1.8692	0.2622	0.4836
SO_RS15510	SO_3325	-0.5313	0.5421	0.7456
SO_RS15515	SO_3326	0.0000	1.0000	1.0000
SO_RS15520	SO_3328	3.4289	0.2046	0.4202
SO_RS15525	SO_3331	0.0000	1.0000	1.0000
SO_RS15530	SO_3332	0.0000	1.0000	1.0000

SO_RS03875	SO_0823	-0.1161	0.9427	1.0000
SO_RS03880	SO_0824	-5.0008	0.0595	0.2172
SO_RS03885	SO_0826	1.9046	0.1164	0.3048
SO_RS03890	SO_0827	-1.8843	0.2293	0.4506
SO_RS03895	SO_0828	0.9007	0.5689	0.7675
SO_RS03900	SO_0830	0.8599	0.4760	0.6932
SO_RS03905	SO_0831	0.9853	0.5286	0.7348
SO_RS03910	SO_0832	10.0444	0.0005	0.0218
SO_RS03915	SO_0833	0.4483	0.7827	0.9418
SO_RS03920	SO_0834	2.9041	0.3143	0.5416
SO_RS03925	SO_0835	-2.1809	0.0431	0.1792
SO_RS03930	SO_0837	0.9963	0.3879	0.6169
SO_RS03935	SO_0839	-1.6651	0.3695	0.5985
SO_RS03940	SO_0840	1.2167	0.0038	0.0583
SO_RS03945	SO_0841	0.1919	0.9250	1.0000
SO_RS03950	SO_0842	0.1889	0.5639	0.7636
SO_RS03955	SO_0843	-0.0132	0.9941	1.0000
SO_RS03960	SO_0845	6.0381	0.0873	0.2600
SO_RS03965	SO_0846	6.8389	0.0189	0.1205
SO_RS03970	SO_0847	1.6802	0.2434	0.4644
SO_RS03975	SO_0848	0.5254	0.5376	0.7425
SO_RS03980	SO_0849	3.8033	0.2357	0.4566
SO_RS03985	SO_0850	-3.3319	0.1077	0.2903
SO_RS03990	SO_0851	-6.8060	0.0219	0.1279
SO_RS03995	SO_0852	-3.6575	0.1250	0.3159
SO_RS04000	SO_0853	-2.0168	0.3605	0.5894
SO_RS04005	SO_0854	0.0000	1.0000	1.0000
SO_RS04010	SO_0855	6.7743	0.0013	0.0357
SO_RS04015	SO_0856	1.7636	0.1900	0.4026
SO_RS04020	SO_0857	0.6462	0.2636	0.4849
SO_RS04025	SO_0858	1.5425	0.0520	0.1996
SO_RS04030	SO_0859	1.0103	0.0165	0.1163
SO_RS04035	SO_0860	-1.8949	0.0429	0.1792
SO_RS04040	SO_0861	2.3137	0.0104	0.0944
SO_RS04045	SO_0862	2.8249	0.0000	0.0045
SO_RS04050	SO_0863	1.2798	0.3066	0.5322
SO_RS04055	SO_0864	-1.0761	0.4485	0.6701
SO_RS04060	SO_0865	0.6514	0.5589	0.7605
SO_RS04065	SO_0866	0.0000	1.0000	1.0000
SO_RS04070	SO_0867	-0.8376	0.5457	0.7486
SO_RS04075	SO_0868	5.6306	0.1077	0.2903
SO_RS04080	SO_0869	-1.6592	0.1910	0.4039
SO_RS04085	SO_0870	3.3446	0.0482	0.1919
SO_RS04090	SO_0871	-2.5866	0.1924	0.4047
SO_RS04095	SO_0872	-2.0535	0.0360	0.1622
SO_RS04100	SO_0873	3.2009	0.0758	0.2422
SO_RS04105	SO_0874	0.2674	0.8558	0.9913
SO_RS04110	SO_0875	5.1940	0.1231	0.3141
SO_RS04115	SO_0876	0.6556	0.2845	0.5080
SO_RS04120	SO_0877	4.8024	0.0048	0.0655
SO_RS04125	SO_0878	2.8808	0.0218	0.1279
SO_RS04130	SO_0879	0.0000	1.0000	1.0000
SO_RS04135	SO_0880	3.4321	0.2585	0.4802
SO_RS04140	SO_0881	0.7912	0.5863	0.7828
SO_RS04145	SO_0882	-1.8373	0.0927	0.2692
SO_RS04150	SO_0883	5.9034	0.0897	0.2640
SO_RS04155	SO_0884	2.7605	0.0975	0.2762
SO_RS04160	SO_0885	-2.2464	0.0264	0.1382
SO_RS04165	SO_0886	-3.3371	0.1535	0.3532
SO_RS04170	SO_0887	0.8154	0.4190	0.6478
SO_RS04175	SO_0888	-0.8810	0.2495	0.4709
SO_RS04180		-1.9620	0.2211	0.4408
SO_RS04190		0.0000	1.0000	1.0000
SO_RS04195	SO_0895	2.4951	0.0060	0.0725
SO_RS04200	SO_0897	-1.1137	0.4211	0.6496
SO_RS04205	SO_0898	4.7497	0.1749	0.3828
SO_RS04210	SO_0899	1.5217	0.3750	0.6045
SO_RS04215	SO_0900	1.6482	0.0909	0.2657

SO_RS15535	SO_3333	-0.5144	0.5528	0.7543
SO_RS15540	SO_3334	3.4227	0.2566	0.4777
SO_RS15545	SO_3335	7.8697	0.0429	0.1792
SO_RS15550	SO_3336	-1.9340	0.1356	0.3306
SO_RS15555	SO_3337	-3.0887	0.0462	0.1877
SO_RS15560	SO_3338	3.9743	0.0257	0.1362
SO_RS15565	SO_3339	-0.3528	0.7723	0.9324
SO_RS15570	SO_3340	1.5666	0.0913	0.2662
SO_RS15575	SO_3341	4.4055	0.0011	0.0328
SO_RS15580	SO_3342	-4.0081	0.1021	0.2832
SO_RS15585	SO_3343	2.8072	0.0004	0.0186
SO_RS15590	SO_3344	-0.9766	0.5454	0.7484
SO_RS15595	SO_3345	3.1806	0.0838	0.2543
SO_RS15600	SO_3346	0.8630	0.5955	0.7912
SO_RS15605	SO_3347	0.0000	1.0000	1.0000
SO_RS15610	SO_3348	-0.6785	0.2095	0.4271
SO_RS15615	SO_3349	-1.9525	0.0003	0.0158
SO_RS15620	SO_3350	2.8538	0.0022	0.0463
SO_RS15625	SO_3351	1.5037	0.1289	0.3203
SO_RS15630	SO_3352	0.0774	0.9613	1.0000
SO_RS15635	SO_3354	0.8178	0.2676	0.4894
SO_RS15640	SO_3355	-0.9456	0.4386	0.6639
SO_RS15645	SO_3356	0.0000	1.0000	1.0000
SO_RS15650	SO_3357	0.0000	1.0000	1.0000
SO_RS15655	SO_3358	-0.0514	0.9644	1.0000
SO_RS15660	SO_3359	8.8475	0.0019	0.0443
SO_RS15665	SO_3360	-2.5634	0.1957	0.4075
SO_RS15670	SO_3361	-4.3779	0.0128	0.1035
SO_RS15675	SO_3363	-0.6297	0.2859	0.5091
SO_RS15680	SO_3364	-0.4799	0.5993	0.7932
SO_RS15685	SO_3365	6.7233	0.0903	0.2643
SO_RS15690	SO_3366	-1.9940	0.2234	0.4436
SO_RS15695	SO_3367	1.9869	0.0885	0.2625
SO_RS15700	SO_3368	2.0859	0.4431	0.6665
SO_RS15705	SO_3369	0.0000	1.0000	1.0000
SO_RS15710	SO_3370	2.2621	0.1403	0.3378
SO_RS15715	SO_3371	-0.1761	0.9366	1.0000
SO_RS15740	SO_3373	0.0000	1.0000	1.0000
SO_RS15745	SO_3374	0.5071	0.5027	0.7151
SO_RS15750	SO_4801	0.0000	1.0000	1.0000
SO_RS15755	SO_3376	-1.8365	0.2384	0.4589
SO_RS15760	SO_3377	-2.0696	0.1195	0.3092
SO_RS15765	SO_3378	-3.6759	0.0942	0.2714
SO_RS15770	SO_3379	3.9312	0.0007	0.0256
SO_RS15775	SO_3380	-3.1597	0.1956	0.4075
SO_RS15780	SO_3381	-0.4389	0.5590	0.7605
SO_RS15785	SO_3382	1.0066	0.5367	0.7415
SO_RS15790	SO_3383	-1.9046	0.0310	0.1492
SO_RS15795	SO_3384	-2.6293	0.1370	0.3321
SO_RS15800	SO_3385	-1.7588	0.0145	0.1094
SO_RS15805	SO_3386	-2.3952	0.0004	0.0186
SO_RS15810	SO_3387	3.5388	0.0753	0.2420
SO_RS15815	SO_3388	-0.4280	0.5985	0.7930
SO_RS15820	SO_3389	2.0595	0.1679	0.3740
SO_RS15825	SO_3391	-0.6215	0.4804	0.6970
SO_RS15830	SO_3392	2.8126	0.0001	0.0079
SO_RS15835	SO_3393	0.3908	0.7404	0.9061
SO_RS15840	SO_3395	3.4455	0.1148	0.3024
SO_RS15845		3.8894	0.0487	0.1928
SO_RS15855	SO_3399	9.3303	0.0002	0.0112
SO_RS15860	SO_3401	2.8299	0.0498	0.1948
SO_RS15870	SO_3403	0.5224	0.2580	0.4794
SO_RS15875	SO_3404	3.5219	0.0592	0.2170
SO_RS15880	SO_3406	7.4318	0.0155	0.1122
SO_RS15885	SO_3407	-2.7203	0.0427	0.1786
SO_RS15890	SO_3408	0.0000	1.0000	1.0000
SO_RS15895	SO_3409	1.4324	0.3486	0.5764
SO_RS15900	SO_3410	0.3868	0.5009	0.7140

SO_RS04220	SO_0902	-0.0649	0.9306	1.0000
SO_RS04225	SO_0903	-0.4914	0.6936	0.8719
SO_RS04230	SO_0904	-0.2480	0.7517	0.9163
SO_RS04235	SO_0905	1.3590	0.3952	0.6240
SO_RS04240	SO_0906	2.9667	0.0862	0.2583
SO_RS04245	SO_0907	1.6665	0.0808	0.2487
SO_RS04250	SO_0908	0.0000	1.0000	1.0000
SO_RS04255	SO_0909	6.2454	0.0413	0.1766
SO_RS04260	SO_0910	0.1127	0.8749	1.0000
SO_RS04270	SO_0911	0.0000	1.0000	1.0000
SO_RS04275	SO_0912	0.9026	0.6608	0.8463
SO_RS04280	SO_0913	-1.3483	0.4915	0.7058
SO_RS04285	SO_0914	-1.7836	0.1728	0.3802
SO_RS04290	SO_0915	-2.9141	0.1776	0.3864
SO_RS04295	SO_0916	3.5013	0.0855	0.2571
SO_RS04300	SO_0917	6.7943	0.0713	0.2360
SO_RS04305	SO_0918	0.3892	0.4626	0.6818
SO_RS04310	SO_0919	-0.1028	0.9488	1.0000
SO_RS04315	SO_0920	3.8085	0.2313	0.4523
SO_RS04320	SO_0921	-0.1016	0.9483	1.0000
SO_RS04325	SO_0922	-0.5080	0.6569	0.8442
SO_RS04330	SO_0923	1.6097	0.3059	0.5318
SO_RS04335	SO_0924	0.0953	0.9651	1.0000
SO_RS04340	SO_0925	-0.3959	0.7578	0.9214
SO_RS04345	SO_0926	-2.8299	0.1046	0.2869
SO_RS04350	SO_0927	0.0000	1.0000	1.0000
SO_RS04355	SO_0928	-1.4668	0.4453	0.6682
SO_RS04360	SO_0929	-0.5967	0.1940	0.4062
SO_RS04365	SO_0930	1.6306	0.0051	0.0670
SO_RS04370	SO_0931	-0.7453	0.6495	0.8393
SO_RS04375	SO_0932	0.2083	0.6802	0.8619
SO_RS04380	SO_0933	0.1378	0.8350	0.9761
SO_RS04385	SO_0934	0.4372	0.7867	0.9428
SO_RS04390	SO_0935	0.0413	0.9368	1.0000
SO_RS04395	SO_0936	0.0000	1.0000	1.0000
SO_RS04400	SO_0937	0.0000	1.0000	1.0000
SO_RS04405	SO_0938	2.9043	0.3261	0.5550
SO_RS04410	SO_0939	3.4321	0.2637	0.4849
SO_RS04415	SO_0940	0.8739	0.2702	0.4926
SO_RS04420	SO_0941	5.9026	0.0606	0.2196
SO_RS04425	SO_0942	-0.6461	0.5414	0.7453
SO_RS04430	SO_0943	-2.8818	0.0190	0.1206
SO_RS04435	SO_0944	1.2738	0.4567	0.6773
SO_RS04440	SO_0945	2.6136	0.1362	0.3314
SO_RS04445	SO_0946	8.2187	0.0082	0.0853
SO_RS04450		0.0000	1.0000	1.0000
SO_RS04455	SO_0947	0.3379	0.5575	0.7592
SO_RS04460	SO_0948	2.7370	0.1524	0.3524
SO_RS04465	SO_0949	-1.6914	0.0391	0.1725
SO_RS04470	SO_0950	-4.1985	0.0770	0.2438
SO_RS04475	SO_0951	0.7387	0.0482	0.1919
SO_RS04480	SO_0952	-2.6521	0.0172	0.1178
SO_RS04490	SO_0954	-0.1745	0.9322	1.0000
SO_RS04495	SO_0956	-3.8579	0.0041	0.0600
SO_RS04500	SO_0958	1.1957	0.0650	0.2260
SO_RS04505	SO_0959	1.7166	0.2809	0.5034
SO_RS04510	SO_0960	1.6786	0.4270	0.6559
SO_RS04515	SO_0961	2.7260	0.1194	0.3091
SO_RS04520	SO_0962	-3.2036	0.1596	0.3629
SO_RS04525	SO_0963	0.0000	1.0000	1.0000
SO_RS04530		-5.8377	0.0679	0.2314
SO_RS04535	SO_0966	0.2191	0.6839	0.8646
SO_RS04540		5.5445	0.0475	0.1910
SO_RS04545	SO_0968	2.0391	0.0107	0.0951
SO_RS04550	SO_0970	2.3119	0.0001	0.0087
SO_RS04555	SO_0971	4.9252	0.0942	0.2714
SO_RS04560	SO_0972	-3.1240	0.1664	0.3720
SO_RS04565	SO_0973	-4.1302	0.0876	0.2607

SO_RS15905	SO_3411	-0.8336	0.4004	0.6292
SO_RS15910	SO_3412	-2.2895	0.0901	0.2641
SO_RS15915	SO_3413	1.0396	0.1475	0.3466
SO_RS15920	SO_3414	1.8970	0.1896	0.4024
SO_RS15925	SO_3415	2.6795	0.0001	0.0095
SO_RS15930	SO_3417	0.6405	0.5939	0.7898
SO_RS15935	SO_3418	2.0908	0.5101	0.7216
SO_RS15940	SO_3419	2.0908	0.4421	0.6664
SO_RS15945	SO_3420	11.5107	0.0000	0.0040
SO_RS15950	SO_3421	-1.5201	0.0736	0.2405
SO_RS15955	SO_3422	1.2248	0.0602	0.2185
SO_RS15960	SO_3423	2.9942	0.1219	0.3128
SO_RS15965	SO_3424	-0.4096	0.3287	0.5576
SO_RS15970	SO_t083	0.0000	1.0000	1.0000
SO_RS15975	SO_t084	-6.0233	0.0614	0.2202
SO_RS15985	SO_t086	4.7498	0.2227	0.4427
SO_RS16010	SO_3426	0.0000	1.0000	1.0000
SO_RS16015	SO_3427	-1.4140	0.1455	0.3437
SO_RS16020	SO_3428	2.3766	0.0053	0.0675
SO_RS16025	SO_3429	3.0993	0.1153	0.3030
SO_RS16030	SO_3430	1.2416	0.0772	0.2438
SO_RS16035	SO_3431	0.4491	0.6873	0.8665
SO_RS16040	SO_3432	3.3396	0.0010	0.0307
SO_RS16045	SO_3433	-0.9048	0.6139	0.8066
SO_RS16050	SO_3434	-3.1042	0.1076	0.2903
SO_RS16055	SO_3435	-4.6140	0.0186	0.1205
SO_RS16060	SO_3436	-2.4227	0.1142	0.3017
SO_RS16065	SO_3437	4.5737	0.0933	0.2701
SO_RS16070	SO_3438	-8.6321	0.0042	0.0605
SO_RS16075	SO_3439	0.0000	1.0000	1.0000
SO_RS16080	SO_3440	-0.2164	0.6226	0.8145
SO_RS16085	SO_3441	-0.0668	0.9085	1.0000
SO_RS16090	SO_3442	8.1478	0.0044	0.0623
SO_RS16120	SO_3449	0.4097	0.7289	0.8991
SO_RS16125	SO_4841	4.5666	0.1273	0.3185
SO_RS16130		-6.5899	0.0310	0.1492
SO_RS16140		7.5841	0.0513	0.1976
SO_RS16145	SO_3454	1.9825	0.0298	0.1468
SO_RS16150	SO_3455	0.1657	0.6967	0.8743
SO_RS16155	SO_3456	-1.1230	0.2141	0.4330
SO_RS16160	SO_3457	1.7253	0.0789	0.2453
SO_RS16165	SO_3458	2.2782	0.2758	0.4983
SO_RS16170	SO_3459	0.0000	1.0000	1.0000
SO_RS16175	SO_3460	-0.3668	0.8360	0.9768
SO_RS16180	SO_3461	1.6861	0.3451	0.5726
SO_RS16185	SO_3462	0.9747	0.2071	0.4237
SO_RS16190	SO_3463	-5.4809	0.0107	0.0954
SO_RS16195	SO_3464	-0.7829	0.5552	0.7563
SO_RS16200	SO_3465	-2.1364	0.2147	0.4338
SO_RS16205	SO_3466	-0.4017	0.7247	0.8949
SO_RS16210	SO_3467	-0.8008	0.1894	0.4023
SO_RS16215	SO_3468	1.7654	0.3170	0.5445
SO_RS16220	SO_3469	-2.1688	0.1312	0.3240
SO_RS16225	SO_3470	8.7085	0.0178	0.1187
SO_RS16230	SO_3471	0.2902	0.6722	0.8557
SO_RS16235	SO_3472	2.0192	0.0026	0.0498
SO_RS16240	SO_3473	1.7743	0.4060	0.6353
SO_RS16245	SO_3474	-4.1326	0.1017	0.2828
SO_RS16250	SO_3475	8.0210	0.0571	0.2126
SO_RS16255	SO_3476	-0.6270	0.6723	0.8557
SO_RS16260		-2.4450	0.2026	0.4170
SO_RS16270		2.2036	0.1025	0.2842
SO_RS16275	SO_3480	3.4259	0.2611	0.4827
SO_RS16280	SO_3481	2.0860	0.4498	0.6703
SO_RS16285	SO_3483	5.5343	0.0745	0.2410
SO_RS16290	SO_3484	0.8526	0.5936	0.7897
SO_RS16295	SO_3485	0.0000	1.0000	1.0000
SO_RS16300	SO_3488	0.0000	1.0000	1.0000

SO_RS04570	SO_0974	-2.0211	0.0323	0.1520
SO_RS04575	SO_0975	1.6907	0.1365	0.3315
SO_RS04580	SO_0976	4.4738	0.0000	0.0042
SO_RS04585	SO_0977	1.8575	0.1627	0.3678
SO_RS04590	SO_0978	0.4252	0.7391	0.9058
SO_RS04595	SO_0980	-2.9034	0.1112	0.2959
SO_RS04600	SO_0981	-5.2827	0.0776	0.2438
SO_RS04610	SO_0984	-0.9719	0.5299	0.7359
SO_RS04615	SO_0986	-1.2053	0.5003	0.7138
SO_RS04620	SO_0987	1.7992	0.0739	0.2405
SO_RS04625	SO_0988	-0.5470	0.5452	0.7484
SO_RS04630	SO_0989	1.2819	0.1278	0.3190
SO_RS04635	SO_0990	1.6169	0.1462	0.3446
SO_RS04640	SO_0991	10.1080	0.0004	0.0200
SO_RS04645	SO_0992	-1.5749	0.0459	0.1869
SO_RS04650	SO_0994	0.0000	1.0000	1.0000
SO_RS04655	SO_0996	7.3547	0.0195	0.1217
SO_RS04660	SO_0997	8.8449	0.0034	0.0564
SO_RS04665	SO_0999	-1.8392	0.2595	0.4814
SO_RS04670	SO_1000	2.9757	0.1685	0.3744
SO_RS04675	SO_1001	1.2083	0.4588	0.6787
SO_RS04680	SO_1002	-2.2178	0.2151	0.4338
SO_RS04685	SO_1003	-0.2519	0.8679	0.9999
SO_RS04690	SO_1004	-1.6150	0.3295	0.5583
SO_RS04695	SO_1006	9.4436	0.0024	0.0485
SO_RS04700	SO_1007	0.6875	0.5945	0.7901
SO_RS04705	SO_1008	-4.8687	0.1338	0.3278
SO_RS04710	SO_1009	0.1422	0.8778	1.0000
SO_RS04715	SO_1010	1.7592	0.1412	0.3391
SO_RS04720	SO_1011	1.8981	0.1100	0.2946
SO_RS04725	SO_1012	-5.4826	0.0500	0.1948
SO_RS04730	SO_1013	0.3835	0.8204	0.9660
SO_RS04735	SO_1014	1.7882	0.2787	0.5010
SO_RS04740	SO_1015	0.9994	0.5313	0.7376
SO_RS04745	SO_1016	0.5788	0.5215	0.7309
SO_RS04750	SO_1017	1.5496	0.0738	0.2405
SO_RS04755	SO_1018	2.0019	0.1787	0.3877
SO_RS04760	SO_1019	-3.5883	0.0534	0.2039
SO_RS04765	SO_1020	-1.1216	0.5447	0.7482
SO_RS04770	SO_1021	-3.3419	0.1438	0.3429
SO_RS04780	SO_1026	9.9095	0.0004	0.0200
SO_RS04785	SO_1027	4.1041	0.2173	0.4377
SO_RS04790	SO_1028	0.0000	1.0000	1.0000
SO_RS04795	SO_1029	6.4261	0.0491	0.1935
SO_RS04800	SO_1030	-1.8340	0.0809	0.2489
SO_RS04805	SO_1031	-3.9336	0.0614	0.2202
SO_RS04810	SO_1033	0.5143	0.8054	0.9563
SO_RS04815	SO_1034	-2.3782	0.3163	0.5438
SO_RS04820	SO_1035	-0.3079	0.7572	0.9210
SO_RS04825	SO_1036	-1.0500	0.2298	0.4508
SO_RS04830	SO_1037	7.6782	0.0313	0.1497
SO_RS04835	SO_1038	-0.6659	0.7394	0.9058
SO_RS04840	SO_1039	-3.8127	0.1069	0.2894
SO_RS04845	SO_4816	2.3979	0.3067	0.5322
SO_RS04850	SO_1042	0.5827	0.7045	0.8800
SO_RS04855	SO_1043	0.0000	1.0000	1.0000
SO_RS04860	SO_1044	9.4998	0.0044	0.0623
SO_RS04865	SO_1045	-0.1458	0.8199	0.9660
SO_RS04870	SO_1046	10.1026	0.0011	0.0328
SO_RS04875	SO_1047	1.0784	0.0664	0.2291
SO_RS04880	SO_1048	1.2508	0.0288	0.1444
SO_RS04885	SO_1049	1.7078	0.3444	0.5724
SO_RS04890	SO_1051	2.0581	0.0088	0.0869
SO_RS04895	SO_1052	-0.3478	0.8243	0.9680
SO_RS04900	SO_1053	8.7759	0.0037	0.0583
SO_RS04905	SO_1054	0.0000	1.0000	1.0000
SO_RS04910	SO_1055	0.0730	0.9340	1.0000
SO_RS04915	SO_1056	3.5065	0.0167	0.1165
SO_RS16305	SO_3489	4.9704	0.0211	0.1268
SO_RS16310	SO_3490	-0.2904	0.8573	0.9927
SO_RS16315	SO_3491	-0.2634	0.8379	0.9779
SO_RS16320	SO_3492	0.4383	0.3819	0.6106
SO_RS16325	SO_3493	1.2252	0.1018	0.2828
SO_RS16330	SO_3494	4.6599	0.0000	0.0055
SO_RS16335	SO_3495	2.0963	0.2716	0.4934
SO_RS16340	SO_3496	0.9777	0.2967	0.5199
SO_RS16345	SO_3497	-0.1181	0.9454	1.0000
SO_RS16350		0.0000	1.0000	1.0000
SO_RS16355	SO_3499	0.0000	1.0000	1.0000
SO_RS16360		-5.4965	0.0560	0.2105
SO_RS16365	SO_3501	0.0000	1.0000	1.0000
SO_RS16370	SO_3502	2.3820	0.0305	0.1480
SO_RS16375	SO_3503	-2.5747	0.1825	0.3919
SO_RS16380	SO_3504	0.0000	1.0000	1.0000
SO_RS16385	SO_3505	1.9881	0.2372	0.4578
SO_RS16390	SO_3506	-3.6096	0.0475	0.1910
SO_RS16395	SO_3507	5.0646	0.0867	0.2591
SO_RS16400	SO_3509	-0.8118	0.5494	0.7516
SO_RS16410	SO_4804	0.0000	1.0000	1.0000
SO_RS16415	SO_3512	0.0000	1.0000	1.0000
SO_RS16420	SO_3513	1.6817	0.4679	0.6856
SO_RS16425	SO_3514	-0.5177	0.5736	0.7704
SO_RS16430	SO_3516	-0.4850	0.7935	0.9474
SO_RS16435	SO_3517	-1.6146	0.1052	0.2878
SO_RS16445	SO_3519	1.0261	0.5250	0.7329
SO_RS16450	SO_3520	-5.8363	0.0423	0.1781
SO_RS16455	SO_3521	-1.8626	0.1093	0.2928
SO_RS16465	SO_3523	-1.3629	0.5467	0.7487
SO_RS16470	SO_3524	-0.7708	0.6629	0.8481
SO_RS16475	SO_3525	0.6994	0.3553	0.5838
SO_RS16480	SO_3526	-0.1964	0.8674	0.9998
SO_RS16485	SO_3527	-2.0992	0.0531	0.2029
SO_RS16490	SO_3528	-2.4174	0.0223	0.1286
SO_RS16495	SO_3529	-1.5628	0.3828	0.6112
SO_RS16500	SO_3530	5.2065	0.0756	0.2420
SO_RS16505	SO_3531	-1.8434	0.3235	0.5516
SO_RS16510	SO_3532	-0.8354	0.0490	0.1935
SO_RS16515	SO_3533	-2.3925	0.0292	0.1457
SO_RS16520	SO_3534	-0.5514	0.6842	0.8646
SO_RS16525	SO_3537	-0.4841	0.6297	0.8217
SO_RS16530	SO_3538	-1.8770	0.2468	0.4691
SO_RS16535	SO_3539	4.0342	0.0071	0.0781
SO_RS16540	SO_3540	1.2371	0.5531	0.7545
SO_RS16545	SO_3541	-2.9866	0.0380	0.1691
SO_RS16550	SO_3542	-1.5244	0.0189	0.1205
SO_RS16555	SO_3543	-0.7095	0.5379	0.7427
SO_RS16565	SO_3545	2.5678	0.0000	0.0031
SO_RS16570	SO_3546	1.4730	0.2039	0.4193
SO_RS16575	SO_3547	1.5401	0.0528	0.2021
SO_RS16580	SO_3548	-4.2480	0.0627	0.2224
SO_RS16585	SO_3549	2.3952	0.0072	0.0787
SO_RS16590	SO_3550	0.5633	0.7182	0.8904
SO_RS16595	SO_3551	0.9851	0.4705	0.6882
SO_RS16600	SO_3552	-0.4764	0.3082	0.5347
SO_RS16605	SO_3553	-0.7973	0.2707	0.4926
SO_RS16610	SO_3554	-0.6942	0.7194	0.8908
SO_RS16615	SO_3555	0.7219	0.6927	0.8716
SO_RS16620	SO_3556	5.7650	0.0214	0.1271
SO_RS16625	SO_3557	0.0658	0.9702	1.0000
SO_RS16630	SO_3558	5.4168	0.0031	0.0542
SO_RS16635	SO_3559	2.3691	0.0001	0.0062
SO_RS16640	SO_3560	-1.4071	0.0026	0.0490
SO_RS16645	SO_3561	0.0000	1.0000	1.0000
SO_RS16650	SO_3562	1.9921	0.0235	0.1303
SO_RS16655	SO_3563	1.2304	0.4735	0.6905
SO_RS16660	SO_3564	-1.6926	0.0688	0.2324

SO_RS04920	SO_1059	0.6798	0.6718	0.8556
SO_RS04925	SO_1060	1.3332	0.1281	0.3197
SO_RS04930	SO_1061	-0.5848	0.7014	0.8785
SO_RS04935	SO_1062	-2.2558	0.1491	0.3484
SO_RS04940	SO_1063	3.3497	0.1413	0.3391
SO_RS04945	SO_1064	1.6996	0.0929	0.2695
SO_RS04950	SO_1065	-0.8393	0.1507	0.3511
SO_RS04955	SO_1066	0.1437	0.7769	0.9371
SO_RS04960	SO_1067	2.5680	0.0648	0.2260
SO_RS04965	SO_1068	-0.0196	0.9892	1.0000
SO_RS04970	SO_1069	-4.2908	0.1595	0.3629
SO_RS04975	SO_1070	-2.4962	0.2410	0.4613
SO_RS04980	SO_1071	0.6442	0.6565	0.8442
SO_RS04985	SO_1072	-0.1313	0.9158	1.0000
SO_RS04990	SO_1074	1.1225	0.5093	0.7212
SO_RS04995	SO_1075	0.4502	0.5161	0.7273
SO_RS05000	SO_1077	-3.1921	0.1328	0.3263
SO_RS05005		-5.2826	0.0835	0.2536
SO_RS05015	SO_1081	5.8197	0.0396	0.1736
SO_RS05025	SO_1085	-3.2631	0.2018	0.4158
SO_RS05030	SO_1086	-1.6751	0.4892	0.7036
SO_RS05035	SO_1087	-2.2521	0.1210	0.3115
SO_RS05040	SO_1090	-6.5540	0.0109	0.0960
SO_RS05045	SO_1091	-6.9933	0.0209	0.1265
SO_RS05050	SO_1092	2.7842	0.1407	0.3384
SO_RS05055	SO_1093	0.3808	0.8488	0.9863
SO_RS05060	SO_1094	11.0079	0.0007	0.0265
SO_RS05065	SO_1095	0.4749	0.4675	0.6852
SO_RS05070	SO_1096	8.7666	0.0065	0.0762
SO_RS05075	SO_1097	8.7198	0.0120	0.0991
SO_RS05080	SO_1098	8.2830	0.0117	0.0988
SO_RS05085	SO_1099	-3.3042	0.0798	0.2470
SO_RS05090	SO_1100	-2.6055	0.2357	0.4566
SO_RS05095	SO_1101	-1.0535	0.3408	0.5692
SO_RS05100	SO_1102	4.1997	0.0088	0.0869
SO_RS05105	SO_1103	-0.5832	0.1683	0.3744
SO_RS05110	SO_1104	0.6256	0.1633	0.3679
SO_RS05115	SO_1105	1.4061	0.0245	0.1324
SO_RS05120	SO_1106	1.7194	0.1840	0.3946
SO_RS05125	SO_1107	1.6857	0.0748	0.2414
SO_RS05130	SO_1108	2.0592	0.0002	0.0133
SO_RS05135	SO_1109	-0.5584	0.7469	0.9115
SO_RS05140	SO_1110	-0.7681	0.5271	0.7334
SO_RS05145	SO_1111	3.8798	0.0017	0.0422
SO_RS05150	SO_1112	2.3838	0.0028	0.0502
SO_RS05155	SO_1113	0.8843	0.5203	0.7304
SO_RS05160	SO_1114	2.9929	0.0217	0.1279
SO_RS05165	SO_1115	0.8355	0.2043	0.4198
SO_RS05170	SO_1117	0.4874	0.5513	0.7528
SO_RS05175	SO_1120	-0.0980	0.9540	1.0000
SO_RS05180	SO_1121	0.2558	0.7770	0.9371
SO_RS05185	SO_1122	0.4610	0.6926	0.8716
SO_RS05190	SO_1123	-0.9098	0.6637	0.8486
SO_RS05195	SO_1124	2.0860	0.4584	0.6787
SO_RS05200	SO_1125	4.3527	0.1747	0.3827
SO_RS05205	SO_1126	0.9452	0.0068	0.0772
SO_RS05210	SO_1127	0.5963	0.1386	0.3348
SO_RS05220		-7.4450	0.0217	0.1279
SO_RS05230	SO_1135	-4.1404	0.0168	0.1172
SO_RS05235	SO_1136	5.4077	0.0105	0.0944
SO_RS05240	SO_1137	1.2849	0.3624	0.5909
SO_RS05250	SO_1139	1.1246	0.1205	0.3109
SO_RS05255	SO_1140	1.5741	0.1866	0.3983
SO_RS05260	SO_1141	-2.0790	0.0507	0.1964
SO_RS05265	SO_1142	-0.3054	0.4207	0.6492
SO_RS05270	SO_1143	5.4298	0.0822	0.2514
SO_RS05275	SO_1144	4.3982	0.0134	0.1050
SO_RS05280	SO_1145	0.8993	0.6405	0.8317

SO_RS16665	SO_3565	-1.5485	0.3805	0.6094
SO_RS16670		-10.1455	0.0195	0.1217
SO_RS16685	SO_3571	9.3243	0.0018	0.0424
SO_RS16695	SO_r014	-7.3825	0.0458	0.1868
SO_RS16700	SO_r015	-2.7411	0.3262	0.5550
SO_RS16705	SO_3575	-0.3904	0.6365	0.8283
SO_RS16710	SO_3576	2.9421	0.0847	0.2558
SO_RS16715	SO_3577	2.2623	0.0000	0.0036
SO_RS16720	SO_3578	-0.6642	0.7206	0.8913
SO_RS16725	SO_3579	0.4772	0.7620	0.9254
SO_RS16730	SO_3580	-2.0103	0.0106	0.0948
SO_RS16750	SO_3582	-0.8775	0.2495	0.4709
SO_RS16755	SO_3583	-2.5688	0.2178	0.4380
SO_RS16760	SO_3584	-0.8858	0.3832	0.6115
SO_RS16765	SO_3585	-2.8903	0.1417	0.3395
SO_RS16770	SO_3586	-1.1022	0.4883	0.7036
SO_RS16775	SO_3587	-2.5504	0.2194	0.4393
SO_RS16780	SO_3588	-4.2171	0.0747	0.2413
SO_RS16785	SO_3591	-0.1817	0.9281	1.0000
SO_RS16790	SO_3592	-4.3745	0.0120	0.0991
SO_RS16795	SO_3593	-2.0618	0.2705	0.4926
SO_RS16800	SO_3594	1.1961	0.4776	0.6950
SO_RS16805	SO_3595	-5.2184	0.0390	0.1722
SO_RS16810	SO_3596	-4.2470	0.0585	0.2159
SO_RS16815	SO_3597	4.5344	0.0024	0.0485
SO_RS16820	SO_3598	3.5741	0.0000	0.0039
SO_RS16825	SO_3599	6.7015	0.0001	0.0096
SO_RS16830	SO_3600	3.4402	0.0285	0.1436
SO_RS16835	SO_3601	1.4062	0.3933	0.6220
SO_RS16840	SO_3602	11.2111	0.0003	0.0149
SO_RS16845		-7.5517	0.0003	0.0149
SO_RS16850	SO_3604	-10.6230	0.0235	0.1303
SO_RS16860		8.2537	0.0421	0.1779
SO_RS16870		4.5772	0.2100	0.4276
SO_RS16875	SO_3611	0.0381	0.9812	1.0000
SO_RS16880	SO_3613	-0.0803	0.9553	1.0000
SO_RS16885	SO_3614	5.5332	0.1120	0.2977
SO_RS16890	SO_3615	-1.2579	0.1599	0.3631
SO_RS16900	SO_3618	-8.3621	0.0064	0.0762
SO_RS16905	SO_3621	-6.6120	0.0458	0.1868
SO_RS16910	SO_3622	3.4228	0.3614	0.5898
SO_RS16915	SO_3623	0.0000	1.0000	1.0000
SO_RS16920		0.8142	0.6424	0.8329
SO_RS16935	SO_3627	-1.2560	0.5491	0.7514
SO_RS16940	SO_3628	4.1152	0.2273	0.4490
SO_RS16945	SO_3629	-6.1872	0.0371	0.1664
SO_RS16955	SO_3631	0.1007	0.9323	1.0000
SO_RS16960	SO_3632	-3.2703	0.1083	0.2907
SO_RS16965	SO_3633	-5.9729	0.0043	0.0619
SO_RS16970	SO_3634	-4.1011	0.0956	0.2733
SO_RS16975	SO_3635	9.4627	0.0011	0.0334
SO_RS16980	SO_3636	-0.5263	0.3715	0.6011
SO_RS16985	SO_3637	-3.4634	0.0134	0.1050
SO_RS16990	SO_3638	1.1543	0.4125	0.6407
SO_RS16995	SO_3639	-4.3521	0.0019	0.0435
SO_RS17000	SO_3640	0.0000	1.0000	1.0000
SO_RS17005	SO_3641	-4.3387	0.0330	0.1541
SO_RS17010	SO_3642	8.5810	0.0113	0.0981
SO_RS17015	SO_3645	-3.3886	0.1700	0.3769
SO_RS17020	SO_3646	0.8199	0.6189	0.8108
SO_RS17025	SO_3647	-2.4035	0.2308	0.4518
SO_RS17030	SO_3648	-1.6478	0.2839	0.5077
SO_RS17035	SO_3649	0.0354	0.9452	1.0000
SO_RS17040	SO_3651	-2.1973	0.0869	0.2596
SO_RS17045	SO_3652	-1.6274	0.0854	0.2571
SO_RS17050	SO_3653	1.0932	0.1017	0.2828
SO_RS17055	SO_3654	-1.9873	0.1805	0.3895
SO_RS17060	SO_3655	-3.4776	0.0784	0.2453

SO_RS05285	SO_1146	0.0000	1.0000	1.0000
SO_RS05290	SO_1148	-4.4103	0.0499	0.1948
SO_RS05295	SO_1149	1.6090	0.1242	0.3151
SO_RS05300	SO_1150	0.2155	0.8849	1.0000
SO_RS05305	SO_1151	-0.0390	0.9650	1.0000
SO_RS05310	SO_1153	-3.8131	0.1234	0.3141
SO_RS05315	SO_1154	0.0366	0.9796	1.0000
SO_RS05325	SO_1155	4.9135	0.1365	0.3315
SO_RS05330	SO_1156	2.5402	0.0891	0.2632
SO_RS05335	SO_1157	-2.0078	0.1262	0.3176
SO_RS05340	SO_1158	-0.5187	0.5718	0.7693
SO_RS05345	SO_1159	7.1303	0.0658	0.2276
SO_RS05350	SO_1160	3.4228	0.2614	0.4829
SO_RS05355	SO_1161	1.8609	0.1527	0.3524
SO_RS05360	SO_1162	1.1117	0.2532	0.4741
SO_RS05365	SO_1163	-6.0208	0.0305	0.1481
SO_RS05370	SO_1164	-3.0442	0.0001	0.0096
SO_RS05375	SO_1165	3.5471	0.0635	0.2231
SO_RS05380	SO_1166	-0.1463	0.9148	1.0000
SO_RS05385	SO_1167	-4.4257	0.0608	0.2196
SO_RS05390	SO_1168	5.2028	0.0632	0.2231
SO_RS05395	SO_1169	-4.7870	0.0178	0.1187
SO_RS05400	SO_1170	-3.7672	0.0607	0.2196
SO_RS05405	SO_1171	6.4795	0.0506	0.1962
SO_RS05410	SO_1172	-7.2579	0.0149	0.1102
SO_RS05415	SO_1173	-0.1534	0.9458	1.0000
SO_RS05420	SO_1174	-0.4459	0.3100	0.5365
SO_RS05425	SO_1175	1.8086	0.0221	0.1283
SO_RS05430	SO_1176	-2.8843	0.1940	0.4062
SO_RS05435	SO_1177	-1.3271	0.3894	0.6185
SO_RS05440	SO_1178	0.9385	0.4372	0.6636
SO_RS05445	SO_1179	6.2375	0.0510	0.1971
SO_RS05450	SO_1180	0.6061	0.4082	0.6371
SO_RS05455	SO_1181	-0.3291	0.7830	0.9418
SO_RS05460	SO_1182	4.3527	0.1775	0.3864
SO_RS05465	SO_1183	0.5819	0.5462	0.7486
SO_RS05470	SO_1184	-0.0318	0.9767	1.0000
SO_RS05475	SO_1185	0.2112	0.7675	0.9287
SO_RS05515	SO_1187	0.0000	1.0000	1.0000
SO_RS05520	SO_1188	0.0000	1.0000	1.0000
SO_RS05525	SO_1189	6.8878	0.0426	0.1786
SO_RS05530	SO_1190	2.3465	0.0447	0.1842
SO_RS05535	SO_1191	0.6675	0.5811	0.7774
SO_RS05540	SO_1192	-0.3041	0.6885	0.8675
SO_RS05545	SO_1193	-3.6064	0.0652	0.2262
SO_RS05550	SO_1194	-0.6286	0.6476	0.8389
SO_RS05555	SO_1195	4.7608	0.1260	0.3176
SO_RS05560	SO_1196	-0.5660	0.2538	0.4748
SO_RS05565	SO_1197	-0.6392	0.0740	0.2405
SO_RS05570	SO_1198	0.9869	0.5748	0.7712
SO_RS05575	SO_1199	0.3401	0.5999	0.7938
SO_RS05580	SO_1200	-1.4636	0.0953	0.2732
SO_RS05585	SO_1201	-0.6165	0.6522	0.8409
SO_RS05600	SO_1202	-3.7498	0.0218	0.1279
SO_RS05605	SO_1203	0.0191	0.9662	1.0000
SO_RS05610	SO_1204	1.4915	0.0008	0.0269
SO_RS05615	SO_1205	2.9065	0.0610	0.2201
SO_RS05620	SO_1206	0.7909	0.4915	0.7058
SO_RS05625	SO_1207	-0.3382	0.7785	0.9387
SO_RS05630	SO_1208	1.2409	0.0976	0.2763
SO_RS05635	SO_1209	1.4317	0.0020	0.0443
SO_RS05640	SO_1210	0.4132	0.5275	0.7337
SO_RS05645	SO_1211	1.2448	0.2254	0.4464
SO_RS05650	SO_1213	0.0000	1.0000	1.0000
SO_RS05655	SO_1214	-0.0677	0.9187	1.0000
SO_RS05660	SO_1215	0.5168	0.2936	0.5169
SO_RS05665	SO_1217	-1.0525	0.0986	0.2779
SO_RS05670	SO_1218	-0.6520	0.3490	0.5767

SO_RS17065	SO_3656	4.7545	0.0738	0.2405
SO_RS17070	SO_3657	-2.3406	0.2633	0.4849
SO_RS17075	SO_3659	6.8514	0.0759	0.2422
SO_RS17080	SO_3660	11.3783	0.0002	0.0136
SO_RS17085	SO_3662	0.9135	0.6547	0.8428
SO_RS17090	SO_3664	2.2529	0.0301	0.1469
SO_RS17095	SO_3665	2.8707	0.1016	0.2828
SO_RS17100	SO_3667	1.7746	0.2905	0.5134
SO_RS17105	SO_3668	0.1082	0.9427	1.0000
SO_RS17110	SO_3669	-0.8692	0.2207	0.4408
SO_RS17115	SO_3670	-4.2503	0.0357	0.1613
SO_RS17120	SO_3671	-5.0742	0.0431	0.1792
SO_RS17125	SO_3672	0.0000	1.0000	1.0000
SO_RS17130	SO_3673	5.4297	0.0120	0.0991
SO_RS17135	SO_3674	-0.5156	0.7904	0.9456
SO_RS17140	SO_3675	-1.2285	0.5215	0.7309
SO_RS17145	SO_3676	2.0908	0.4451	0.6682
SO_RS17150	SO_3677	-10.4728	0.0139	0.1072
SO_RS17155	SO_3678	-0.6072	0.5687	0.7674
SO_RS17160	SO_3679	0.7502	0.6501	0.8393
SO_RS17165	SO_3680	-1.6960	0.3376	0.5654
SO_RS17170	SO_3681	4.4697	0.0000	0.0033
SO_RS17175	SO_3682	2.1922	0.0006	0.0244
SO_RS17180	SO_3683	4.2707	0.0094	0.0899
SO_RS17185	SO_3684	1.2301	0.4579	0.6784
SO_RS17190	SO_3685	0.0000	1.0000	1.0000
SO_RS17195	SO_3686	0.0000	1.0000	1.0000
SO_RS17200	SO_3687	-2.4661	0.2890	0.5114
SO_RS17205	SO_3688	-1.6490	0.4716	0.6896
SO_RS17210	SO_3689	-2.6348	0.0310	0.1492
SO_RS17215	SO_3690	2.6653	0.0775	0.2438
SO_RS17220	SO_3691	6.5259	0.0166	0.1163
SO_RS17225	SO_3692	6.1859	0.0425	0.1785
SO_RS17230	SO_3693	1.0780	0.6269	0.8182
SO_RS17235	SO_3694	1.6361	0.3500	0.5780
SO_RS17240	SO_3695	8.6063	0.0050	0.0667
SO_RS17245	SO_3696	5.0641	0.0114	0.0981
SO_RS17250	SO_3697	0.0000	1.0000	1.0000
SO_RS17255	SO_3698	-3.9487	0.0228	0.1291
SO_RS17260	SO_4805	-2.8751	0.1146	0.3022
SO_RS17265	SO_3700	-2.4156	0.1062	0.2889
SO_RS17270	SO_3701	-2.3970	0.2307	0.4518
SO_RS17275	SO_3704	0.0000	1.0000	1.0000
SO_RS17280	SO_3705	8.8105	0.0053	0.0676
SO_RS17285	SO_3706	-3.5859	0.0670	0.2293
SO_RS17290	SO_3708	-5.2241	0.0368	0.1652
SO_RS17295	SO_3709	3.8111	0.1522	0.3524
SO_RS17300	SO_3711	-2.0574	0.0769	0.2438
SO_RS17305	SO_3712	-11.0104	0.0228	0.1291
SO_RS17310	SO_3713	-0.1908	0.9238	1.0000
SO_RS17315	SO_3714	3.4410	0.0001	0.0089
SO_RS17320	SO_3715	1.5000	0.2185	0.4385
SO_RS17325	SO_3716	4.2122	0.0183	0.1201
SO_RS17330	SO_3718	-2.5991	0.0882	0.2620
SO_RS17335	SO_3719	0.0000	1.0000	1.0000
SO_RS17340	SO_3720	-3.5541	0.0966	0.2751
SO_RS17345	SO_3722	-0.2603	0.8804	1.0000
SO_RS17350	SO_3723	3.6485	0.0356	0.1613
SO_RS17355	SO_3724	1.8889	0.2645	0.4856
SO_RS17360	SO_3726	3.0920	0.0038	0.0583
SO_RS17365	SO_3727	5.6429	0.0016	0.0400
SO_RS17370	SO_3728	4.3703	0.0021	0.0459
SO_RS17375	SO_3729	-3.6250	0.0331	0.1541
SO_RS17380	SO_3731	-2.4299	0.0814	0.2496
SO_RS17385	SO_3733	1.9094	0.1018	0.2828
SO_RS17390	SO_4806	-1.5632	0.3644	0.5932
SO_RS17395	SO_3735	5.7264	0.0357	0.1615
SO_RS17400	SO_3736	2.5676	0.0066	0.0765

SO_RS05675	SO_1219	-0.3469	0.5976	0.7927
SO_RS05680	SO_1221	0.9438	0.1715	0.3791
SO_RS05685	SO_1222	-2.9936	0.0132	0.1044
SO_RS05690	SO_1223	-2.5105	0.1393	0.3363
SO_RS05695	SO_1224	0.5239	0.7327	0.9003
SO_RS05700	SO_1225	-0.0104	0.9926	1.0000
SO_RS05705	SO_1226	0.1945	0.8141	0.9622
SO_RS05710	SO_1227	-1.0077	0.4626	0.6818
SO_RS05715	SO_1228	0.4739	0.6701	0.8546
SO_RS05720	SO_1229	0.1777	0.9122	1.0000
SO_RS05725	SO_1230	-2.4344	0.0220	0.1279
SO_RS05730	SO_1231	4.1131	0.1525	0.3524
SO_RS05735	SO_1232	-2.2885	0.0788	0.2453
SO_RS05740	SO_1233	8.3127	0.0500	0.1948
SO_RS05750	SO_1236	-1.7620	0.3538	0.5827
SO_RS05755	SO_1237	5.0285	0.0035	0.0568
SO_RS05760	SO_1238	2.5661	0.1803	0.3895
SO_RS05765	SO_1239	3.4320	0.2556	0.4770
SO_RS05770	SO_1241	-5.3817	0.0341	0.1573
SO_RS05780	SO_1242	-2.4544	0.0224	0.1286
SO_RS05785	SO_1244	2.9043	0.4084	0.6371
SO_RS05790	SO_1245	2.5515	0.0866	0.2591
SO_RS05795	SO_1247	-1.0422	0.3851	0.6138
SO_RS05800	SO_1248	7.0425	0.0623	0.2215
SO_RS05805	SO_1249	7.5343	0.0521	0.1998
SO_RS05810	SO_1250	-0.1608	0.8890	1.0000
SO_RS05815	SO_1251	2.0861	0.4660	0.6843
SO_RS05820	SO_1252	0.4958	0.5750	0.7713
SO_RS05825	SO_1254	0.8549	0.4391	0.6641
SO_RS05830	SO_1255	-1.0442	0.1565	0.3579
SO_RS05835	SO_1256	-4.2964	0.0859	0.2577
SO_RS05840	SO_1258	-0.0047	0.9906	1.0000
SO_RS05845	SO_1259	9.1723	0.0118	0.0990
SO_RS05850	SO_1260	-4.2428	0.1027	0.2842
SO_RS05855	SO_1261	0.9038	0.3695	0.5985
SO_RS05860	SO_1262	9.3440	0.0038	0.0583
SO_RS05865	SO_1264	-1.4010	0.2616	0.4829
SO_RS05870	SO_1265	-3.2939	0.0899	0.2640
SO_RS05875	SO_1267	0.8652	0.0934	0.2701
SO_RS05880	SO_1268	0.5425	0.3427	0.5706
SO_RS05885	SO_1269	-5.8938	0.0412	0.1766
SO_RS05890	SO_1270	2.3953	0.0486	0.1928
SO_RS05895	SO_1271	5.3326	0.0100	0.0932
SO_RS05900	SO_1272	-2.2801	0.0910	0.2657
SO_RS05905	SO_1273	1.4834	0.1510	0.3513
SO_RS05910	SO_1274	-1.4428	0.0263	0.1382
SO_RS05915	SO_1275	-1.1753	0.0465	0.1884
SO_RS05920	SO_1276	0.5220	0.2602	0.4819
SO_RS05925	SO_1277	1.6476	0.2742	0.4964
SO_RS05930	SO_1278	-0.2090	0.6637	0.8486
SO_RS05935		0.0000	1.0000	1.0000
SO_RS05940	SO_1280	7.4459	0.0558	0.2102
SO_RS05945	SO_1281	0.0000	1.0000	1.0000
SO_RS05960	SO_1284	0.1779	0.6504	0.8393
SO_RS05965	SO_1286	-2.6385	0.0109	0.0960
SO_RS05970	SO_1287	-1.1817	0.0233	0.1302
SO_RS05975	SO_1288	-7.9789	0.0057	0.0706
SO_RS05980	SO_1289	-0.0867	0.9155	1.0000
SO_RS05985	SO_1290	-0.3680	0.7969	0.9496
SO_RS05990	SO_1291	-0.6481	0.7024	0.8789
SO_RS05995	SO_1292	2.3230	0.2457	0.4674
SO_RS06000	SO_1293	0.1345	0.9184	1.0000
SO_RS06005	SO_1294	6.2520	0.0804	0.2482
SO_RS06010	SO_1295	-0.8699	0.1743	0.3824
SO_RS06015	SO_1296	0.8731	0.4809	0.6972
SO_RS06020	SO_1297	-1.5095	0.0635	0.2231
SO_RS06025	SO_1298	-0.6412	0.6128	0.8057
SO_RS06030	SO_1299	4.2142	0.0897	0.2640

SO_RS17405	SO_3737	4.8542	0.0002	0.0112
SO_RS17410	SO_3738	3.0913	0.0000	0.0033
SO_RS17415	SO_3740	-0.2173	0.6760	0.8583
SO_RS17420	SO_3741	1.7001	0.0186	0.1205
SO_RS17425	SO_4807	2.0050	0.3727	0.6023
SO_RS17430	SO_3743	2.9944	0.0189	0.1205
SO_RS17435	SO_3744	-5.9738	0.0293	0.1457
SO_RS17440	SO_3745	-2.1040	0.0385	0.1709
SO_RS17445	SO_3746	-0.9170	0.5255	0.7331
SO_RS17450	SO_3747	-2.7580	0.0036	0.0579
SO_RS17455	SO_3748	-0.2713	0.8374	0.9778
SO_RS17460	SO_3749	-0.6325	0.5060	0.7179
SO_RS17465	SO_3750	-2.5087	0.2163	0.4360
SO_RS17470		-4.0981	0.0699	0.2342
SO_RS17475		0.0000	1.0000	1.0000
SO_RS17485		0.0000	1.0000	1.0000
SO_RS17490		0.0000	1.0000	1.0000
SO_RS17500	SO_3756	-0.1897	0.9123	1.0000
SO_RS17505	SO_3757	8.5428	0.0383	0.1700
SO_RS17510	SO_3758	0.6054	0.7310	0.8999
SO_RS17515	SO_3759	-0.6812	0.4410	0.6659
SO_RS17520	SO_3760	0.7537	0.1581	0.3607
SO_RS17525	SO_3761	0.2058	0.7991	0.9513
SO_RS17530	SO_3762	2.7551	0.1657	0.3715
SO_RS17535	SO_3763	-1.6349	0.1176	0.3067
SO_RS17540	SO_3764	-1.1371	0.1041	0.2860
SO_RS17545	SO_3765	0.1626	0.8210	0.9660
SO_RS17550	SO_3766	5.8344	0.0010	0.0309
SO_RS17555	SO_3767	-0.5188	0.6927	0.8716
SO_RS17560	SO_3768	-0.8402	0.5899	0.7867
SO_RS17565	SO_3769	0.9011	0.2406	0.4612
SO_RS17570	SO_3770	2.6226	0.1233	0.3141
SO_RS17575	SO_3771	0.2379	0.7215	0.8919
SO_RS17580	SO_3772	-0.1985	0.8325	0.9735
SO_RS17585	SO_3774	-0.1024	0.8268	0.9687
SO_RS17590	SO_3775	2.5077	0.1207	0.3110
SO_RS17595	SO_3776	-0.1502	0.9405	1.0000
SO_RS17600	SO_3777	0.0000	1.0000	1.0000
SO_RS17605	SO_3778	-2.2548	0.1539	0.3535
SO_RS17610	SO_3779	0.9832	0.2723	0.4940
SO_RS17615	SO_3780	0.7929	0.4102	0.6388
SO_RS17620	SO_3781	-6.3814	0.0132	0.1044
SO_RS17625	SO_3782	0.6039	0.5365	0.7415
SO_RS17630	SO_3783	3.4101	0.1803	0.3895
SO_RS17635	SO_3786	3.6776	0.0066	0.0765
SO_RS17640	SO_3787	0.3377	0.5396	0.7437
SO_RS17645	SO_3789	0.4557	0.7722	0.9324
SO_RS17650	SO_3790	4.1041	0.2546	0.4757
SO_RS17655	SO_3791	0.7489	0.6524	0.8409
SO_RS17660	SO_3792	-1.8382	0.2327	0.4533
SO_RS17665	SO_3793	0.0000	1.0000	1.0000
SO_RS17670	SO_3794	-0.6370	0.5397	0.7437
SO_RS17675	SO_3795	4.1154	0.1912	0.4040
SO_RS17680	SO_3796	-1.0667	0.4641	0.6831
SO_RS17685	SO_3797	1.4131	0.2233	0.4435
SO_RS17690	SO_3798	-9.1915	0.0012	0.0346
SO_RS17695	SO_3799	-1.5585	0.3608	0.5896
SO_RS17700	SO_3800	1.3171	0.0833	0.2536
SO_RS17705	SO_3801	0.7487	0.6255	0.8172
SO_RS17710	SO_3802	0.5001	0.4352	0.6616
SO_RS17715	SO_3803	-0.7766	0.4830	0.6986
SO_RS17720	SO_3804	-3.4179	0.0162	0.1153
SO_RS17725	SO_3805	0.1046	0.8651	0.9990
SO_RS17730	SO_3807	-4.2518	0.0313	0.1497
SO_RS17735	SO_3808	-0.4572	0.6986	0.8756
SO_RS17740	SO_3810	-2.0265	0.2525	0.4738
SO_RS17745	SO_3811	-1.9954	0.2979	0.5216
SO_RS17750	SO_3812	-0.6060	0.6411	0.8322

SO_RS06035	SO_1300	1.2878	0.0737	0.2405
SO_RS06040	SO_1301	-1.3730	0.2387	0.4590
SO_RS06045	SO_1302	8.8419	0.0030	0.0535
SO_RS06050	SO_1303	-2.4983	0.1454	0.3437
SO_RS06055	SO_1304	0.3265	0.7509	0.9157
SO_RS06060	SO_1305	3.4289	0.1949	0.4073
SO_RS06065	SO_1306	-5.8740	0.0137	0.1061
SO_RS06070	SO_1307	3.0610	0.0797	0.2469
SO_RS06075	SO_1309	0.2098	0.8595	0.9948
SO_RS06080	SO_1310	-3.7478	0.0839	0.2543
SO_RS06085	SO_1311	-3.8197	0.0900	0.2640
SO_RS06090	SO_1313	-3.6174	0.0174	0.1182
SO_RS06095	SO_1314	2.8637	0.0283	0.1436
SO_RS06100	SO_1315	1.0396	0.1525	0.3524
SO_RS06105	SO_1317	-3.1084	0.0015	0.0387
SO_RS06110	SO_1318	-3.1564	0.1448	0.3437
SO_RS06115	SO_1319	1.7068	0.3755	0.6050
SO_RS06120	SO_1320	2.0909	0.4458	0.6686
SO_RS06125	SO_1321	-1.3369	0.5224	0.7313
SO_RS06130	SO_1322	-0.6417	0.6953	0.8731
SO_RS06135	SO_1324	0.2146	0.8044	0.9557
SO_RS06140	SO_1325	-0.4363	0.4196	0.6482
SO_RS06145	SO_1326	-1.1602	0.5257	0.7331
SO_RS06150	SO_1327	-0.2507	0.7901	0.9455
SO_RS06155	SO_1328	1.0302	0.3589	0.5882
SO_RS06160	SO_1329	-1.0309	0.5355	0.7405
SO_RS06165	SO_1330	-4.1183	0.0202	0.1239
SO_RS06170	SO_1331	-0.6075	0.4051	0.6347
SO_RS06175	SO_1332	1.1104	0.2283	0.4503
SO_RS06180	SO_1333	2.9527	0.0811	0.2490
SO_RS06185	SO_1334	-1.1078	0.4648	0.6831
SO_RS06190	SO_1335	3.1182	0.0311	0.1492
SO_RS06195	SO_1336	1.2855	0.2713	0.4932
SO_RS06200	SO_1337	3.6902	0.0023	0.0465
SO_RS06205	SO_1338	-0.2113	0.8651	0.9990
SO_RS06210	SO_1339	4.7513	0.1780	0.3868
SO_RS06215	SO_1340	6.5176	0.0596	0.2172
SO_RS06220	SO_1341	-0.4816	0.4681	0.6857
SO_RS06225	SO_1342	-1.3232	0.0076	0.0816
SO_RS06230	SO_1343	-1.5078	0.0267	0.1386
SO_RS06235	SO_1344	-3.6620	0.0005	0.0222
SO_RS06240	SO_1345	-0.2730	0.8300	0.9711
SO_RS06245	SO_1346	-0.9348	0.3286	0.5576
SO_RS06250	SO_1347	-1.9375	0.0693	0.2331
SO_RS06255	SO_1348	-0.3344	0.8159	0.9637
SO_RS06260	SO_1349	0.2345	0.8180	0.9644
SO_RS06265	SO_1350	-3.4410	0.0415	0.1767
SO_RS06270	SO_1351	8.1715	0.0069	0.0772
SO_RS06275	SO_1352	0.0000	1.0000	1.0000
SO_RS06280	SO_1353	2.0243	0.0416	0.1767
SO_RS06285	SO_1354	-3.1974	0.1233	0.3141
SO_RS06290	SO_1355	-0.8618	0.3580	0.5876
SO_RS06295	SO_1356	-0.2776	0.6227	0.8145
SO_RS06300	SO_1357	-0.4404	0.8004	0.9525
SO_RS06305	SO_1358	-2.1114	0.0035	0.0568
SO_RS06310	SO_1359	-1.2949	0.0154	0.1121
SO_RS06315	SO_1360	-0.5465	0.6658	0.8501
SO_RS06320	SO_1361	4.3381	0.0118	0.0990
SO_RS06325	SO_1362	-0.9761	0.3880	0.6169
SO_RS06330	SO_1363	-0.2823	0.7700	0.9307
SO_RS06335	SO_1364	0.0000	1.0000	1.0000
SO_RS06340	SO_1365	3.5480	0.0663	0.2289
SO_RS06345	SO_1366	2.6620	0.0165	0.1161
SO_RS06350	SO_1367	3.4726	0.0143	0.1084
SO_RS06355	SO_1368	0.3492	0.8179	0.9644
SO_RS06360	SO_1369	-5.7169	0.0373	0.1670
SO_RS06365	SO_1370	0.5800	0.7331	0.9006
SO_RS06370	SO_1371	-3.3044	0.1080	0.2905

SO_RS17755	SO_3813	0.2085	0.8727	1.0000
SO_RS17760	SO_3814	8.7200	0.0105	0.0944
SO_RS17765	SO_3815	2.3282	0.1802	0.3895
SO_RS17770	SO_3816	0.0000	1.0000	1.0000
SO_RS17775	SO_3817	-5.9895	0.0342	0.1575
SO_RS17780	SO_3819	5.1953	0.0977	0.2764
SO_RS17785	SO_3820	-7.1599	0.0201	0.1233
SO_RS17790	SO_3821	-3.0396	0.1227	0.3141
SO_RS17795	SO_3822	-3.3756	0.0263	0.1382
SO_RS17805		-6.8336	0.0480	0.1919
SO_RS17820	SO_3826	0.0000	1.0000	1.0000
SO_RS17825	SO_3827	1.3412	0.0482	0.1919
SO_RS17830	SO_3828	-2.0165	0.1016	0.2828
SO_RS17835	SO_3829	0.5090	0.7833	0.9418
SO_RS17840	SO_3830	6.8407	0.0169	0.1172
SO_RS17845	SO_3831	5.6306	0.1107	0.2954
SO_RS17850	SO_3832	-3.2446	0.0495	0.1945
SO_RS17855	SO_3833	-0.9930	0.4396	0.6643
SO_RS17860	SO_3834	-0.5786	0.5471	0.7491
SO_RS17865	SO_3835	-4.2043	0.0744	0.2407
SO_RS17870	SO_3836	0.3030	0.8851	1.0000
SO_RS17875	SO_3837	0.6979	0.2603	0.4819
SO_RS17880	SO_3838	-1.4723	0.0376	0.1676
SO_RS17885	SO_3840	-2.2960	0.1610	0.3652
SO_RS17890	SO_3841	1.5257	0.0706	0.2352
SO_RS17895	SO_3842	0.6872	0.4412	0.6659
SO_RS17900	SO_3843	8.8942	0.0021	0.0460
SO_RS17905	SO_3844	1.8299	0.0114	0.0981
SO_RS17910	SO_3846	0.3955	0.6610	0.8463
SO_RS17915	SO_3847	-3.5989	0.1473	0.3464
SO_RS17920	SO_3848	-4.6829	0.0635	0.2231
SO_RS17925	SO_3849	7.5840	0.0323	0.1521
SO_RS17935	SO_3852	2.6897	0.1300	0.3221
SO_RS17940	SO_3854	-1.5292	0.4698	0.6875
SO_RS17945	SO_3855	-0.8625	0.4962	0.7104
SO_RS17950	SO_3856	0.0000	1.0000	1.0000
SO_RS17955	SO_3857	-3.6972	0.0405	0.1753
SO_RS17960	SO_3858	0.0000	1.0000	1.0000
SO_RS17965	SO_3859	-2.0416	0.1981	0.4103
SO_RS17970	SO_3860	-3.5806	0.0337	0.1562
SO_RS17975	SO_3861	3.7909	0.0449	0.1849
SO_RS17980	SO_3862	-1.7095	0.2842	0.5079
SO_RS17985	SO_3863	2.7466	0.0318	0.1510
SO_RS17990	SO_3864	-4.0900	0.0736	0.2405
SO_RS17995	SO_3865	-6.6088	0.0293	0.1457
SO_RS18000	SO_3866	-0.5373	0.7119	0.8854
SO_RS18010	SO_3868	-0.8438	0.4465	0.6691
SO_RS18015	SO_3870	0.0000	1.0000	1.0000
SO_RS18025	SO_3872	1.5918	0.4413	0.6659
SO_RS18030	SO_3873	-3.2353	0.2210	0.4408
SO_RS18035	SO_3874	-8.6518	0.0078	0.0830
SO_RS18050		-7.8212	0.0402	0.1750
SO_RS18055	SO_3878	1.1219	0.4927	0.7066
SO_RS18060		3.8034	0.2885	0.5114
SO_RS18065	SO_3880	0.4345	0.8393	0.9785
SO_RS18070	SO_3882	-4.1898	0.1448	0.3437
SO_RS18075		0.0000	1.0000	1.0000
SO_RS18080	SO_3884	0.0000	1.0000	1.0000
SO_RS18085	SO_3885	-1.8106	0.3258	0.5549
SO_RS18090	SO_3886	8.9320	0.0565	0.2112
SO_RS18095	SO_3887	0.0000	1.0000	1.0000
SO_RS18100	SO_3888	-0.9489	0.3742	0.6039
SO_RS18110	SO_3890	1.3007	0.2527	0.4738
SO_RS18115	SO_3891	-2.7985	0.0776	0.2438
SO_RS18120	SO_3892	3.3777	0.0083	0.0855
SO_RS18125	SO_3894	-2.2051	0.2213	0.4410
SO_RS18130	SO_3895	-3.7925	0.0592	0.2170
SO_RS18135	SO_3896	2.5098	0.0000	0.0039

SO_RS06375	SO_1372	0.0000	1.0000	1.0000
SO_RS06380	SO_1373	0.0000	1.0000	1.0000
SO_RS06385	SO_1374	-4.8493	0.0267	0.1387
SO_RS06390	SO_1375	4.1291	0.0114	0.0981
SO_RS06395	SO_1376	1.1153	0.4891	0.7036
SO_RS06400	SO_1377	-0.9573	0.2930	0.5162
SO_RS06405	SO_1378	1.2751	0.4379	0.6639
SO_RS06410	SO_1379	8.3990	0.0291	0.1452
SO_RS06415	SO_1380	-0.9216	0.6114	0.8045
SO_RS06420	SO_1381	0.1511	0.8491	0.9864
SO_RS06425	SO_1382	-7.5059	0.0052	0.0673
SO_RS06430	SO_1383	-1.4666	0.4729	0.6904
SO_RS06435	SO_1385	7.3477	0.0297	0.1467
SO_RS06440	SO_1387	6.5677	0.0465	0.1884
SO_RS06445	SO_1388	2.8837	0.0776	0.2438
SO_RS06450	SO_1389	-0.7962	0.5106	0.7218
SO_RS06455	SO_1390	7.5464	0.0086	0.0868
SO_RS06460	SO_1391	-1.8662	0.1299	0.3219
SO_RS06465	SO_1392	-0.6655	0.5216	0.7309
SO_RS06470	SO_1393	2.0972	0.2814	0.5040
SO_RS06480	SO_1395	-8.0648	0.0166	0.1163
SO_RS06485	SO_1396	0.0000	1.0000	1.0000
SO_RS06490	SO_1397	7.6866	0.0069	0.0772
SO_RS06495	SO_1399	2.9042	0.3450	0.5726
SO_RS06500	SO_1400	-1.3751	0.4586	0.6787
SO_RS06505	SO_1401	-1.7150	0.1467	0.3454
SO_RS06510	SO_1402	-6.6150	0.0265	0.1382
SO_RS06515	SO_1403	2.4287	0.0623	0.2215
SO_RS06520	SO_1404	-5.0638	0.0546	0.2076
SO_RS06525	SO_1405	-3.2621	0.1123	0.2982
SO_RS06530	SO_1406	0.0000	1.0000	1.0000
SO_RS06535	SO_1407	0.0000	1.0000	1.0000
SO_RS06540	SO_1408	-0.3868	0.7060	0.8813
SO_RS06545	SO_1409	6.9687	0.0397	0.1739
SO_RS06550	SO_1410	2.3548	0.1537	0.3532
SO_RS06555	SO_1411	5.8172	0.0320	0.1516
SO_RS06560	SO_1412	5.1649	0.0251	0.1338
SO_RS06565	SO_1413	6.6778	0.0908	0.2656
SO_RS06570	SO_1414	1.1970	0.5603	0.7611
SO_RS06575	SO_1415	-6.7566	0.0067	0.0771
SO_RS06580	SO_1416	-6.8754	0.0034	0.0564
SO_RS06585	SO_1417	-3.8417	0.0398	0.1740
SO_RS06590	SO_1418	-1.2664	0.4645	0.6831
SO_RS06595	SO_1419	2.8178	0.1269	0.3181
SO_RS06600	SO_1420	4.1086	0.1802	0.3895
SO_RS06605	SO_1421	1.4541	0.3929	0.6218
SO_RS06610	SO_1422	-5.1167	0.0426	0.1786
SO_RS06615	SO_1424	-2.6939	0.1879	0.3999
SO_RS06620	SO_1425	-0.8530	0.4843	0.6997
SO_RS06625	SO_4834	0.0000	1.0000	1.0000
SO_RS06630	SO_1427	-2.4910	0.0085	0.0868
SO_RS06635	SO_1428	0.4953	0.4889	0.7036
SO_RS06640	SO_1429	1.6008	0.0575	0.2138
SO_RS06645	SO_1430	1.3151	0.0414	0.1767
SO_RS06650	SO_1431	-3.5341	0.0981	0.2771
SO_RS06655	SO_1432	-3.9305	0.0743	0.2406
SO_RS06660	SO_1434	1.7708	0.1775	0.3864
SO_RS06665		0.0000	1.0000	1.0000
SO_RS06670	SO_1436	7.5504	0.0951	0.2732
SO_RS06675	SO_1438	0.0000	1.0000	1.0000
SO_RS06680		1.8686	0.3014	0.5264
SO_RS06685	SO_1440	7.8496	0.0478	0.1913
SO_RS06690	SO_1441	-1.8790	0.0373	0.1670
SO_RS06695	SO_4821	0.0000	1.0000	1.0000
SO_RS06700	SO_1442	-4.6253	0.0701	0.2344
SO_RS06705	SO_1443	-6.6181	0.0253	0.1347
SO_RS06710	SO_1444	2.0860	0.4513	0.6716
SO_RS06715	SO_1445	0.0000	1.0000	1.0000

SO_RS18140	SO_3897	1.5531	0.0719	0.2373
SO_RS18145	SO_3898	-0.2158	0.8108	0.9603
SO_RS18150	SO_3899	0.8715	0.3291	0.5580
SO_RS18155	SO_3900	-7.3093	0.0200	0.1230
SO_RS18160	SO_3901	-0.2157	0.8854	1.0000
SO_RS18165	SO_3902	-2.6669	0.1662	0.3720
SO_RS18170	SO_3903	0.6121	0.6987	0.8756
SO_RS18175	SO_3904	1.7216	0.0122	0.1005
SO_RS18180	SO_3905	2.6107	0.0051	0.0670
SO_RS18185	SO_3906	0.3128	0.7040	0.8797
SO_RS18190	SO_3907	-0.1755	0.8791	1.0000
SO_RS18195	SO_3908	-1.7058	0.0953	0.2732
SO_RS18200	SO_3909	0.1609	0.9182	1.0000
SO_RS18205	SO_3910	2.4259	0.0164	0.1161
SO_RS18210	SO_3912	-3.4169	0.1063	0.2889
SO_RS18215	SO_3913	3.4289	0.2115	0.4297
SO_RS18220	SO_3914	-0.5183	0.7220	0.8923
SO_RS18225	SO_3915	2.3814	0.1278	0.3190
SO_RS18230	SO_3916	3.3585	0.0548	0.2076
SO_RS18235	SO_3917	-0.0399	0.9561	1.0000
SO_RS18240	SO_3918	4.0261	0.0298	0.1468
SO_RS18245	SO_3920	5.2077	0.1203	0.3106
SO_RS18250	SO_3921	0.0000	1.0000	1.0000
SO_RS18255	SO_3922	7.2414	0.0313	0.1497
SO_RS18260	SO_3923	-2.1842	0.2784	0.5009
SO_RS18265	SO_3924	0.0000	1.0000	1.0000
SO_RS18270	SO_3925	7.3582	0.0148	0.1100
SO_RS18275	SO_3926	-8.1855	0.0187	0.1205
SO_RS18280	SO_3927	3.7841	0.0007	0.0265
SO_RS18285	SO_3928	-1.1950	0.5011	0.7141
SO_RS18290	SO_3929	-0.2969	0.7848	0.9423
SO_RS18295	SO_3930	1.2558	0.0832	0.2536
SO_RS18300	SO_3931	0.2328	0.9170	1.0000
SO_RS18305	SO_3933	0.3953	0.8022	0.9538
SO_RS18310	SO_3934	3.2362	0.0117	0.0988
SO_RS18315	SO_3935	0.2152	0.6265	0.8182
SO_RS18320	SO_3936	0.5191	0.3228	0.5510
SO_RS18325	SO_3937	0.5468	0.3661	0.5954
SO_RS18330	SO_3938	2.0907	0.5201	0.7304
SO_RS18335	SO_3939	-0.7330	0.5046	0.7164
SO_RS18340	SO_3940	-0.8073	0.2643	0.4856
SO_RS18345	SO_3941	-0.6684	0.4695	0.6873
SO_RS18350	SO_3942	-0.0204	0.9722	1.0000
SO_RS18355	SO_3943	1.4830	0.3599	0.5888
SO_RS18365	SO_3945	1.4375	0.3889	0.6181
SO_RS18370	SO_3946	0.4994	0.7849	0.9423
SO_RS18375	SO_3948	-1.1904	0.0356	0.1613
SO_RS18380	SO_3949	-3.2346	0.1607	0.3646
SO_RS18385	SO_3950	5.4396	0.0701	0.2344
SO_RS18390	SO_3951	0.6576	0.1980	0.4103
SO_RS18395	SO_3952	-3.3818	0.0038	0.0583
SO_RS18400	SO_3953	-1.1385	0.0478	0.1913
SO_RS18405	SO_3954	-1.1921	0.2863	0.5093
SO_RS18410		2.0861	0.5144	0.7255
SO_RS18415	SO_3956	4.1465	0.0954	0.2732
SO_RS18420	SO_3957	-1.0485	0.5709	0.7692
SO_RS18425	SO_3958	-2.8879	0.1062	0.2889
SO_RS18430	SO_3959	-0.9761	0.4890	0.7036
SO_RS18435	SO_3960	-0.2623	0.8809	1.0000
SO_RS18440	SO_3961	-0.5096	0.6739	0.8565
SO_RS18445	SO_3962	-0.9630	0.5139	0.7251
SO_RS18450	SO_3963	0.6322	0.6542	0.8426
SO_RS18455	SO_3964	-2.0286	0.2553	0.4767
SO_RS18460	SO_3965	0.0000	1.0000	1.0000
SO_RS18465	SO_3966	3.0236	0.1083	0.2907
SO_RS18470	SO_3967	1.5543	0.3713	0.6010
SO_RS18475	SO_3969	4.3747	0.0265	0.1382
SO_RS18480	SO_3972	7.1643	0.0247	0.1330

SO_RS06720	SO_1447	-4.6883	0.1121	0.2978
SO_RS06725	SO_1448	-1.7454	0.2418	0.4625
SO_RS06730	SO_1450	-4.2905	0.1311	0.3240
SO_RS06735	SO_1451	0.0000	1.0000	1.0000
SO_RS06745	SO_1453	0.0000	1.0000	1.0000
SO_RS06750	SO_1454	0.0000	1.0000	1.0000
SO_RS06755	SO_1455	4.7545	0.1045	0.2867
SO_RS06765	SO_1457	-2.6277	0.0017	0.0420
SO_RS06770	SO_1458	-3.2459	0.0415	0.1767
SO_RS06775	SO_1459	0.8665	0.6657	0.8501
SO_RS06780	SO_1460	-1.5663	0.1205	0.3109
SO_RS06785	SO_1461	-0.7738	0.2643	0.4856
SO_RS06790	SO_1462	-0.5752	0.5033	0.7155
SO_RS06795	SO_1463	1.2490	0.1939	0.4062
SO_RS06805		-4.5918	0.0387	0.1712
SO_RS06810	SO_1467	1.5180	0.2854	0.5090
SO_RS06815	SO_1468	0.4036	0.5759	0.7721
SO_RS06820	SO_1469	7.8292	0.0700	0.2342
SO_RS06825	SO_4773	1.3003	0.5169	0.7277
SO_RS06830		-10.4526	0.0294	0.1457
SO_RS06835	SO_1470	1.2638	0.4729	0.6904
SO_RS06840	SO_1471	-0.5978	0.7348	0.9019
SO_RS06845	SO_m003	-2.4776	0.0000	0.0044
SO_RS06850	SO_1473	0.6956	0.5809	0.7774
SO_RS06855	SO_1474	1.9186	0.3820	0.6106
SO_RS06860	SO_1475	0.2232	0.8885	1.0000
SO_RS06865	SO_1476	-0.4812	0.7811	0.9414
SO_RS06870	SO_1478	0.4067	0.7316	0.9000
SO_RS06875	SO_1479	-1.2217	0.5611	0.7613
SO_RS06880	SO_1480	1.5862	0.2674	0.4893
SO_RS06885	SO_1481	-0.6292	0.6413	0.8323
SO_RS06890	SO_1482	-2.8643	0.0218	0.1279
SO_RS06895	SO_1483	3.5116	0.0004	0.0197
SO_RS06900	SO_1484	3.4184	0.0000	0.0052
SO_RS06905	SO_1486	8.2269	0.0109	0.0960
SO_RS06910	SO_1487	3.8034	0.2597	0.4814
SO_RS06915	SO_1489	3.5512	0.0224	0.1286
SO_RS06920	SO_1490	0.9714	0.0787	0.2453
SO_RS06925	SO_1492	0.0000	1.0000	1.0000
SO_RS06930	SO_1493	1.8345	0.3223	0.5504
SO_RS06935	SO_1494	0.0999	0.9455	1.0000
SO_RS06940	SO_1495	3.4289	0.1808	0.3900
SO_RS06945	SO_1496	-0.3374	0.8443	0.9827
SO_RS06950	SO_1498	-4.7790	0.0492	0.1938
SO_RS06955	SO_1499	-2.3110	0.2294	0.4506
SO_RS06960	SO_1500	-3.1306	0.0100	0.0932
SO_RS06965	SO_1501	1.7913	0.3617	0.5900
SO_RS06970	SO_1502	-1.6942	0.3482	0.5760
SO_RS06975	SO_1503	-2.9938	0.0650	0.2260
SO_RS06980	SO_1504	-1.2028	0.5432	0.7467
SO_RS06985	SO_1505	1.5076	0.2432	0.4643
SO_RS06990	SO_1506	2.1730	0.1176	0.3067
SO_RS06995	SO_1507	0.0393	0.9697	1.0000
SO_RS07000	SO_1508	-3.0780	0.1436	0.3429
SO_RS07010	SO_1510	-7.0801	0.0191	0.1206
SO_RS07015	SO_1511	8.7192	0.0614	0.2202
SO_RS07020	SO_1512	-0.4236	0.8067	0.9569
SO_RS07025	SO_1513	-0.5497	0.6747	0.8573
SO_RS07030		2.0861	0.5132	0.7244
SO_RS07050	SO_1518	2.4343	0.0002	0.0136
SO_RS07055	SO_1519	2.5257	0.0001	0.0103
SO_RS07060	SO_1520	1.8542	0.0012	0.0356
SO_RS07065	SO_1521	1.9452	0.0025	0.0490
SO_RS07070	SO_1522	1.7523	0.0120	0.0991
SO_RS07075	SO_1523	-0.5842	0.6580	0.8449
SO_RS07080	SO_1524	-0.7038	0.1665	0.3720
SO_RS07085	SO_1525	2.3753	0.0344	0.1580
SO_RS07090	SO_1526	0.2534	0.8422	0.9808

SO_RS18485	SO_3973	1.1686	0.0865	0.2589
SO_RS18490	SO_3974	7.2879	0.0562	0.2106
SO_RS18495	SO_3975	4.1994	0.1069	0.2894
SO_RS18500		0.0000	1.0000	1.0000
SO_RS18505		7.0779	0.0732	0.2405
SO_RS18510		4.1042	0.2769	0.4994
SO_RS18515	SO_3980	1.3355	0.1786	0.3876
SO_RS18520	SO_3981	-0.7405	0.6571	0.8442
SO_RS18525	SO_3982	7.1961	0.0286	0.1441
SO_RS18530	SO_3983	1.4269	0.4203	0.6489
SO_RS18535	SO_3984	0.9919	0.4459	0.6686
SO_RS18540	SO_3985	-2.0522	0.2632	0.4849
SO_RS18545	SO_3986	-1.7297	0.1903	0.4030
SO_RS18550	SO_3987	0.2225	0.8661	0.9992
SO_RS18555	SO_3988	-1.2453	0.0173	0.1182
SO_RS18560	SO_3990	0.7887	0.6491	0.8393
SO_RS18565	SO_3991	1.5531	0.2760	0.4983
SO_RS18570	SO_3993	-4.9289	0.0633	0.2231
SO_RS18575	SO_3994	-1.8653	0.0463	0.1878
SO_RS18580	SO_3996	0.0000	1.0000	1.0000
SO_RS18585	SO_3997	7.6414	0.0081	0.0846
SO_RS18590		2.4694	0.2366	0.4575
SO_RS18600		-0.2336	0.8860	1.0000
SO_RS18605	SO_4002	-0.2278	0.7400	0.9059
SO_RS18610	SO_4003	-2.2176	0.0569	0.2121
SO_RS18615	SO_4004	0.0000	1.0000	1.0000
SO_RS18620	SO_4005	-6.5332	0.0106	0.0948
SO_RS18625	SO_4006	0.9135	0.5854	0.7818
SO_RS18630	SO_4007	1.9252	0.4095	0.6381
SO_RS18635	SO_4008	-0.1810	0.7829	0.9418
SO_RS18640	SO_4011	7.7447	0.0487	0.1928
SO_RS18645	SO_4012	-2.0437	0.2963	0.5194
SO_RS18650	SO_4013	1.2170	0.4494	0.6703
SO_RS18655	SO_4014	-0.5177	0.4819	0.6978
SO_RS18660	SO_4015	4.7814	0.0337	0.1562
SO_RS18665	SO_4016	2.4908	0.1622	0.3672
SO_RS18670	SO_4017	-4.6471	0.0175	0.1182
SO_RS18675	SO_4018	-4.7268	0.0767	0.2434
SO_RS18680	SO_4019	4.5499	0.0970	0.2754
SO_RS18685	SO_4020	0.0992	0.9418	1.0000
SO_RS18690	SO_4021	5.6412	0.0416	0.1767
SO_RS18695	SO_4022	1.5167	0.2236	0.4437
SO_RS18705	SO_4025	-1.8977	0.0621	0.2215
SO_RS18710	SO_4027	0.0000	1.0000	1.0000
SO_RS18715	SO_4028	1.7154	0.1216	0.3123
SO_RS18720	SO_4029	-2.3022	0.0872	0.2600
SO_RS18725	SO_4030	-1.2819	0.0518	0.1989
SO_RS18730	SO_4031	3.4227	0.2879	0.5112
SO_RS18735	SO_4032	0.0000	1.0000	1.0000
SO_RS18740	SO_4034	-0.3923	0.4492	0.6703
SO_RS18745	SO_4035	6.1859	0.0348	0.1589
SO_RS18750	SO_4036	10.3262	0.0008	0.0269
SO_RS18755	SO_4037	4.1040	0.1889	0.4014
SO_RS18760	SO_4038	-1.1245	0.4872	0.7026
SO_RS18765	SO_4039	3.8326	0.0114	0.0981
SO_RS18770	SO_4040	-2.5367	0.0122	0.1005
SO_RS18775	SO_4042	-4.6679	0.0527	0.2020
SO_RS18780	SO_4043	9.5267	0.0006	0.0238
SO_RS18785	SO_4044	0.0000	1.0000	1.0000
SO_RS18790	SO_4045	0.0000	1.0000	1.0000
SO_RS18795	SO_4046	5.4288	0.1339	0.3279
SO_RS18800	SO_4047	1.8633	0.1138	0.3010
SO_RS18805	SO_4048	9.1556	0.0158	0.1135
SO_RS18810	SO_4050	5.3904	0.0220	0.1279
SO_RS18815	SO_4051	2.6450	0.0044	0.0621
SO_RS18820	SO_4052	1.6591	0.0273	0.1414
SO_RS18825	SO_4053	-0.5345	0.7004	0.8774
SO_RS18830	SO_4054	-2.9535	0.0041	0.0600

SO_RS07095	SO_1527	-6.0231	0.0413	0.1766
SO_RS07100	SO_1529	0.0898	0.7867	0.9428
SO_RS07105	SO_1530	0.4135	0.4975	0.7118
SO_RS07110	SO_1531	-0.4817	0.5988	0.7931
SO_RS07115	SO_1532	-0.5669	0.7246	0.8949
SO_RS07120	SO_1533	0.6709	0.3317	0.5598
SO_RS07125	SO_1534	-0.0384	0.9753	1.0000
SO_RS07130	SO_1535	3.4791	0.0682	0.2316
SO_RS07135	SO_1536	0.7699	0.4365	0.6631
SO_RS07140	SO_1538	1.8180	0.0028	0.0502
SO_RS07145	SO_1539	-0.4060	0.6393	0.8308
SO_RS07150		0.0000	1.0000	1.0000
SO_RS07155	SO_1541	0.0000	1.0000	1.0000
SO_RS07160	SO_1543	-2.0805	0.2635	0.4849
SO_RS07170	SO_1546	-6.9503	0.0037	0.0581
SO_RS07175	SO_1547	-1.9284	0.3784	0.6071
SO_RS07180	SO_1548	-2.1708	0.3395	0.5678
SO_RS07185	SO_1549	-2.3358	0.1499	0.3502
SO_RS07190	SO_1550	0.2798	0.6362	0.8281
SO_RS07195	SO_1551	0.7750	0.0968	0.2752
SO_RS07200	SO_1552	4.9135	0.1331	0.3266
SO_RS07210	SO_1556	-0.4357	0.6480	0.8389
SO_RS07215	SO_1557	6.9258	0.0180	0.1189
SO_RS07220	SO_1558	0.8666	0.3440	0.5721
SO_RS07225	SO_1559	2.8130	0.0927	0.2692
SO_RS07230	SO_1560	0.5523	0.3577	0.5874
SO_RS07235	SO_1561	-1.2038	0.1277	0.3190
SO_RS07240		0.0000	1.0000	1.0000
SO_RS07245	SO_1562	7.2665	0.0971	0.2754
SO_RS07250	SO_1563	7.0231	0.0001	0.0084
SO_RS07255	SO_1565	1.2483	0.3586	0.5882
SO_RS07260	SO_1566	4.0895	0.1240	0.3148
SO_RS07265	SO_1568	3.5517	0.0073	0.0791
SO_RS07270	SO_1569	-6.4663	0.0266	0.1385
SO_RS07275	SO_1570	-1.4075	0.4579	0.6784
SO_RS07280	SO_1571	0.2282	0.8024	0.9538
SO_RS07285	SO_1572	2.0861	0.4736	0.6905
SO_RS07290	SO_1575	7.9041	0.0135	0.1053
SO_RS07295	SO_1576	3.1516	0.0089	0.0873
SO_RS07300	SO_1577	1.8397	0.0097	0.0920
SO_RS07305	SO_1578	10.1810	0.0009	0.0304
SO_RS07310	SO_1579	2.0599	0.2007	0.4144
SO_RS07315	SO_1580	-2.2906	0.0925	0.2688
SO_RS07320	SO_1581	-2.2578	0.2492	0.4709
SO_RS07325	SO_1582	1.4915	0.1818	0.3912
SO_RS07330	SO_1583	0.5836	0.6350	0.8273
SO_RS07335	SO_1585	-6.8061	0.0213	0.1268
SO_RS07340	SO_1586	4.5754	0.1073	0.2903
SO_RS07345	SO_1587	0.0000	1.0000	1.0000
SO_RS07350	SO_1588	2.0860	0.4660	0.6843
SO_RS07355	SO_1589	-0.8715	0.3774	0.6068
SO_RS07360	SO_1590	1.7059	0.3039	0.5289
SO_RS07370		-7.6269	0.0188	0.1205
SO_RS07375	SO_1595	2.2912	0.1870	0.3989
SO_RS07380	SO_1597	7.6369	0.0073	0.0794
SO_RS07385	SO_1599	-1.0913	0.3213	0.5499
SO_RS07390	SO_1600	-4.3712	0.0712	0.2359
SO_RS07395	SO_1602	-0.3184	0.6042	0.7983
SO_RS07400	SO_1603	0.0000	1.0000	1.0000
SO_RS07405	SO_1604	-2.8450	0.0746	0.2410
SO_RS07410	SO_1605	-1.3823	0.4821	0.6978
SO_RS07415	SO_1606	1.1057	0.4816	0.6978
SO_RS07420	SO_1607	3.3386	0.0840	0.2544
SO_RS07425	SO_1608	0.8030	0.5345	0.7396
SO_RS07430	SO_1609	-1.7655	0.2672	0.4891
SO_RS07435	SO_1610	-2.4152	0.1263	0.3176
SO_RS07440	SO_1611	-1.0602	0.5960	0.7916
SO_RS07445	SO_1612	-2.6629	0.0233	0.1302

SO_RS18835	SO_4055	-1.6918	0.1839	0.3946
SO_RS18840	SO_4056	-4.5249	0.0023	0.0465
SO_RS18845	SO_4057	-0.5276	0.4886	0.7036
SO_RS18850	SO_4058	0.0000	1.0000	1.0000
SO_RS18855	SO_4060	13.7575	0.0000	0.0012
SO_RS18860	SO_4061	2.9317	0.0891	0.2632
SO_RS18865	SO_4062	1.8651	0.0123	0.1005
SO_RS18870	SO_4064	0.0016	0.9991	1.0000
SO_RS18875	SO_4066	4.4141	0.0158	0.1136
SO_RS18885	SO_4068	5.9018	0.0475	0.1910
SO_RS18890	SO_4069	-2.5826	0.1938	0.4062
SO_RS18895	SO_4070	0.1700	0.7637	0.9263
SO_RS18900	SO_4072	-1.3411	0.4335	0.6602
SO_RS18905	SO_4073	-5.4517	0.0760	0.2422
SO_RS18910	SO_4075	-2.7130	0.1518	0.3524
SO_RS18915	SO_4077	1.7289	0.2176	0.4380
SO_RS18920	SO_4078	0.1989	0.7642	0.9263
SO_RS18925	SO_4079	1.3367	0.0850	0.2559
SO_RS18930	SO_4080	-0.2500	0.8454	0.9836
SO_RS18935	SO_4081	8.4552	0.0230	0.1295
SO_RS18945	SO_4087	4.6306	0.0497	0.1948
SO_RS18950	SO_4088	-4.4807	0.0325	0.1527
SO_RS18955	SO_4089	4.2781	0.0009	0.0290
SO_RS18960	SO_4090	2.0552	0.0098	0.0920
SO_RS18965	SO_4091	-0.4934	0.7437	0.9084
SO_RS18970	SO_4092	-0.6713	0.7186	0.8907
SO_RS18975	SO_4093	-1.3485	0.4432	0.6665
SO_RS18980	SO_4094	0.5004	0.7569	0.9209
SO_RS18985	SO_4095	4.5872	0.0130	0.1035
SO_RS18990	SO_4096	7.5977	0.0237	0.1304
SO_RS18995	SO_4097	-0.3047	0.8166	0.9637
SO_RS19000	SO_4098	-0.9954	0.0565	0.2112
SO_RS19005	SO_4100	-0.5959	0.4244	0.6532
SO_RS19010	SO_4101	4.9193	0.0682	0.2316
SO_RS19015	SO_4102	1.4871	0.3413	0.5695
SO_RS19020	SO_4103	-0.1900	0.9116	1.0000
SO_RS19025	SO_4104	3.4321	0.2532	0.4741
SO_RS19030	SO_4105	1.2716	0.1486	0.3477
SO_RS19035	SO_4106	1.2336	0.1048	0.2871
SO_RS19040	SO_4107	-1.6375	0.2652	0.4863
SO_RS19045	SO_4108	1.0027	0.2547	0.4757
SO_RS19050	SO_4109	0.6909	0.3751	0.6045
SO_RS19055	SO_4110	-2.9483	0.1728	0.3802
SO_RS19060	SO_4111	-0.8921	0.4162	0.6445
SO_RS19065	SO_4112	-0.8110	0.4161	0.6445
SO_RS19070	SO_4113	0.0000	1.0000	1.0000
SO_RS19075	SO_4114	-1.9615	0.2410	0.4613
SO_RS19080	SO_4809	1.0341	0.5328	0.7386
SO_RS19085	SO_4115	-2.0982	0.0297	0.1467
SO_RS19090	SO_4116	-0.1371	0.9089	1.0000
SO_RS19095	SO_4118	2.9374	0.0838	0.2543
SO_RS19100	SO_4120	2.9042	0.3133	0.5403
SO_RS19105	SO_4121	-0.0127	0.9904	1.0000
SO_RS19110	SO_4122	-0.6760	0.4050	0.6347
SO_RS19115	SO_4123	-0.1395	0.9059	1.0000
SO_RS19120	SO_4124	-6.5882	0.0239	0.1305
SO_RS19125	SO_4126	0.3222	0.8502	0.9872
SO_RS19130	SO_4128	-0.8210	0.3548	0.5835
SO_RS19135	SO_4129	-2.3939	0.1386	0.3348
SO_RS19140	SO_4130	-1.6722	0.1765	0.3852
SO_RS19145	SO_4131	2.0768	0.1238	0.3147
SO_RS19150	SO_4133	0.1713	0.7444	0.9090
SO_RS19155	SO_4134	-0.0155	0.9878	1.0000
SO_RS19160	SO_4135	5.5130	0.0103	0.0943
SO_RS19165	SO_4136	-1.4107	0.3445	0.5724
SO_RS19170	SO_4138	-1.0013	0.6159	0.8083
SO_RS19175	SO_4139	-0.8099	0.4384	0.6639
SO_RS19180	SO_4140	4.5393	0.0211	0.1268

SO_RS07450	SO_1613	2.0859	0.4304	0.6589
SO_RS07455	SO_1614	2.1743	0.0689	0.2324
SO_RS07460	SO_1615	4.3647	0.1816	0.3909
SO_RS07465	SO_1616	-1.3843	0.3924	0.6214
SO_RS07470	SO_1617	-7.0607	0.0187	0.1205
SO_RS07475	SO_1618	1.7676	0.3089	0.5354
SO_RS07480	SO_1619	3.4228	0.2637	0.4849
SO_RS07485	SO_1620	7.4447	0.0213	0.1268
SO_RS07490	SO_1622	-1.4593	0.4091	0.6376
SO_RS07495	SO_1623	-2.9088	0.1743	0.3824
SO_RS07500	SO_1624	0.7282	0.6601	0.8461
SO_RS07505	SO_1625	-0.7667	0.4509	0.6712
SO_RS07510	SO_1626	-0.4199	0.7460	0.9107
SO_RS07515	SO_1627	-0.3333	0.7719	0.9324
SO_RS07520	SO_1629	1.3413	0.0053	0.0675
SO_RS07525	SO_1630	1.2772	0.0285	0.1436
SO_RS07530	SO_1631	-1.5632	0.2763	0.4986
SO_RS07535	SO_1632	2.2466	0.0585	0.2159
SO_RS07540	SO_1633	1.7100	0.2874	0.5105
SO_RS07545	SO_1634	-0.8860	0.4237	0.6524
SO_RS07550	SO_1635	-2.3083	0.0209	0.1265
SO_RS07555	SO_1636	0.4306	0.6482	0.8389
SO_RS07560	SO_1637	-2.0414	0.0030	0.0531
SO_RS07565	SO_1638	-0.7817	0.2484	0.4706
SO_RS07570	SO_1639	-0.9875	0.0899	0.2640
SO_RS07575	SO_1640	1.7178	0.2940	0.5170
SO_RS07580	SO_1641	-1.4454	0.0347	0.1585
SO_RS07585	SO_1642	-0.4292	0.7675	0.9287
SO_RS07590	SO_1643	-3.1714	0.2052	0.4211
SO_RS07595	SO_1644	-0.9629	0.0992	0.2790
SO_RS07600	SO_1645	0.2716	0.7757	0.9363
SO_RS07605	SO_1646	2.9357	0.0038	0.0583
SO_RS07610	SO_1647	-1.7803	0.2225	0.4427
SO_RS07615	SO_1648	2.5532	0.0760	0.2422
SO_RS07620	SO_1650	0.7618	0.6834	0.8645
SO_RS07625	SO_1651	-1.3143	0.0822	0.2514
SO_RS07630	SO_1652	-1.6145	0.0934	0.2701
SO_RS07635	SO_1653	0.4543	0.7532	0.9171
SO_RS07640	SO_1655	3.9485	0.0007	0.0265
SO_RS07645	SO_1656	1.9766	0.0085	0.0868
SO_RS07650	SO_1657	0.7444	0.6767	0.8587
SO_RS07655	SO_1658	-0.0056	0.9972	1.0000
SO_RS07660	SO_1659	2.2583	0.0005	0.0214
SO_RS07665	SO_1661	-0.6214	0.6524	0.8409
SO_RS07670	SO_1662	0.0000	1.0000	1.0000
SO_RS07675	SO_1663	0.0000	1.0000	1.0000
SO_RS07680	SO_1664	-0.2080	0.7342	0.9014
SO_RS07685	SO_1665	0.4684	0.4276	0.6562
SO_RS07690	SO_1666	3.5189	0.0634	0.2231
SO_RS07695	SO_1667	-1.3332	0.4647	0.6831
SO_RS07700	SO_1669	0.6356	0.7169	0.8899
SO_RS07705	SO_1670	1.5185	0.0274	0.1415
SO_RS07710	SO_1671	-3.8495	0.0049	0.0661
SO_RS07715	SO_1672	-0.7618	0.6497	0.8393
SO_RS07720	SO_1673	3.3514	0.0001	0.0095
SO_RS07725	SO_1674	2.9816	0.0433	0.1797
SO_RS07730	SO_1675	-1.5796	0.0232	0.1302
SO_RS07735	SO_1676	-2.0272	0.0593	0.2170
SO_RS07740	SO_1677	0.4020	0.4718	0.6896
SO_RS07745	SO_1678	-0.5402	0.3824	0.6109
SO_RS07750	SO_1679	-1.3681	0.1030	0.2844
SO_RS07755	SO_1680	-0.5845	0.4223	0.6510
SO_RS07760	SO_1681	0.3339	0.4216	0.6501
SO_RS07765	SO_1682	2.2927	0.0012	0.0348
SO_RS07770	SO_1683	-0.6736	0.2291	0.4506
SO_RS07775	SO_1685	7.4026	0.0847	0.2558
SO_RS07780	SO_1686	-0.7982	0.5682	0.7672
SO_RS07785	SO_1687	-3.8644	0.0408	0.1758

SO_RS19185	SO_4141	12.2985	0.0000	0.0052
SO_RS19190	SO_4142	0.0000	1.0000	1.0000
SO_RS19195	SO_4143	-9.8416	0.0182	0.1199
SO_RS19200	SO_4144	8.0401	0.0359	0.1619
SO_RS19205	SO_4145	-5.1695	0.0245	0.1324
SO_RS19210	SO_4146	-8.2539	0.0171	0.1175
SO_RS19215	SO_4147	-0.5215	0.7914	0.9462
SO_RS19220	SO_4148	-0.4805	0.6103	0.8037
SO_RS19225	SO_4149	-0.5055	0.5166	0.7275
SO_RS19230	SO_4150	3.4655	0.1288	0.3203
SO_RS19235	SO_4151	4.6937	0.0130	0.1037
SO_RS19240	SO_4152	3.8033	0.2192	0.4393
SO_RS19245	SO_4153	8.2089	0.0087	0.0869
SO_RS19250	SO_4154	-0.1637	0.9137	1.0000
SO_RS19255	SO_4155	-0.7628	0.5224	0.7313
SO_RS19260	SO_4157	4.5956	0.0244	0.1324
SO_RS19265	SO_4159	1.5610	0.0582	0.2154
SO_RS19270	SO_4160	3.9934	0.0349	0.1589
SO_RS19275	SO_4162	1.5215	0.1920	0.4046
SO_RS19280	SO_4163	0.9245	0.0450	0.1849
SO_RS19285	SO_4164	3.2163	0.0994	0.2791
SO_RS19290		-7.7885	0.0178	0.1187
SO_RS19295	SO_4165	9.6610	0.1062	0.2889
SO_RS19300		0.2242	0.8726	1.0000
SO_RS19310	SO_4168	0.0000	1.0000	1.0000
SO_RS19315	SO_4169	0.5782	0.7084	0.8826
SO_RS19320	SO_4170	7.9488	0.0138	0.1067
SO_RS19325	SO_4171	8.3684	0.0059	0.0720
SO_RS19330	SO_4172	-5.0638	0.0438	0.1814
SO_RS19335	SO_4173	-0.8623	0.5975	0.7927
SO_RS19340	SO_4174	0.4929	0.7397	0.9059
SO_RS19345	SO_4176	-3.2362	0.0975	0.2762
SO_RS19350	SO_4177	1.3770	0.4325	0.6598
SO_RS19355	SO_4178	-2.6056	0.0785	0.2453
SO_RS19360	SO_4179	0.3828	0.8067	0.9569
SO_RS19365	SO_4180	-2.9501	0.0249	0.1336
SO_RS19370	SO_4181	2.1851	0.1876	0.3999
SO_RS19375	SO_4182	1.1688	0.5206	0.7304
SO_RS19385		0.0000	1.0000	1.0000
SO_RS19390	SO_4187	2.2949	0.0966	0.2751
SO_RS19395	SO_4189	8.3851	0.0155	0.1122
SO_RS19400	SO_4190	0.6665	0.2403	0.4612
SO_RS19405	SO_4191	-3.4072	0.0947	0.2725
SO_RS19410	SO_4193	-3.3079	0.1762	0.3848
SO_RS19415	SO_4194	-2.6177	0.2714	0.4932
SO_RS19420	SO_4195	0.0000	1.0000	1.0000
SO_RS19425	SO_4196	1.4114	0.5128	0.7242
SO_RS19430	SO_4197	6.3053	0.0000	0.0022
SO_RS19435	SO_4198	4.1352	0.0206	0.1257
SO_RS19440	SO_4199	-0.5742	0.4730	0.6904
SO_RS19445	SO_4200	-0.6253	0.7890	0.9445
SO_RS19450	SO_4201	-1.2929	0.4490	0.6703
SO_RS19455	SO_4202	-0.7452	0.5590	0.7605
SO_RS19460	SO_4203	-3.7691	0.0017	0.0421
SO_RS19465	SO_4204	-4.0027	0.0276	0.1423
SO_RS19470	SO_4205	-3.8208	0.0638	0.2235
SO_RS19475	SO_4206	0.0000	1.0000	1.0000
SO_RS19480	SO_4207	4.5773	0.1625	0.3675
SO_RS19485	SO_4208	1.8662	0.2188	0.4386
SO_RS19490	SO_4210	0.0000	1.0000	1.0000
SO_RS19500	SO_r017	0.0000	1.0000	1.0000
SO_RS19520	SO_4211	-0.4238	0.4113	0.6399
SO_RS19525	SO_4212	-0.8570	0.3625	0.5909
SO_RS19530	SO_4213	1.2072	0.4276	0.6562
SO_RS19535	SO_4214	0.8120	0.0416	0.1767
SO_RS19540	SO_4215	1.8105	0.0014	0.0379
SO_RS19545	SO_4216	-0.5860	0.3917	0.6204
SO_RS19550	SO_4217	-0.7906	0.2937	0.5169

SO_RS07790	SO_1689	3.3118	0.0013	0.0359
SO_RS07795	SO_1690	0.1927	0.7643	0.9263
SO_RS07800	SO_1691	3.3770	0.0185	0.1205
SO_RS07805	SO_1692	-1.6201	0.4265	0.6557
SO_RS07810	SO_1694	-3.0143	0.0980	0.2769
SO_RS07815	SO_1695	2.9117	0.4148	0.6429
SO_RS07820	SO_1697	1.6145	0.3369	0.5647
SO_RS07825	SO_1698	9.7775	0.0035	0.0572
SO_RS07830	SO_1699	-2.2841	0.2114	0.4296
SO_RS07835	SO_1700	1.9155	0.0810	0.2489
SO_RS07840	SO_1701	-1.2114	0.5016	0.7144
SO_RS07845	SO_1702	3.4289	0.2003	0.4138
SO_RS07850	SO_1703	7.0779	0.0691	0.2327
SO_RS07855	SO_1705	1.5743	0.1270	0.3181
SO_RS07860	SO_1706	3.1186	0.0797	0.2469
SO_RS07865	SO_1707	2.5018	0.0308	0.1489
SO_RS07870	SO_1708	3.1524	0.0642	0.2247
SO_RS07875	SO_1709	-3.1923	0.1642	0.3693
SO_RS07880	SO_1711	-0.8943	0.6057	0.7990
SO_RS07885	SO_1712	1.5932	0.3816	0.6106
SO_RS07890	SO_1713	4.7545	0.0849	0.2558
SO_RS07895	SO_1715	-1.9028	0.2789	0.5012
SO_RS07900	SO_1716	-8.7266	0.0163	0.1155
SO_RS07905	SO_1717	3.6083	0.0104	0.0944
SO_RS07910	SO_1718	-0.4584	0.7918	0.9462
SO_RS07915	SO_1720	0.2240	0.8676	0.9998
SO_RS07920	SO_1722	4.9007	0.0092	0.0891
SO_RS07925	SO_1723	0.7312	0.4206	0.6492
SO_RS07930	SO_1724	-0.1423	0.8038	0.9553
SO_RS07935	SO_1725	-0.5287	0.5723	0.7693
SO_RS07940	SO_1726	2.7312	0.0212	0.1268
SO_RS07945	SO_1727	3.2244	0.0551	0.2079
SO_RS07950	SO_1728	0.0000	1.0000	1.0000
SO_RS07960	SO_1732	-3.1736	0.2151	0.4338
SO_RS07965	SO_1733	-4.5973	0.1139	0.3012
SO_RS07970	SO_1734	-0.7888	0.5675	0.7666
SO_RS07975	SO_1736	-1.7901	0.3125	0.5397
SO_RS07980	SO_1738	-0.5689	0.6269	0.8182
SO_RS07985	SO_1739	2.9080	0.4120	0.6405
SO_RS07990	SO_1741	1.5564	0.4530	0.6730
SO_RS07995	SO_1742	0.0202	0.9877	1.0000
SO_RS08000	SO_1743	0.4429	0.8126	0.9618
SO_RS08005	SO_1744	-0.9134	0.6649	0.8495
SO_RS08010	SO_1745	0.0733	0.9534	1.0000
SO_RS08015	SO_1746	-1.1443	0.6181	0.8100
SO_RS08020	SO_1747	0.6797	0.2319	0.4531
SO_RS08025	SO_1748	0.4486	0.8262	0.9683
SO_RS08030	SO_1750	3.8959	0.0227	0.1291
SO_RS08035	SO_1751	-2.3568	0.0129	0.1035
SO_RS08040	SO_1753	4.9818	0.0106	0.0948
SO_RS08045	SO_1755	2.7824	0.0232	0.1302
SO_RS08050	SO_1756	0.8730	0.5270	0.7334
SO_RS08055	SO_1757	5.3208	0.0671	0.2295
SO_RS08060	SO_1758	-1.6705	0.3383	0.5663
SO_RS08065	SO_1759	0.0000	1.0000	1.0000
SO_RS08070	SO_1760	-1.4811	0.5397	0.7437
SO_RS08075	SO_1762	5.2078	0.1232	0.3141
SO_RS08080	SO_1763	1.8089	0.2431	0.4643
SO_RS08085	SO_1764	0.0000	1.0000	1.0000
SO_RS08090	SO_1765	3.8034	0.3061	0.5318
SO_RS08095		-3.3986	0.0215	0.1274
SO_RS08100	SO_1767	-0.4717	0.8254	0.9683
SO_RS08105		-5.2793	0.0161	0.1148
SO_RS08110	SO_1769	-1.3282	0.2506	0.4717
SO_RS08115	SO_1770	6.0531	0.0897	0.2640
SO_RS08120	SO_1771	1.1050	0.4884	0.7036
SO_RS08125	SO_1773	2.3262	0.0051	0.0670
SO_RS08130	SO_1774	8.1625	0.0059	0.0718

SO_RS19555	SO_4218	-0.2368	0.6850	0.8651
SO_RS19560	SO_4219	-0.1290	0.9240	1.0000
SO_RS19565	SO_4220	-0.2535	0.8750	1.0000
SO_RS19570	SO_4221	-0.1050	0.8701	1.0000
SO_RS19575	SO_4222	-0.7407	0.4187	0.6478
SO_RS19580	SO_4223	-0.0250	0.9839	1.0000
SO_RS19585	SO_4224	-2.5444	0.0029	0.0515
SO_RS19590	SO_4225	-0.1033	0.9201	1.0000
SO_RS19595	SO_4226	-3.3369	0.1823	0.3919
SO_RS19600	SO_4227	-0.0637	0.9418	1.0000
SO_RS19605	SO_4228	1.7003	0.2350	0.4559
SO_RS19610	SO_4229	-1.3421	0.0623	0.2215
SO_RS19615	SO_4230	0.1686	0.8975	1.0000
SO_RS19620	SO_4231	2.4964	0.0900	0.2640
SO_RS19625	SO_4232	0.5869	0.6502	0.8393
SO_RS19630	SO_4233	9.2181	0.0033	0.0556
SO_RS19635	SO_4234	2.8722	0.0238	0.1304
SO_RS19640	SO_4235	-0.2633	0.7534	0.9171
SO_RS19645	SO_4236	1.3276	0.1940	0.4062
SO_RS19650		-6.4606	0.0326	0.1530
SO_RS19660		-5.8358	0.0331	0.1541
SO_RS19665	SO_4241	-2.9164	0.1405	0.3381
SO_RS19670	SO_4242	-2.2959	0.0889	0.2631
SO_RS19675	SO_4243	0.1876	0.9172	1.0000
SO_RS19680	SO_4244	8.3273	0.0422	0.1780
SO_RS19685	SO_4245	-1.9236	0.3353	0.5637
SO_RS19690	SO_4246	-1.5670	0.4058	0.6353
SO_RS19695	SO_4247	-0.9975	0.4863	0.7018
SO_RS19700	SO_4248	-2.1449	0.1468	0.3455
SO_RS19705	SO_4249	2.5144	0.1180	0.3073
SO_RS19710	SO_4250	-2.2105	0.2870	0.5102
SO_RS19715	SO_4251	4.9267	0.1553	0.3560
SO_RS19720	SO_4252	-1.0610	0.4122	0.6405
SO_RS19725	SO_4254	4.2726	0.0158	0.1135
SO_RS19730	SO_4255	0.0693	0.9576	1.0000
SO_RS19735	SO_4256	1.2044	0.2119	0.4300
SO_RS19740	SO_4257	1.0477	0.5271	0.7334
SO_RS19745	SO_4258	-1.0132	0.1049	0.2871
SO_RS19750	SO_4259	1.9903	0.2562	0.4777
SO_RS19755	SO_4260	0.0000	1.0000	1.0000
SO_RS19760	SO_4261	5.2238	0.0179	0.1189
SO_RS19765	SO_4262	-1.9809	0.3369	0.5647
SO_RS19770	SO_4263	0.5894	0.6597	0.8461
SO_RS19775	SO_4264	-2.6361	0.0074	0.0805
SO_RS19780	SO_4265	7.3195	0.0118	0.0990
SO_RS19785	SO_4266	8.8597	0.0033	0.0554
SO_RS19790	SO_4267	0.4488	0.8058	0.9566
SO_RS19800	SO_4270	-0.6703	0.7312	0.8999
SO_RS19810	SO_4272	0.0000	1.0000	1.0000
SO_RS19815		-4.6770	0.0776	0.2438
SO_RS19820	SO_4274	5.7273	0.0344	0.1580
SO_RS19825	SO_4825	0.0000	1.0000	1.0000
SO_RS19835	SO_4279	-0.6332	0.6347	0.8273
SO_RS19840	SO_4280	1.8645	0.0226	0.1291
SO_RS19845	SO_4281	1.7144	0.3031	0.5280
SO_RS19850	SO_4282	6.5693	0.0265	0.1382
SO_RS19855	SO_4283	0.4296	0.8398	0.9788
SO_RS19860	SO_4284	0.0000	1.0000	1.0000
SO_RS19870	SO_4286	-0.8253	0.3501	0.5780
SO_RS19875	SO_4287	-1.0674	0.2409	0.4613
SO_RS19880	SO_4288	-0.9900	0.4443	0.6674
SO_RS19885	SO_4289	5.2003	0.0506	0.1962
SO_RS19890	SO_4290	-2.5727	0.0680	0.2315
SO_RS19895	SO_4291	0.6890	0.6830	0.8642
SO_RS19900	SO_4292	0.9031	0.5318	0.7378
SO_RS19910	SO_4295	3.8136	0.2337	0.4540
SO_RS19915	SO_4296	2.0860	0.4475	0.6693
SO_RS19920	SO_4297	0.0000	1.0000	1.0000

SO_RS08135	SO_1775	4.3608	0.1015	0.2828
SO_RS08140	SO_1776	2.2819	0.0001	0.0084
SO_RS08145	SO_1777	0.4225	0.3427	0.5706
SO_RS08150	SO_1778	1.4410	0.0051	0.0670
SO_RS08155	SO_1779	1.6204	0.0009	0.0304
SO_RS08160	SO_1780	-1.4691	0.2735	0.4957
SO_RS08165	SO_1781	-2.7083	0.1804	0.3895
SO_RS08170	SO_1782	5.2077	0.1229	0.3141
SO_RS08175	SO_1783	4.3528	0.1723	0.3802
SO_RS08180	SO_1784	-1.0001	0.0284	0.1436
SO_RS08185	SO_1786	2.0824	0.0321	0.1518
SO_RS08190	SO_1787	0.6472	0.1271	0.3183
SO_RS08195	SO_1788	-1.0933	0.5166	0.7275
SO_RS08200	SO_1789	7.9048	0.0184	0.1203
SO_RS08205	SO_1790	-1.6888	0.2528	0.4738
SO_RS08210	SO_1791	0.7126	0.2381	0.4588
SO_RS08215	SO_1792	2.4970	0.0229	0.1291
SO_RS08235	SO_1793	0.7231	0.1134	0.3007
SO_RS08240	SO_1794	0.2739	0.5824	0.7789
SO_RS08245	SO_1795	0.4064	0.4308	0.6589
SO_RS08250	SO_1796	0.9005	0.0118	0.0989
SO_RS08255	SO_1797	5.3155	0.0001	0.0084
SO_RS08260	SO_1798	0.2120	0.7108	0.8845
SO_RS08265	SO_1799	-2.1140	0.3013	0.5264
SO_RS08270	SO_1800	1.2920	0.0702	0.2345
SO_RS08275	SO_1801	-1.2552	0.3673	0.5967
SO_RS08280	SO_1802	-3.6119	0.0052	0.0673
SO_RS08285	SO_1803	-0.7907	0.6602	0.8461
SO_RS08290	SO_1804	-2.7785	0.0507	0.1964
SO_RS08295	SO_1805	0.2378	0.7379	0.9050
SO_RS08300	SO_1806	-1.0011	0.4997	0.7135
SO_RS08305	SO_1807	0.5035	0.5248	0.7328
SO_RS08310	SO_1808	0.7187	0.5613	0.7613
SO_RS08315	SO_1809	0.2586	0.6163	0.8083
SO_RS08320	SO_1810	1.1453	0.2774	0.5000
SO_RS08325	SO_1811	2.6895	0.0638	0.2235
SO_RS08330	SO_1812	3.6409	0.0400	0.1741
SO_RS08335	SO_1813	5.4386	0.0457	0.1868
SO_RS08340	SO_m005	-3.2353	0.2294	0.4506
SO_RS08345	SO_1814	0.3196	0.8558	0.9913
SO_RS08350	SO_1815	-2.7020	0.1615	0.3660
SO_RS08355	SO_1816	3.0019	0.0586	0.2160
SO_RS08360	SO_1817	5.4419	0.0788	0.2453
SO_RS08365	SO_1818	5.3161	0.1149	0.3025
SO_RS08370	SO_1819	-0.5254	0.5724	0.7693
SO_RS08375	SO_1820	-0.0689	0.9448	1.0000
SO_RS08380	SO_1821	0.3514	0.6708	0.8548
SO_RS08385	SO_1822	1.3467	0.3760	0.6056
SO_RS08390	SO_1824	0.7290	0.0715	0.2363
SO_RS08395	SO_1825	0.7652	0.0310	0.1492
SO_RS08400	SO_1826	1.4295	0.0241	0.1310
SO_RS08405	SO_1827	-0.7189	0.2439	0.4647
SO_RS08410	SO_1828	-0.1288	0.7410	0.9064
SO_RS08415	SO_1829	0.5767	0.0809	0.2488
SO_RS08420	SO_1830	8.0898	0.0211	0.1268
SO_RS08425	SO_1831	2.3821	0.0257	0.1362
SO_RS08430	SO_1832	0.6490	0.7031	0.8793
SO_RS08440	SO_1834	-5.6233	0.0618	0.2207
SO_RS08445	SO_1835	0.0000	1.0000	1.0000
SO_RS08450	SO_1836	1.1756	0.4085	0.6371
SO_RS08455	SO_1839	-0.5560	0.6796	0.8616
SO_RS08460	SO_1840	-1.5666	0.0712	0.2359
SO_RS08465	SO_1841	-0.4561	0.8315	0.9726
SO_RS08470	SO_1843	4.3646	0.1930	0.4055
SO_RS08475	SO_1844	0.7724	0.6369	0.8285
SO_RS08480	SO_1845	5.4385	0.0517	0.1988
SO_RS08485	SO_1846	0.6028	0.7326	0.9003
SO_RS08490	SO_1848	-1.5920	0.1740	0.3824
SO_RS19925	SO_4298	4.8578	0.0127	0.1031
SO_RS19930	SO_4299	-1.4314	0.3018	0.5264
SO_RS19940	SO_4302	-7.6895	0.0185	0.1205
SO_RS19945	SO_4303	4.0979	0.0166	0.1163
SO_RS19950	SO_4304	-1.3738	0.4336	0.6602
SO_RS19955	SO_4305	1.2910	0.4798	0.6966
SO_RS19960	SO_4306	0.2725	0.7835	0.9418
SO_RS19965	SO_4307	-1.2686	0.4722	0.6900
SO_RS19970	SO_4308	-0.5079	0.5235	0.7321
SO_RS19975	SO_4309	0.8792	0.5672	0.7664
SO_RS19980	SO_4310	0.0000	1.0000	1.0000
SO_RS19985	SO_4311	-3.1586	0.1681	0.3741
SO_RS19990	SO_4312	0.3727	0.6153	0.8080
SO_RS19995	SO_4313	0.2344	0.8403	0.9788
SO_RS20000	SO_4314	7.2314	0.0125	0.1021
SO_RS20005	SO_4315	-0.7833	0.5044	0.7164
SO_RS20010	SO_4316	-2.1613	0.1329	0.3263
SO_RS20040	SO_4318	-0.7699	0.2438	0.4646
SO_RS20045	SO_4319	-0.7622	0.5617	0.7615
SO_RS20050	SO_4320	1.8633	0.0015	0.0386
SO_RS20055	SO_4321	1.2164	0.0885	0.2625
SO_RS20060	SO_4322	1.0301	0.2797	0.5023
SO_RS20065	SO_4323	-0.4787	0.5040	0.7161
SO_RS20090	SO_4324	-0.0859	0.9346	1.0000
SO_RS20095	SO_4325	-0.9683	0.3035	0.5284
SO_RS20100	SO_4326	-0.4080	0.6255	0.8172
SO_RS20105	SO_4327	0.8197	0.1919	0.4046
SO_RS20110	SO_4328	2.4393	0.0272	0.1411
SO_RS20115	SO_4329	4.7593	0.0799	0.2470
SO_RS20120	SO_4330	0.0000	1.0000	1.0000
SO_RS20125	SO_4331	4.7608	0.1265	0.3176
SO_RS20130	SO_4332	0.9191	0.5504	0.7527
SO_RS20135	SO_4333	3.4229	0.3460	0.5736
SO_RS20140	SO_4334	-4.8462	0.0546	0.2076
SO_RS20145	SO_4335	0.0000	1.0000	1.0000
SO_RS20150	SO_4337	7.2614	0.0129	0.1035
SO_RS20155	SO_4339	0.0000	1.0000	1.0000
SO_RS20160	SO_4340	2.3236	0.1760	0.3845
SO_RS20165	SO_4341	-1.4278	0.2367	0.4575
SO_RS20170	SO_4342	0.0000	1.0000	1.0000
SO_RS20175	SO_4343	-1.4276	0.1352	0.3304
SO_RS20180	SO_4344	-0.7923	0.2432	0.4643
SO_RS20185	SO_4345	2.8092	0.0004	0.0187
SO_RS20190	SO_4346	-0.4424	0.7957	0.9485
SO_RS20195	SO_4347	0.8858	0.1487	0.3479
SO_RS20200	SO_4349	-0.2684	0.7847	0.9423
SO_RS20205	SO_4350	-3.2761	0.1453	0.3437
SO_RS20210	SO_4351	0.2986	0.7939	0.9474
SO_RS20220	SO_4354	2.0860	0.4298	0.6583
SO_RS20225	SO_4355	0.0000	1.0000	1.0000
SO_RS20230	SO_4356	-1.6709	0.3518	0.5802
SO_RS20235	SO_4357	3.3977	0.1035	0.2849
SO_RS20240	SO_4358	-0.0893	0.9522	1.0000
SO_RS20245	SO_4359	-7.8949	0.0029	0.0513
SO_RS20250	SO_4360	0.0000	1.0000	1.0000
SO_RS20255	SO_4361	0.0000	1.0000	1.0000
SO_RS20260	SO_4362	0.0000	1.0000	1.0000
SO_RS20265	SO_4364	-0.0367	0.9742	1.0000
SO_RS20270	SO_4365	-0.7153	0.3861	0.6147
SO_RS20275	SO_4366	0.0255	0.9861	1.0000
SO_RS20280	SO_4367	-3.8929	0.0140	0.1072
SO_RS20285	SO_4368	-4.6770	0.0792	0.2460
SO_RS20290	SO_4369	-6.1866	0.0429	0.1792
SO_RS20295	SO_4370	-8.0432	0.0129	0.1035
SO_RS20300	SO_4371	-0.9728	0.3682	0.5975
SO_RS20305	SO_4372	0.0000	1.0000	1.0000
SO_RS20310	SO_4373	-0.9873	0.6040	0.7983
SO_RS20315	SO_4374	-0.9837	0.3410	0.5693

SO_RS08495	SO_1849	7.2895	0.0207	0.1260
SO_RS08500	SO_1850	0.0000	1.0000	1.0000
SO_RS08525	SO_1851	1.3509	0.1144	0.3019
SO_RS08530	SO_1852	0.0000	1.0000	1.0000
SO_RS08535	SO_1853	-2.0958	0.0060	0.0725
SO_RS08540	SO_1854	-0.5564	0.3280	0.5569
SO_RS08545	SO_1855	0.2520	0.8784	1.0000
SO_RS08550	SO_1856	1.6334	0.1651	0.3705
SO_RS08555	SO_1857	-2.6109	0.0102	0.0938
SO_RS08575	SO_1860	3.0764	0.0299	0.1468
SO_RS08580	SO_1861	-5.2827	0.0022	0.0463
SO_RS08585	SO_1862	-2.1249	0.1736	0.3816
SO_RS08600	SO_t035	-3.2353	0.2294	0.4506
SO_RS08610	SO_1863	0.0000	1.0000	1.0000
SO_RS08615	SO_1864	0.0000	1.0000	1.0000
SO_RS08620	SO_1865	-3.7390	0.0571	0.2125
SO_RS08625	SO_1866	4.3587	0.0992	0.2790
SO_RS08630	SO_1867	0.6207	0.6953	0.8731
SO_RS08635	SO_1868	0.2831	0.8178	0.9644
SO_RS08640	SO_1870	0.3820	0.4266	0.6557
SO_RS08645	SO_1871	-0.0081	0.9941	1.0000
SO_RS08650	SO_1872	-0.4292	0.7691	0.9300
SO_RS08655	SO_1873	0.9196	0.6135	0.8064
SO_RS08670		2.0860	0.4537	0.6738
SO_RS08675	SO_1877	-0.6962	0.5513	0.7528
SO_RS08680	SO_1878	3.1514	0.1899	0.4026
SO_RS08685	SO_1879	-0.9743	0.3873	0.6162
SO_RS08690	SO_1880	-1.2763	0.0086	0.0868
SO_RS08695	SO_1881	5.3173	0.0751	0.2417
SO_RS08700	SO_1882	-0.2953	0.7096	0.8838
SO_RS08705	SO_1883	2.0861	0.4807	0.6972
SO_RS08710	SO_1884	-10.8186	0.0137	0.1061
SO_RS08715	SO_1886	0.0000	1.0000	1.0000
SO_RS08720	SO_1887	-3.0212	0.1311	0.3240
SO_RS08730	SO_1888	2.8661	0.2446	0.4657
SO_RS08735	SO_1889	0.0000	1.0000	1.0000
SO_RS08740	SO_1890	1.2415	0.2186	0.4385
SO_RS08745	SO_1891	1.8068	0.0263	0.1382
SO_RS08750	SO_1892	0.7073	0.3954	0.6240
SO_RS08755	SO_1893	1.1464	0.1865	0.3983
SO_RS08760	SO_1894	-0.1578	0.7277	0.8980
SO_RS08765	SO_1895	-1.1989	0.1779	0.3868
SO_RS08770	SO_1896	-1.8438	0.0187	0.1205
SO_RS08775	SO_1897	-2.6028	0.0237	0.1304
SO_RS08780	SO_1898	-6.1330	0.0318	0.1510
SO_RS08785	SO_1899	2.5400	0.1172	0.3061
SO_RS08790	SO_1900	2.1875	0.0491	0.1935
SO_RS08795	SO_1901	1.9821	0.3482	0.5760
SO_RS08800	SO_1902	2.3508	0.0577	0.2142
SO_RS08805		-1.6320	0.2961	0.5193
SO_RS08815	SO_1907	6.7649	0.0284	0.1436
SO_RS08820	SO_1909	-0.2753	0.8820	1.0000
SO_RS08825	SO_1910	4.1829	0.0145	0.1094
SO_RS08830	SO_1911	-3.9133	0.0041	0.0600
SO_RS08835	SO_1912	-1.2833	0.2609	0.4826
SO_RS08840	SO_1913	-4.6710	0.0645	0.2254
SO_RS08845	SO_1915	1.1485	0.1402	0.3378
SO_RS08850	SO_1916	8.0108	0.0057	0.0706
SO_RS08855	SO_1917	4.8115	0.0000	0.0022
SO_RS08860	SO_1918	2.7184	0.0420	0.1778
SO_RS08865	SO_1921	0.0000	1.0000	1.0000
SO_RS08870	SO_1922	5.8960	0.0300	0.1469
SO_RS08875	SO_1923	4.1202	0.0207	0.1260
SO_RS08880	SO_1924	1.6538	0.2818	0.5043
SO_RS08885	SO_1925	-0.9346	0.2485	0.4706
SO_RS08890	SO_1926	1.0934	0.0091	0.0883
SO_RS08895	SO_1927	1.7531	0.1652	0.3706
SO_RS08900	SO_4778	-0.8753	0.5345	0.7396

SO_RS20320	SO_4375	2.0860	0.4368	0.6633
SO_RS20325	SO_4376	-0.3269	0.8895	1.0000
SO_RS20330	SO_4377	5.3603	0.0056	0.0697
SO_RS20335	SO_4378	0.2551	0.8392	0.9785
SO_RS20340	SO_4379	0.0000	1.0000	1.0000
SO_RS20345	SO_4380	-2.3373	0.2650	0.4861
SO_RS20350	SO_4381	0.0000	1.0000	1.0000
SO_RS20355	SO_4382	-3.1786	0.1647	0.3700
SO_RS20360	SO_4383	2.4354	0.1854	0.3966
SO_RS20365	SO_4384	-1.2207	0.4519	0.6723
SO_RS20370	SO_4385	-1.7237	0.3401	0.5686
SO_RS20380		-3.3386	0.1452	0.3437
SO_RS20385	SO_4388	-1.0381	0.5486	0.7509
SO_RS20395	SO_4390	-5.2018	0.0315	0.1500
SO_RS20400	SO_4391	0.1488	0.8985	1.0000
SO_RS20405	SO_4393	-7.7569	0.0179	0.1189
SO_RS20410	SO_4394	1.8194	0.3188	0.5471
SO_RS20415	SO_4396	-0.1982	0.7056	0.8812
SO_RS20420	SO_4397	1.4038	0.4978	0.7119
SO_RS20425	SO_4398	-1.1328	0.2942	0.5170
SO_RS20430	SO_4399	9.1407	0.0014	0.0369
SO_RS20435	SO_4400	0.8454	0.7114	0.8850
SO_RS20440	SO_4401	2.1418	0.1644	0.3696
SO_RS20445	SO_4402	0.0000	1.0000	1.0000
SO_RS20450	SO_4403	-0.2805	0.8167	0.9637
SO_RS20455	SO_4404	-5.3690	0.0171	0.1175
SO_RS20460	SO_4405	1.6974	0.0541	0.2063
SO_RS20465	SO_4407	0.2912	0.8512	0.9881
SO_RS20470	SO_4408	0.7981	0.2568	0.4777
SO_RS20475	SO_4410	2.2555	0.0226	0.1291
SO_RS20480		0.0000	1.0000	1.0000
SO_RS20490	SO_4413	8.8883	0.0072	0.0787
SO_RS20495	SO_4414	7.4305	0.0275	0.1418
SO_RS20500	SO_4417	-0.6141	0.4312	0.6591
SO_RS20505	SO_4418	0.0000	1.0000	1.0000
SO_RS20510	SO_4419	-3.0350	0.1446	0.3437
SO_RS20515	SO_4420	4.8081	0.0847	0.2558
SO_RS20520	SO_4421	-4.6774	0.1079	0.2904
SO_RS20525	SO_4422	-1.3425	0.0269	0.1398
SO_RS20535	SO_4425	-2.5098	0.1793	0.3883
SO_RS20540	SO_4426	-0.5912	0.7643	0.9263
SO_RS20545	SO_4427	1.6039	0.2523	0.4738
SO_RS20550	SO_4428	-1.0034	0.4350	0.6615
SO_RS20555	SO_4429	0.6661	0.5259	0.7331
SO_RS20565	SO_4432	0.3455	0.8735	1.0000
SO_RS20570		0.0000	1.0000	1.0000
SO_RS20595		-9.9608	0.0236	0.1304
SO_RS20600	SO_4439	3.0836	0.1394	0.3363
SO_RS20605		0.0000	1.0000	1.0000
SO_RS20615		-4.1341	0.1111	0.2959
SO_RS20620	SO_4444	3.5777	0.1466	0.3453
SO_RS20625	SO_4445	-5.6553	0.0102	0.0938
SO_RS20630	SO_4446	-1.6333	0.1890	0.4016
SO_RS20635	SO_4447	0.4492	0.8297	0.9711
SO_RS20640	SO_4448	-2.8884	0.0103	0.0943
SO_RS20645	SO_4449	-3.2200	0.0751	0.2417
SO_RS20650	SO_4450	5.3277	0.0769	0.2438
SO_RS20655	SO_4451	-0.1106	0.9125	1.0000
SO_RS20660	SO_4452	1.3289	0.0761	0.2423
SO_RS20665	SO_4453	0.5811	0.2818	0.5043
SO_RS20670	SO_4454	0.8319	0.3896	0.6185
SO_RS20675	SO_4455	6.7145	0.0190	0.1206
SO_RS20680	SO_4456	3.7677	0.0036	0.0579
SO_RS20685	SO_4457	-2.0578	0.2619	0.4834
SO_RS20690	SO_4458	-0.9732	0.3220	0.5502
SO_RS20695	SO_4461	2.0907	0.4986	0.7124
SO_RS20700	SO_4462	-3.2638	0.0078	0.0830
SO_RS20705	SO_4463	1.0020	0.6351	0.8273

SO_RS08905	SO_1928	1.4080	0.0073	0.0791
SO_RS08910	SO_1929	2.8779	0.0093	0.0899
SO_RS08915	SO_1930	0.6969	0.0602	0.2185
SO_RS08920	SO_1931	1.4986	0.0054	0.0679
SO_RS08925	SO_1932	0.8973	0.0322	0.1520
SO_RS08930	SO_1933	2.4344	0.0043	0.0615
SO_RS08935	SO_1935	1.4379	0.3916	0.6204
SO_RS08940	SO_1936	1.6282	0.3515	0.5801
SO_RS08945	SO_1937	-1.7484	0.2494	0.4709
SO_RS08950	SO_1938	0.4756	0.6127	0.8057
SO_RS08955	SO_1939	-1.9329	0.3547	0.5835
SO_RS08960	SO_1940	2.0859	0.4290	0.6575
SO_RS08965	SO_1941	1.9665	0.1214	0.3120
SO_RS08970	SO_1942	1.9446	0.1059	0.2889
SO_RS08975	SO_1944	-1.7330	0.3954	0.6240
SO_RS08980	SO_1945	-1.9779	0.1287	0.3203
SO_RS08985	SO_1946	-0.8149	0.4177	0.6464
SO_RS08990	SO_1947	7.6920	0.0134	0.1050
SO_RS08995	SO_1948	2.1887	0.0806	0.2487
SO_RS09000	SO_1949	-0.6372	0.2924	0.5156
SO_RS09005		0.0000	1.0000	1.0000
SO_RS09015		2.0861	0.5507	0.7528
SO_RS09020	SO_1952	1.6357	0.1450	0.3437
SO_RS09025	SO_1953	7.5354	0.0289	0.1448
SO_RS09030	SO_1954	4.5683	0.0891	0.2632
SO_RS09035	SO_1955	0.0000	1.0000	1.0000
SO_RS09040	SO_1956	-3.2346	0.1092	0.2927
SO_RS09045	SO_1957	4.0920	0.0311	0.1492
SO_RS09050	SO_1958	8.6174	0.0104	0.0944
SO_RS09055	SO_1959	8.3277	0.0161	0.1148
SO_RS09060	SO_1960	3.8137	0.2142	0.4331
SO_RS09065	SO_1961	2.9630	0.1041	0.2860
SO_RS09070	SO_1962	1.2895	0.1524	0.3524
SO_RS09075	SO_1963	-0.0471	0.9730	1.0000
SO_RS09080	SO_1965	-5.8015	0.0175	0.1182
SO_RS09085	SO_1966	2.5032	0.0064	0.0762
SO_RS09090	SO_1967	3.0203	0.2110	0.4290
SO_RS09095	SO_1968	-2.9998	0.1232	0.3141
SO_RS09100	SO_1969	-2.8690	0.0816	0.2501
SO_RS09105	SO_1970	-1.1796	0.2930	0.5162
SO_RS09110	SO_1971	1.1895	0.3146	0.5418
SO_RS09115	SO_4823	2.9041	0.3144	0.5416
SO_RS09120		0.1603	0.8541	0.9898
SO_RS09130	SO_1975	-0.9833	0.6490	0.8393
SO_RS09135	SO_1976	-1.5893	0.4787	0.6961
SO_RS09140	SO_1977	-1.2511	0.4307	0.6589
SO_RS09145	SO_1978	-6.7532	0.0250	0.1338
SO_RS09150	SO_1979	4.2496	0.0146	0.1095
SO_RS09155	SO_1980	-0.1178	0.9286	1.0000
SO_RS09160	SO_1981	2.8557	0.0844	0.2554
SO_RS09165	SO_1983	-4.1336	0.1153	0.3030
SO_RS09170	SO_1985	1.5777	0.0050	0.0667
SO_RS09175	SO_1986	0.2933	0.4086	0.6371
SO_RS09180	SO_1987	-0.9978	0.2295	0.4506
SO_RS09185	SO_1988	-0.0633	0.9231	1.0000
SO_RS09190	SO_1989	-0.4594	0.3370	0.5647
SO_RS09195	SO_1990	-1.0867	0.5462	0.7486
SO_RS09200	SO_1991	-0.0744	0.9395	1.0000
SO_RS09205	SO_1994	1.1347	0.2071	0.4237
SO_RS09210	SO_1995	0.2353	0.8630	0.9975
SO_RS09215	SO_1997	-0.4632	0.8134	0.9622
SO_RS09220	SO_1998	-10.4321	0.0151	0.1109
SO_RS09225	SO_1999	-0.2082	0.8934	1.0000
SO_RS09230	SO_2001	3.0189	0.0003	0.0149
SO_RS09235	SO_2002	0.2234	0.8890	1.0000
SO_RS09240	SO_2004	-4.1338	0.1630	0.3679
SO_RS09245	SO_2005	1.1912	0.3059	0.5318
SO_RS09250	SO_2006	-1.3809	0.4500	0.6703

SO_RS20710	SO_4465	-0.3744	0.5017	0.7144
SO_RS20715	SO_4466	1.2164	0.4835	0.6989
SO_RS20720	SO_4467	-5.5328	0.0399	0.1740
SO_RS20725	SO_4468	3.3839	0.0799	0.2470
SO_RS20730	SO_4469	-0.7782	0.5797	0.7762
SO_RS20735	SO_4470	1.1367	0.4733	0.6904
SO_RS20740	SO_4471	2.0975	0.2373	0.4579
SO_RS20745	SO_4472	-0.6377	0.6950	0.8731
SO_RS20750	SO_4473	0.8886	0.5868	0.7832
SO_RS20755	SO_4474	-3.5548	0.1297	0.3219
SO_RS20760	SO_4475	1.7134	0.2332	0.4535
SO_RS20765	SO_4476	1.3422	0.1019	0.2829
SO_RS20770	SO_4477	2.2792	0.2341	0.4544
SO_RS20775	SO_4478	9.4260	0.0022	0.0463
SO_RS20780	SO_4479	-0.9529	0.3782	0.6071
SO_RS20785	SO_4480	4.0955	0.0228	0.1291
SO_RS20790	SO_4481	6.5171	0.0719	0.2373
SO_RS20795	SO_4482	6.4138	0.0377	0.1682
SO_RS20800	SO_4483	-3.9514	0.0783	0.2453
SO_RS20805	SO_4484	-2.8724	0.2130	0.4315
SO_RS20810	SO_4485	0.8635	0.6863	0.8661
SO_RS20815	SO_4486	2.0908	0.4452	0.6682
SO_RS20820	SO_4487	-0.7807	0.6113	0.8045
SO_RS20825	SO_4488	-4.6477	0.0160	0.1145
SO_RS20830	SO_4489	-4.1296	0.0778	0.2442
SO_RS20835	SO_4490	0.0000	1.0000	1.0000
SO_RS20840	SO_4492	0.9020	0.0820	0.2509
SO_RS20880	SO_4497	-1.3172	0.3163	0.5438
SO_RS20885	SO_4498	0.0000	1.0000	1.0000
SO_RS20910	SO_r023	-8.9358	0.0234	0.1303
SO_RS20920	SO_4502	0.7459	0.3360	0.5645
SO_RS20925	SO_4503	0.4812	0.6937	0.8719
SO_RS20930	SO_4504	4.5176	0.0141	0.1080
SO_RS20935	SO_4505	8.5674	0.0022	0.0463
SO_RS20940	SO_4506	3.4541	0.0008	0.0277
SO_RS20945	SO_4507	0.8941	0.1396	0.3367
SO_RS20950	SO_4508	2.0908	0.4902	0.7043
SO_RS20955	SO_4509	-1.0121	0.0193	0.1210
SO_RS20960	SO_4510	0.9326	0.2206	0.4408
SO_RS20965	SO_4511	-0.9772	0.1105	0.2953
SO_RS20970	SO_4512	0.0000	1.0000	1.0000
SO_RS20975	SO_4513	-2.1767	0.0017	0.0422
SO_RS20980	SO_4514	-2.3068	0.0087	0.0869
SO_RS20985	SO_4515	-3.0279	0.0001	0.0079
SO_RS20990	SO_4516	0.6639	0.4480	0.6697
SO_RS20995	SO_4519	8.5861	0.0040	0.0600
SO_RS21000	SO_4520	-1.2320	0.0320	0.1516
SO_RS21005	SO_4521	0.0000	1.0000	1.0000
SO_RS21010	SO_4522	0.0000	1.0000	1.0000
SO_RS21015	SO_4523	0.3587	0.7487	0.9132
SO_RS21020	SO_4524	6.2520	0.0848	0.2558
SO_RS21025	SO_4525	0.2633	0.8753	1.0000
SO_RS21030	SO_4526	-0.4882	0.7437	0.9084
SO_RS21035	SO_4527	-6.6088	0.0454	0.1856
SO_RS21040	SO_4528	7.0271	0.0394	0.1731
SO_RS21045	SO_4529	7.3757	0.0235	0.1303
SO_RS21050	SO_4530	8.2287	0.0053	0.0673
SO_RS21060	SO_4533	-2.4406	0.2471	0.4694
SO_RS21065		0.0000	1.0000	1.0000
SO_RS21070	SO_4535	-0.8042	0.6497	0.8393
SO_RS21075	SO_4537	8.9620	0.0000	0.0053
SO_RS21080	SO_4538	1.0031	0.2798	0.5023
SO_RS21085	SO_4811	-1.6547	0.1859	0.3973
SO_RS21095	SO_4542	-8.2239	0.0153	0.1113
SO_RS21100	SO_4543	7.5249	0.0513	0.1977
SO_RS21105		0.0000	1.0000	1.0000
SO_RS21115		-5.2827	0.0734	0.2405
SO_RS21125	SO_4547	-1.0816	0.5410	0.7453

SO_RS09255	SO_2007	2.1787	0.0076	0.0816
SO_RS09260	SO_2008	2.0246	0.0041	0.0601
SO_RS09265	SO_2009	1.7606	0.2950	0.5176
SO_RS09270	SO_2011	1.5118	0.3458	0.5735
SO_RS09275	SO_2012	0.9392	0.6173	0.8094
SO_RS09280	SO_2013	-0.9148	0.2695	0.4919
SO_RS09285	SO_2014	-3.0580	0.0193	0.1210
SO_RS09290	SO_2015	1.2384	0.3684	0.5977
SO_RS09295	SO_2016	1.8934	0.0063	0.0752
SO_RS09300	SO_2017	0.2655	0.7415	0.9067
SO_RS09305	SO_2018	1.5589	0.0250	0.1338
SO_RS09310	SO_2019	-2.9175	0.1262	0.3176
SO_RS09315	SO_2020	0.2380	0.8228	0.9669
SO_RS09320	SO_2021	-2.4284	0.1191	0.3087
SO_RS09325	SO_2022	3.8034	0.2209	0.4408
SO_RS09330	SO_2023	-11.5126	0.0077	0.0819
SO_RS09345	SO_2027	-2.4438	0.1920	0.4046
SO_RS09355	SO_4779	0.0000	1.0000	1.0000
SO_RS09365	SO_2033	6.1887	0.0829	0.2529
SO_RS09370	SO_2034	3.4258	0.1958	0.4075
SO_RS09380	SO_2036	0.0000	1.0000	1.0000
SO_RS09385	SO_2037	-0.4463	0.8095	0.9594
SO_RS09390	SO_2039	0.0000	1.0000	1.0000
SO_RS09395	SO_2040	2.4321	0.0500	0.1948
SO_RS09400	SO_2041	8.2062	0.0405	0.1754
SO_RS09405	SO_2042	2.2719	0.0005	0.0214
SO_RS09410	SO_2043	2.4482	0.0014	0.0372
SO_RS09415	SO_2044	1.3992	0.3821	0.6106
SO_RS09420	SO_2045	1.2602	0.3016	0.5264
SO_RS09425	SO_2046	-0.7694	0.4380	0.6639
SO_RS09430	SO_2047	2.8000	0.0549	0.2077
SO_RS09435	SO_2048	-6.1974	0.0012	0.0339
SO_RS09440	SO_2049	-1.8441	0.1936	0.4062
SO_RS09445	SO_2050	-3.0180	0.1378	0.3339
SO_RS09450	SO_2052	5.9745	0.0075	0.0808
SO_RS09455	SO_2053	-10.8499	0.0108	0.0959
SO_RS09460	SO_2054	1.6862	0.3298	0.5584
SO_RS09465	SO_2055	8.8453	0.0045	0.0628
SO_RS09475	SO_2061	-2.5256	0.1811	0.3903
SO_RS09480	SO_2062	1.7952	0.2946	0.5171
SO_RS09485	SO_2063	-7.8868	0.0243	0.1318
SO_RS09490	SO_2064	2.4609	0.1142	0.3017
SO_RS09495	SO_2065	0.5351	0.7478	0.9124
SO_RS09500	SO_2067	0.8107	0.5065	0.7185
SO_RS09505	SO_2068	-2.4667	0.1362	0.3314
SO_RS09510	SO_2069	5.7228	0.0620	0.2215
SO_RS09515	SO_2070	2.9314	0.1078	0.2903
SO_RS09520	SO_2071	0.8537	0.4018	0.6308
SO_RS09525	SO_2072	-1.0647	0.5266	0.7334
SO_RS09530	SO_2073	1.7872	0.1182	0.3073
SO_RS09535	SO_2074	-1.1713	0.5004	0.7138
SO_RS09540	SO_2075	-5.6061	0.0668	0.2293
SO_RS09545	SO_2076	0.0000	1.0000	1.0000
SO_RS09550		0.0000	1.0000	1.0000
SO_RS09555	SO_2078	9.4951	0.0013	0.0359
SO_RS09560	SO_2079	-4.2909	0.1583	0.3610
SO_RS09565	SO_2081	-3.1783	0.0406	0.1756
SO_RS09570	SO_2082	-0.4774	0.8090	0.9590
SO_RS09575	SO_2083	2.8154	0.0032	0.0554
SO_RS09580		7.2428	0.0937	0.2706
SO_RS09585	SO_2085	0.0257	0.9648	1.0000
SO_RS09590	SO_2086	-0.2016	0.5744	0.7712
SO_RS09595	SO_2087	-0.6351	0.3258	0.5549
SO_RS09600	SO_2088	-1.4127	0.3779	0.6070
SO_RS09605	SO_2089	8.4517	0.0078	0.0830
SO_RS09610	SO_2090	6.2584	0.0007	0.0265
SO_RS09615	SO_2091	1.6366	0.1980	0.4103
SO_RS09620	SO_2092	3.4319	0.2919	0.5153

SO_RS21140	SO_4550	2.0688	0.2485	0.4706
SO_RS21145	SO_4551	-3.7462	0.1448	0.3437
SO_RS21150	SO_4552	-0.0001	0.9999	1.0000
SO_RS21155	SO_4554	-1.7579	0.3529	0.5817
SO_RS21160	SO_4555	-0.0249	0.9730	1.0000
SO_RS21165	SO_4556	8.5180	0.0209	0.1265
SO_RS21170	SO_4557	-1.0736	0.2938	0.5169
SO_RS21175	SO_4558	0.9116	0.4132	0.6412
SO_RS21180	SO_4559	-0.5245	0.6605	0.8462
SO_RS21185	SO_4560	2.7223	0.1511	0.3513
SO_RS21190	SO_4561	-2.7745	0.2669	0.4887
SO_RS21195	SO_4562	0.2066	0.9100	1.0000
SO_RS21200	SO_4563	-1.8980	0.0706	0.2352
SO_RS21205	SO_4564	2.0200	0.0742	0.2405
SO_RS21210	SO_4565	1.7817	0.3087	0.5353
SO_RS21215	SO_4567	-12.0980	0.0005	0.0214
SO_RS21220	SO_4568	-2.0678	0.2179	0.4380
SO_RS21230	SO_4570	0.0000	1.0000	1.0000
SO_RS21235	SO_4571	-1.8408	0.3203	0.5486
SO_RS21240	SO_4572	-5.3727	0.0607	0.2196
SO_RS21245	SO_4573	2.8286	0.0163	0.1155
SO_RS21250	SO_4574	8.0869	0.0082	0.0853
SO_RS21255	SO_4575	0.4703	0.7387	0.9057
SO_RS21260	SO_4576	7.8292	0.0707	0.2352
SO_RS21265	SO_4577	-4.0093	0.0825	0.2522
SO_RS21275	SO_4579	0.0000	1.0000	1.0000
SO_RS21290	SO_4583	-0.6303	0.0705	0.2352
SO_RS21295	SO_4584	0.8197	0.7121	0.8854
SO_RS21300	SO_4585	0.2258	0.8614	0.9964
SO_RS21305	SO_4586	-1.7940	0.0868	0.2593
SO_RS21310	SO_4587	5.1246	0.0710	0.2357
SO_RS21315	SO_4588	3.8136	0.2405	0.4612
SO_RS21320	SO_4589	2.2450	0.0279	0.1429
SO_RS21325	SO_4590	5.7337	0.0437	0.1811
SO_RS21330	SO_4591	-0.2867	0.5218	0.7309
SO_RS21335	SO_4592	-2.8534	0.0093	0.0899
SO_RS21340	SO_4593	4.6205	0.0425	0.1785
SO_RS21345	SO_4594	0.1717	0.8299	0.9711
SO_RS21350	SO_4596	2.4364	0.1478	0.3467
SO_RS21355	SO_4597	1.3163	0.4479	0.6697
SO_RS21360	SO_4598	4.3314	0.0004	0.0187
SO_RS21365	SO_4599	1.3856	0.3853	0.6139
SO_RS21370	SO_4600	0.7814	0.4662	0.6843
SO_RS21375	SO_4601	-1.1991	0.3233	0.5515
SO_RS21380	SO_4602	0.0087	0.9902	1.0000
SO_RS21385	SO_4603	1.4745	0.1168	0.3056
SO_RS21390	SO_4604	-1.1685	0.3322	0.5603
SO_RS21395	SO_4606	-0.3680	0.6402	0.8317
SO_RS21400	SO_4607	3.7402	0.0028	0.0502
SO_RS21405	SO_4608	7.9819	0.0249	0.1336
SO_RS21410	SO_4609	1.3573	0.3341	0.5625
SO_RS21415	SO_4610	2.9043	0.3736	0.6031
SO_RS21420	SO_4611	0.8178	0.5260	0.7331
SO_RS21425	SO_4612	5.8215	0.0649	0.2260
SO_RS21430	SO_4613	3.8059	0.1685	0.3744
SO_RS21435	SO_4614	4.2412	0.0585	0.2159
SO_RS21440	SO_4615	5.4342	0.0444	0.1832
SO_RS21445	SO_4616	10.2784	0.0005	0.0214
SO_RS21450	SO_4617	6.2408	0.0286	0.1441
SO_RS21455	SO_4618	2.8060	0.1523	0.3524
SO_RS21460	SO_4619	2.4438	0.1602	0.3637
SO_RS21465	SO_4620	-2.4879	0.0879	0.2614
SO_RS21470	SO_4621	3.4229	0.3636	0.5920
SO_RS21475	SO_4622	-8.6997	0.0145	0.1094
SO_RS21480	SO_4623	-5.6203	0.0468	0.1889
SO_RS21485	SO_4624	-0.7045	0.6704	0.8548
SO_RS21490	SO_4625	-7.1610	0.0190	0.1205
SO_RS21495	SO_4626	-6.4042	0.0283	0.1436

SO_RS09625	SO_2093	2.7001	0.0093	0.0899
SO_RS09630	SO_2094	-0.8245	0.4988	0.7124
SO_RS09635	SO_2095	0.5540	0.4316	0.6594
SO_RS09640	SO_2096	9.6760	0.0033	0.0556
SO_RS09645	SO_2097	2.3524	0.0068	0.0772
SO_RS09650	SO_2098	2.8771	0.0008	0.0281
SO_RS09655	SO_2099	2.4729	0.0006	0.0244
SO_RS09660	SO_2100	8.0373	0.0466	0.1886
SO_RS09665	SO_2101	-2.9816	0.1937	0.4062
SO_RS09670	SO_2102	-0.6570	0.7140	0.8872
SO_RS09675	SO_2103	2.5499	0.1311	0.3240
SO_RS09680	SO_2104	-1.9690	0.3019	0.5265
SO_RS09685	SO_2105	4.8166	0.0049	0.0661
SO_RS09690	SO_2107	1.2414	0.0287	0.1442
SO_RS09695	SO_2108	-0.9917	0.0235	0.1303
SO_RS09700	SO_2109	0.6333	0.6804	0.8619
SO_RS09705	SO_2110	0.4411	0.6505	0.8393
SO_RS09710	SO_2111	-2.5872	0.1773	0.3864
SO_RS09715	SO_2112	-0.1943	0.8727	1.0000
SO_RS09720	SO_2113	0.3656	0.7305	0.8999
SO_RS09725	SO_2114	2.6706	0.0638	0.2235
SO_RS09730	SO_2115	-1.5657	0.3016	0.5264
SO_RS09735	SO_2116	-0.1739	0.6572	0.8442
SO_RS09750	SO_2119	2.0907	0.5626	0.7621
SO_RS09755	SO_2120	0.0000	1.0000	1.0000
SO_RS09760	SO_2121	-2.6658	0.1980	0.4103
SO_RS09765	SO_2122	0.0000	1.0000	1.0000
SO_RS09770	SO_2123	2.9875	0.0742	0.2405
SO_RS09775	SO_2124	0.0000	1.0000	1.0000
SO_RS09780	SO_2125	-2.2726	0.0955	0.2733
SO_RS09785	SO_2126	-0.3290	0.8141	0.9622
SO_RS09790	SO_2127	5.3278	0.0607	0.2196
SO_RS09800	SO_2129	0.2096	0.9278	1.0000
SO_RS09810		0.0000	1.0000	1.0000
SO_RS09815	SO_2133	6.0382	0.1432	0.3422
SO_RS09825	SO_2136	-0.1955	0.7023	0.8789
SO_RS09830	SO_2137	-1.4948	0.4488	0.6703
SO_RS09845	SO_2141	1.3677	0.5694	0.7679
SO_RS09850		1.9426	0.3768	0.6064
SO_RS09855	SO_2143	-0.8149	0.6383	0.8301
SO_RS09860	SO_2144	-1.2760	0.3523	0.5810
SO_RS09865	SO_2145	-0.7103	0.7405	0.9061
SO_RS09870	SO_2146	6.3113	0.0776	0.2438
SO_RS09875	SO_2147	-1.2880	0.1537	0.3532
SO_RS09880	SO_2148	0.7765	0.3745	0.6041
SO_RS09885	SO_2149	0.2101	0.7941	0.9474
SO_RS09890	SO_2150	4.5648	0.1563	0.3578
SO_RS09895	SO_2151	1.1428	0.5047	0.7164
SO_RS09900	SO_2153	4.6935	0.0113	0.0981
SO_RS09905	SO_2154	2.1411	0.2062	0.4230
SO_RS09910	SO_2155	3.8136	0.2386	0.4590
SO_RS09915	SO_2156	5.8094	0.0478	0.1913
SO_RS09925		0.0000	1.0000	1.0000
SO_RS09935		-0.9187	0.6791	0.8613
SO_RS09945		-0.0087	0.9959	1.0000
SO_RS09950	SO_2164	-0.2307	0.8757	1.0000
SO_RS09965		-0.4424	0.8090	0.9590
SO_RS09975		-2.1633	0.3218	0.5502
SO_RS09980	SO_4781	-5.7024	0.0315	0.1500
SO_RS09990	SO_2174	-3.1599	0.1169	0.3056
SO_RS09995	SO_2175	1.0448	0.5029	0.7152
SO_RS10000	SO_2176	-1.3900	0.4804	0.6970
SO_RS10005	SO_2177	-2.4936	0.1422	0.3403
SO_RS10010	SO_2178	3.6549	0.0211	0.1268
SO_RS10015	SO_2179	1.4534	0.0749	0.2414
SO_RS10020	SO_2180	-1.3915	0.4815	0.6978
SO_RS10025	SO_2181	-1.2503	0.5722	0.7693
SO_RS10030	SO_2182	0.0000	1.0000	1.0000

SO_RS21500	SO_4627	0.8742	0.1675	0.3735
SO_RS21505	SO_4628	-1.3253	0.2781	0.5007
SO_RS21510	SO_4629	-0.2267	0.6193	0.8110
SO_RS21515	SO_4631	1.0559	0.4524	0.6726
SO_RS21520	SO_4633	1.9369	0.0366	0.1647
SO_RS21525	SO_4634	0.6022	0.6627	0.8480
SO_RS21530	SO_4635	9.3515	0.0023	0.0465
SO_RS21535	SO_4636	-2.3480	0.2219	0.4418
SO_RS21540	SO_4637	-0.4189	0.7765	0.9371
SO_RS21545	SO_4638	6.1196	0.0452	0.1850
SO_RS21550	SO_4639	0.0000	1.0000	1.0000
SO_RS21555	SO_4640	0.4696	0.8064	0.9569
SO_RS21560	SO_4641	-8.7468	0.0066	0.0767
SO_RS21565	SO_4642	2.1985	0.2754	0.4977
SO_RS21570	SO_4643	3.8047	0.0628	0.2224
SO_RS21575	SO_4812	0.2333	0.6084	0.8020
SO_RS21580	SO_4645	-2.6399	0.0548	0.2076
SO_RS21585	SO_4646	5.3290	0.0735	0.2405
SO_RS21590	SO_4647	-0.6311	0.7063	0.8813
SO_RS21595	SO_4648	-1.4793	0.4323	0.6598
SO_RS21600	SO_4649	-0.1219	0.9122	1.0000
SO_RS21605	SO_4650	2.5178	0.0047	0.0655
SO_RS21610	SO_4651	1.3046	0.4820	0.6978
SO_RS21615	SO_4652	4.9154	0.0003	0.0149
SO_RS21620	SO_4653	2.0466	0.0354	0.1605
SO_RS21625	SO_4654	10.9160	0.0003	0.0176
SO_RS21630	SO_4655	3.6363	0.0421	0.1779
SO_RS21635	SO_4656	4.5755	0.1030	0.2844
SO_RS21640	SO_4657	-1.3935	0.0668	0.2293
SO_RS21650		-2.7696	0.2513	0.4726
SO_RS21655	SO_4658	-3.2143	0.0027	0.0502
SO_RS21660	SO_4659	1.7138	0.0835	0.2536
SO_RS21665	SO_4660	-2.1515	0.2478	0.4703
SO_RS21670	SO_4661	-2.4227	0.2858	0.5091
SO_RS21675	SO_4662	-1.6325	0.2609	0.4826
SO_RS21680	SO_4664	6.9905	0.0937	0.2706
SO_RS21690	SO_4666	-0.1430	0.7525	0.9170
SO_RS21695	SO_4667	-1.4310	0.1796	0.3888
SO_RS21700	SO_4669	-0.3552	0.5092	0.7212
SO_RS21705	SO_4670	4.1801	0.0059	0.0718
SO_RS21710	SO_4671	-0.8507	0.4761	0.6932
SO_RS21715	SO_4672	1.6285	0.3109	0.5375
SO_RS21720	SO_4673	3.9125	0.0000	0.0042
SO_RS21725	SO_4674	4.7776	0.0000	0.0035
SO_RS21730	SO_4675	5.3161	0.1182	0.3073
SO_RS21735	SO_4676	-1.0409	0.4929	0.7066
SO_RS21740	SO_4677	-2.0406	0.1385	0.3348
SO_RS21745	SO_4678	-0.7910	0.6490	0.8393
SO_RS21750	SO_4679	-0.4013	0.6602	0.8461
SO_RS21755	SO_4680	0.0711	0.9364	1.0000
SO_RS21760	SO_4681	0.9838	0.2305	0.4518
SO_RS21765	SO_4682	1.3543	0.4139	0.6418
SO_RS21770	SO_4684	-1.2464	0.4562	0.6768
SO_RS21775	SO_4685	-2.8576	0.0148	0.1100
SO_RS21780	SO_4686	0.9784	0.5104	0.7218
SO_RS21785	SO_4687	-0.9885	0.3361	0.5645
SO_RS21790	SO_4688	2.2451	0.0327	0.1531
SO_RS21795	SO_4689	3.0815	0.1036	0.2852
SO_RS21800	SO_4690	-4.2759	0.0214	0.1271
SO_RS21810	SO_4692	0.5599	0.1528	0.3524
SO_RS21815	SO_4693	0.2065	0.6222	0.8143
SO_RS21820	SO_4694	8.8859	0.0053	0.0673
SO_RS21825	SO_4696	1.6991	0.1189	0.3084
SO_RS21830	SO_4697	1.5264	0.3850	0.6138
SO_RS21835	SO_4698	1.4989	0.5157	0.7271
SO_RS21840	SO_4699	0.7658	0.0531	0.2029
SO_RS21845	SO_4700	-4.6718	0.0615	0.2204
SO_RS21850	SO_4701	-4.4916	0.0591	0.2170

SO_RS10035	SO_2183	5.4904	0.0057	0.0707
SO_RS10040	SO_2185	8.7016	0.0136	0.1059
SO_RS10045		-6.6272	0.0069	0.0772
SO_RS10055		7.0495	0.0007	0.0265
SO_RS10060	SO_2189	-1.6741	0.0940	0.2709
SO_RS10065	SO_2190	-0.3484	0.5925	0.7890
SO_RS10070	SO_2191	4.4298	0.0117	0.0988
SO_RS10075	SO_2192	7.0636	0.0277	0.1426
SO_RS10080	SO_2193	-0.5893	0.7292	0.8991
SO_RS10085	SO_2194	0.0000	1.0000	1.0000
SO_RS10090	SO_2195	4.3587	0.1062	0.2889
SO_RS10095	SO_2196	-2.8711	0.2211	0.4408
SO_RS10100	SO_2197	-0.2952	0.6615	0.8467
SO_RS10105	SO_2198	-1.8515	0.0788	0.2453
SO_RS10110	SO_2199	-10.6692	0.0102	0.0938
SO_RS10115	SO_2200	-1.2981	0.4623	0.6818
SO_RS10120	SO_2201	1.2505	0.1286	0.3203
SO_RS10125	SO_2202	7.8825	0.0129	0.1035
SO_RS10130	SO_2203	0.0000	1.0000	1.0000
SO_RS10135	SO_2204	5.4396	0.0473	0.1909
SO_RS10145	SO_2208	0.1871	0.9213	1.0000
SO_RS10150	SO_2210	-1.8509	0.1138	0.3010
SO_RS10170	SO_2213	-0.9080	0.3688	0.5980
SO_RS10175	SO_2214	-0.2909	0.8262	0.9683
SO_RS10180	SO_2215	-0.5978	0.5930	0.7894
SO_RS10185	SO_2216	-2.5611	0.1577	0.3601
SO_RS10190	SO_2217	1.2104	0.2971	0.5204
SO_RS10195	SO_2218	2.2226	0.0601	0.2185
SO_RS10200	SO_2219	-1.9107	0.1433	0.3423
SO_RS10205	SO_2220	-0.8187	0.5921	0.7889
SO_RS10210	SO_2221	3.2815	0.1445	0.3437
SO_RS10215	SO_2222	2.5604	0.0004	0.0200
SO_RS10220	SO_2223	-0.2231	0.7949	0.9481
SO_RS10225	SO_2224	0.0000	1.0000	1.0000
SO_RS10230	SO_2225	4.7545	0.0785	0.2453
SO_RS10235		0.0000	1.0000	1.0000
SO_RS10245	SO_2228	1.0529	0.3001	0.5246
SO_RS10250	SO_2229	-1.8467	0.0161	0.1148
SO_RS10260		0.0000	1.0000	1.0000
SO_RS10265	SO_2234	0.0000	1.0000	1.0000
SO_RS10270	SO_2236	1.5078	0.1214	0.3120
SO_RS10275	SO_2237	-0.8597	0.3777	0.6070
SO_RS10280	SO_2238	-3.9322	0.0774	0.2438
SO_RS10285	SO_2239	7.7791	0.0100	0.0932
SO_RS10290	SO_2240	0.3354	0.6582	0.8449
SO_RS10295	SO_2241	-1.6776	0.1115	0.2967
SO_RS10300	SO_2244	-1.3361	0.4333	0.6602
SO_RS10305	SO_2245	0.0556	0.9771	1.0000
SO_RS10310	SO_2247	4.0135	0.0147	0.1098
SO_RS10315	SO_2248	2.6238	0.0048	0.0659
SO_RS10320		0.0000	1.0000	1.0000
SO_RS10325	SO_2250	-0.9490	0.4786	0.6961
SO_RS10330	SO_2251	0.5903	0.3408	0.5692
SO_RS10335	SO_2253	-3.3371	0.2568	0.4777
SO_RS10340	SO_2254	1.0861	0.2663	0.4881
SO_RS10345	SO_2255	-0.6300	0.4324	0.6598
SO_RS10350	SO_2256	-0.7008	0.7531	0.9171
SO_RS10355	SO_2257	5.2003	0.0512	0.1975
SO_RS10360	SO_2258	-3.7875	0.0511	0.1974
SO_RS10365	SO_2259	0.5826	0.6698	0.8545
SO_RS10370	SO_2260	-1.9028	0.0951	0.2732
SO_RS10375	SO_2261	1.3377	0.5513	0.7528
SO_RS10380	SO_2262	-1.9080	0.0569	0.2122
SO_RS10385	SO_2263	6.1770	0.0284	0.1436
SO_RS10390	SO_2264	-0.4220	0.6085	0.8020
SO_RS10395	SO_2265	-1.8102	0.1282	0.3198
SO_RS10400	SO_2266	-2.7186	0.2217	0.4415
SO_RS10405	SO_2267	1.0135	0.5936	0.7897

SO_RS21855	SO_4702	1.9889	0.1263	0.3176
SO_RS21860	SO_4704	2.3786	0.2180	0.4381
SO_RS21865	SO_4705	0.0044	0.9979	1.0000
SO_RS21870	SO_4706	10.2837	0.0008	0.0269
SO_RS21880	SO_4708	1.5850	0.4332	0.6602
SO_RS21885	SO_4709	0.0000	1.0000	1.0000
SO_RS21890	SO_4710	0.3390	0.8213	0.9660
SO_RS21895	SO_4711	-0.2482	0.6571	0.8442
SO_RS21900	SO_4712	-1.8398	0.3908	0.6199
SO_RS21905	SO_4713	0.3289	0.7370	0.9041
SO_RS21910	SO_4714	0.0567	0.9386	1.0000
SO_RS21915	SO_4715	-1.3010	0.3296	0.5583
SO_RS21920	SO_4716	0.7944	0.6356	0.8276
SO_RS21925	SO_4717	-3.5425	0.0068	0.0772
SO_RS21930	SO_4718	0.1154	0.8713	1.0000
SO_RS21935	SO_4719	2.8040	0.0011	0.0330
SO_RS21940	SO_4720	2.9118	0.3131	0.5402
SO_RS21945	SO_4721	9.0225	0.0063	0.0752
SO_RS21950	SO_4722	-2.5157	0.2066	0.4233
SO_RS21955	SO_4723	-1.3936	0.0668	0.2293
SO_RS21960	SO_4724	7.1004	0.0149	0.1102
SO_RS21965	SO_4725	1.7511	0.1528	0.3524
SO_RS21970	SO_4726	-2.1172	0.0983	0.2772
SO_RS21975	SO_4727	0.0000	1.0000	1.0000
SO_RS21980	SO_4728	-7.3423	0.0025	0.0490
SO_RS21985	SO_4729	0.0000	1.0000	1.0000
SO_RS21990	SO_4730	1.8277	0.1212	0.3119
SO_RS21995	SO_4731	4.4811	0.0011	0.0333
SO_RS22000	SO_4732	-1.5750	0.1533	0.3532
SO_RS22005	SO_4733	9.2452	0.0033	0.0554
SO_RS22010	SO_4734	-1.8621	0.0079	0.0830
SO_RS22030	SO_4737	1.2874	0.0773	0.2438
SO_RS22035	SO_4738	1.3027	0.3428	0.5706
SO_RS22040	SO_4739	5.6539	0.0023	0.0465
SO_RS22045	SO_4740	0.0000	1.0000	1.0000
SO_RS22050	SO_4741	-0.0950	0.8867	1.0000
SO_RS22055	SO_4742	-0.5196	0.8137	0.9622
SO_RS22060	SO_4743	-0.2024	0.7722	0.9324
SO_RS22065	SO_4745	-1.0952	0.2869	0.5102
SO_RS22070	SO_4746	2.0239	0.0347	0.1585
SO_RS22075	SO_4747	1.9806	0.0001	0.0095
SO_RS22080	SO_4748	1.1516	0.0121	0.0996
SO_RS22085	SO_4749	0.7266	0.0500	0.1948
SO_RS22090	SO_4750	-1.2221	0.0786	0.2453
SO_RS22095	SO_4751	-1.8738	0.0105	0.0944
SO_RS22100	SO_4752	-3.4555	0.0772	0.2438
SO_RS22105	SO_4753	-1.8094	0.0040	0.0600
SO_RS22110	SO_4754	-1.9778	0.0657	0.2276
SO_RS22115	SO_4755	0.3481	0.7159	0.8893
SO_RS22120	SO_4756	0.6845	0.5420	0.7456
SO_RS22125	SO_4757	-0.2091	0.9012	1.0000
SO_RS22130	SO_4758	-1.5431	0.0035	0.0572
SO_RS22135		-0.3986	0.7174	0.8902
SO_RS22140		-4.7760	0.0119	0.0991
SO_RS22145		-3.2353	0.2318	0.4530
SO_RS22150		-1.4527	0.1328	0.3263
SO_RS22155		-2.2386	0.0011	0.0328
SO_RS22160		3.8136	0.2352	0.4559
SO_RS22165		1.1090	0.4795	0.6966
SO_RS22170		0.0000	1.0000	1.0000
SO_RS22175		0.0000	1.0000	1.0000
SO_RS22180		0.0000	1.0000	1.0000
SO_RS22185		0.0000	1.0000	1.0000
SO_RS22190		2.9116	0.3666	0.5960
SO_RS22195		-5.0672	0.0689	0.2324
SO_RS22200		-0.9290	0.6101	0.8037
SO_RS22205		2.3199	0.0952	0.2732
SO_RS22210		0.0000	1.0000	1.0000

SO_RS10410	SO_2268	-1.9383	0.0052	0.0673
SO_RS10415	SO_2269	-2.3287	0.1701	0.3769
SO_RS10420	SO_2270	-1.1617	0.2063	0.4230
SO_RS10425	SO_2271	0.0000	1.0000	1.0000
SO_RS10435	SO_2274	1.2393	0.4339	0.6603
SO_RS10440	SO_2275	-1.4487	0.1712	0.3786
SO_RS10445	SO_2277	0.4241	0.3096	0.5360
SO_RS10450	SO_2278	0.8578	0.6417	0.8325
SO_RS10455	SO_2279	2.4465	0.0463	0.1878
SO_RS10460	SO_2280	2.7232	0.1105	0.2953
SO_RS10465	SO_2281	-2.6045	0.1417	0.3395
SO_RS10470	SO_2282	-1.0697	0.0612	0.2202
SO_RS10480		-0.1957	0.9415	1.0000
SO_RS10485		-2.3716	0.2909	0.5140
SO_RS10490	SO_2286	0.4665	0.4431	0.6665
SO_RS10495	SO_2288	7.6197	0.0088	0.0869
SO_RS10500	SO_2290	-1.0571	0.4442	0.6674
SO_RS10505		-8.7662	0.0098	0.0920
SO_RS10510	SO_2292	0.0000	1.0000	1.0000
SO_RS10525	SO_2295	0.6335	0.6764	0.8587
SO_RS10530	SO_2296	7.9529	0.0068	0.0772
SO_RS10540	SO_2298	0.0000	1.0000	1.0000
SO_RS10545	SO_2299	-2.2111	0.0014	0.0370
SO_RS10550	SO_2300	-0.0160	0.9718	1.0000
SO_RS10555	SO_2301	-1.9979	0.0654	0.2271
SO_RS10560	SO_2302	2.2343	0.0073	0.0791
SO_RS10565	SO_2303	0.9094	0.1727	0.3802
SO_RS10570	SO_2304	0.7914	0.1480	0.3468
SO_RS10575	SO_2305	-0.7798	0.2944	0.5170
SO_RS10580	SO_2306	-2.4324	0.0223	0.1286
SO_RS10585	SO_2307	0.9238	0.5736	0.7704
SO_RS10590	SO_2308	-2.0962	0.2592	0.4812
SO_RS10595	SO_2309	0.0000	1.0000	1.0000
SO_RS10600	SO_2310	-0.8554	0.0755	0.2420
SO_RS10605	SO_2312	5.1423	0.0000	0.0012
SO_RS10615		5.3198	0.0390	0.1722
SO_RS10625	SO_2318	0.0000	1.0000	1.0000
SO_RS10630	SO_2319	0.0000	1.0000	1.0000
SO_RS10635		6.3012	0.0240	0.1306
SO_RS10645		-6.4680	0.0347	0.1585
SO_RS10650	SO_2323	1.1465	0.5516	0.7528
SO_RS10655	SO_2324	5.3174	0.0987	0.2781
SO_RS10660	SO_2325	6.7521	0.0629	0.2226
SO_RS10665	SO_2326	0.0000	1.0000	1.0000
SO_RS10670	SO_2327	1.8420	0.3942	0.6227
SO_RS10675	SO_2328	2.2477	0.0191	0.1206
SO_RS10680	SO_2329	6.3653	0.0197	0.1217
SO_RS10685	SO_2330	0.8436	0.2987	0.5225
SO_RS10690	SO_2331	0.0000	1.0000	1.0000
SO_RS10695	SO_2332	0.0000	1.0000	1.0000
SO_RS10700	SO_2333	2.0259	0.1081	0.2907
SO_RS10705	SO_2335	2.0427	0.0540	0.2061
SO_RS10710	SO_2336	0.1339	0.7841	0.9422
SO_RS10715	SO_2337	-6.4993	0.0011	0.0328
SO_RS10720	SO_2338	-4.0486	0.0139	0.1069
SO_RS10725	SO_2339	-0.2410	0.7988	0.9511
SO_RS10730	SO_2340	-0.9066	0.0967	0.2752
SO_RS10735	SO_2341	-1.1130	0.0278	0.1428
SO_RS10740	SO_2342	2.4327	0.0012	0.0348
SO_RS10745	SO_2344	9.7214	0.0035	0.0572
SO_RS10750	SO_2345	0.8740	0.2707	0.4926
SO_RS10755	SO_2346	2.0609	0.1489	0.3482
SO_RS10760	SO_2347	1.2125	0.0135	0.1055
SO_RS10780	SO_2350	1.5467	0.2728	0.4948
SO_RS10785	SO_2351	-0.5944	0.5972	0.7926
SO_RS10790	SO_2352	-10.5997	0.0006	0.0238
SO_RS10795	SO_2353	-0.5684	0.6481	0.8389
SO_RS10800	SO_2354	-2.1892	0.1001	0.2801

SO_RS22215		-2.7737	0.0027	0.0502
SO_RS22220		1.5951	0.3293	0.5581
SO_RS22225		-0.7767	0.3935	0.6222
SO_RS22230		-1.0476	0.0718	0.2371
SO_RS22235		-1.0078	0.5809	0.7774
SO_RS22240		-5.4216	0.0687	0.2324
SO_RS22245		0.4933	0.5237	0.7321
SO_RS22250		-1.3752	0.1014	0.2828
SO_RS22260		-1.0275	0.5464	0.7486
SO_RS22270		2.9535	0.0347	0.1585
SO_RS22275		3.0913	0.1821	0.3917
SO_RS22280		3.1883	0.0015	0.0380
SO_RS22285		7.2297	0.0197	0.1217
SO_RS22290		3.8037	0.2014	0.4153
SO_RS22295		0.0000	1.0000	1.0000
SO_RS22300		1.3411	0.5452	0.7484
SO_RS22310		2.9660	0.1065	0.2890
SO_RS22315		-3.5809	0.0236	0.1304
SO_RS22320		-1.2795	0.5214	0.7309
SO_RS22325		3.8085	0.1478	0.3467
SO_RS22330		-2.2694	0.2328	0.4533
SO_RS22335		2.9551	0.1111	0.2959
SO_RS22340		-1.4430	0.0650	0.2260
SO_RS22345		-0.4350	0.5787	0.7751
SO_RS22350		-1.0894	0.1824	0.3919
SO_RS22355		-1.4093	0.0017	0.0422
SO_RS22360		-0.5346	0.3854	0.6139
SO_RS22365		4.7545	0.1074	0.2903
SO_RS22370		5.9801	0.1155	0.3033
SO_RS22380		0.0000	1.0000	1.0000
SO_RS22385		1.1240	0.5667	0.7659
SO_RS22390		-0.0865	0.9233	1.0000
SO_RS22395		-7.9425	0.0181	0.1196
SO_RS22400		0.7972	0.4295	0.6580
SO_RS22405		-2.1658	0.0088	0.0869
SO_RS22410		0.6028	0.6423	0.8329
SO_RS22415		-9.1398	0.0124	0.1014
SO_RS22420		0.0000	1.0000	1.0000
SO_RS22430		1.2972	0.2098	0.4273
SO_RS22435		0.4285	0.6433	0.8339
SO_RS22440		0.3916	0.7108	0.8845
SO_RS22445		-0.1065	0.9270	1.0000
SO_RS22450		-0.4341	0.4579	0.6784
SO_RS22455		-0.8451	0.2704	0.4926
SO_RS22460		-0.1744	0.8849	1.0000
SO_RS22465		-5.0709	0.0593	0.2170
SO_RS22475		-5.3147	0.0145	0.1094
SO_RS22480		-8.1960	0.0189	0.1205
SO_RS22485		-0.9683	0.3272	0.5560
SO_RS22490		-3.0633	0.0188	0.1205
SO_RS22495		-1.2584	0.4972	0.7116
SO_RS22500		-0.1610	0.8887	1.0000
SO_RS22505		0.0000	1.0000	1.0000
SO_RS22510		-5.6231	0.0350	0.1594
SO_RS22520		-4.4281	0.0109	0.0960
SO_RS22525		-3.2962	0.0206	0.1255
SO_RS22530		-1.8651	0.0761	0.2423
SO_RS22535		-2.5227	0.0233	0.1302
SO_RS22540		-3.2792	0.1559	0.3572
SO_RS22545		-5.8365	0.0395	0.1731
SO_RS22550		-1.1185	0.4860	0.7015
SO_RS22560		-2.9344	0.0086	0.0868
SO_RS22565		-0.5012	0.7854	0.9423
SO_RS22570		-1.5609	0.0222	0.1284
SO_RS22575		-0.1806	0.7370	0.9041
SO_RS22580		7.2665	0.0482	0.1919
SO_RS22585		-2.0766	0.3348	0.5631
SO_RS22590		-0.0915	0.8621	0.9970

SO_RS10805	SO_2355	3.1344	0.0002	0.0149
SO_RS10810	SO_2356	4.8466	0.0037	0.0583
SO_RS10815	SO_2357	4.3528	0.1879	0.3999
SO_RS10820	SO_2358	0.0000	1.0000	1.0000
SO_RS10825	SO_2359	0.1726	0.9152	1.0000
SO_RS10830	SO_2360	1.0149	0.5762	0.7722
SO_RS10835	SO_2361	1.1773	0.0187	0.1205
SO_RS10840	SO_2362	-0.1829	0.9349	1.0000
SO_RS10845	SO_2363	0.5844	0.4474	0.6693
SO_RS10850	SO_2364	0.2743	0.5723	0.7693
SO_RS10855	SO_2365	-0.7980	0.6544	0.8426
SO_RS10860	SO_2366	-0.4447	0.4118	0.6405
SO_RS10865		-1.6491	0.4620	0.6818
SO_RS10875		-0.2848	0.8916	1.0000
SO_RS10885	SO_2373	1.2575	0.3914	0.6204
SO_RS10890	SO_2374	-1.7308	0.1350	0.3302
SO_RS10895	SO_2375	-0.5869	0.0856	0.2571
SO_RS10900	SO_2376	3.5252	0.0772	0.2438
SO_RS10905	SO_2377	-1.3497	0.4470	0.6693
SO_RS10910	SO_2378	0.0000	1.0000	1.0000
SO_RS10915	SO_2379	2.0989	0.3415	0.5696
SO_RS10920	SO_2380	-0.7056	0.6826	0.8640
SO_RS10925	SO_2381	0.0000	1.0000	1.0000
SO_RS10930	SO_2383	0.0000	1.0000	1.0000
SO_RS10935	SO_2384	0.0000	1.0000	1.0000
SO_RS10940	SO_2385	-0.6481	0.7181	0.8904
SO_RS10945	SO_2387	-7.6826	0.0169	0.1172
SO_RS10950	SO_2388	1.9022	0.3026	0.5274
SO_RS10955	SO_2389	0.0000	1.0000	1.0000
SO_RS10960	SO_2390	-0.0805	0.9420	1.0000
SO_RS10965	SO_2391	-3.6403	0.0684	0.2320
SO_RS10970	SO_2392	3.7910	0.1346	0.3295
SO_RS10975	SO_2394	-6.5601	0.0085	0.0868
SO_RS10980	SO_2395	1.8330	0.0612	0.2202
SO_RS10985	SO_2396	2.2469	0.1515	0.3520
SO_RS10990	SO_2397	-1.3930	0.2696	0.4919
SO_RS10995	SO_2398	-2.0825	0.2760	0.4983
SO_RS11000	SO_2399	0.3458	0.8424	0.9808
SO_RS11005	SO_2400	0.0000	1.0000	1.0000
SO_RS11010	SO_2401	-1.2077	0.0476	0.1912
SO_RS11015	SO_2402	-0.0570	0.8380	0.9779
SO_RS11020	SO_2403	-1.3460	0.0991	0.2790
SO_RS11025	SO_2404	0.9883	0.2568	0.4777
SO_RS11030	SO_2406	3.3885	0.0039	0.0596
SO_RS11035	SO_2407	-1.2863	0.4630	0.6822
SO_RS11040	SO_2408	0.0000	1.0000	1.0000
SO_RS11045	SO_2409	-0.9281	0.6029	0.7973
SO_RS11050	SO_2410	1.4615	0.2330	0.4533
SO_RS11055	SO_2411	0.5185	0.2010	0.4149
SO_RS11065	SO_2413	6.4675	0.0264	0.1382
SO_RS11070	SO_2414	-4.0114	0.0281	0.1436
SO_RS11075	SO_2415	-0.3833	0.6041	0.7983
SO_RS11080	SO_2416	-0.1419	0.8888	1.0000
SO_RS11085	SO_2417	-4.5054	0.0497	0.1948
SO_RS11090	SO_2418	-0.3215	0.8531	0.9893
SO_RS11095	SO_2419	-0.5224	0.4927	0.7066
SO_RS11100	SO_2420	1.4513	0.3321	0.5603
SO_RS11105	SO_2421	2.6269	0.0037	0.0583
SO_RS11110	SO_2422	3.8033	0.2292	0.4506
SO_RS11115	SO_2423	1.0555	0.5413	0.7453
SO_RS11120	SO_2424	2.0143	0.0169	0.1172
SO_RS11125	SO_2426	-2.8252	0.0070	0.0779
SO_RS11130	SO_2427	2.5432	0.0000	0.0031
SO_RS11135	SO_2429	-0.2756	0.8727	1.0000
SO_RS11140	SO_2430	8.5698	0.0085	0.0868
SO_RS11145	SO_2431	-2.8252	0.0863	0.2585
SO_RS11150	SO_2432	2.8932	0.1661	0.3720
SO_RS11155	SO_2433	-0.0250	0.9598	1.0000

SO_RS22595		0.2909	0.6752	0.8576
SO_RS22600		-2.2880	0.2615	0.4829
SO_RS22615		-1.5056	0.0890	0.2632
SO_RS22620		-0.9063	0.0550	0.2078
SO_RS22625		0.0000	1.0000	1.0000
SO_RS22630		0.0000	1.0000	1.0000
SO_RS22635		0.0000	1.0000	1.0000
SO_RS22640		1.7524	0.0005	0.0214
SO_RS22645		-0.4494	0.1633	0.3679
SO_RS22650		0.0000	1.0000	1.0000
SO_RS22665		0.0000	1.0000	1.0000
SO_RS22675		-4.3456	0.0024	0.0485
SO_RS22680		1.1485	0.4428	0.6665
SO_RS22695		0.0000	1.0000	1.0000
SO_RS22700		-1.1534	0.3829	0.6112
SO_RS22710		-1.7863	0.2924	0.5156
SO_RS22715		-4.2930	0.1054	0.2880
SO_RS22720		7.6646	0.0078	0.0830
SO_RS22725		-2.3865	0.0214	0.1272
SO_RS22730		-0.8033	0.5547	0.7561
SO_RS22735		-0.9220	0.6388	0.8305
SO_RS22740		0.1129	0.9208	1.0000
SO_RS22745		-3.3742	0.0094	0.0899
SO_RS22750		-5.5556	0.0081	0.0845
SO_RS22755		-1.2795	0.2064	0.4231
SO_RS22765		-5.9462	0.0313	0.1497
SO_RS22770		-1.3004	0.0758	0.2422
SO_RS22775		-1.3455	0.0563	0.2106
SO_RS22780		0.6091	0.3774	0.6068
SO_RS22785		-2.2676	0.0112	0.0981
SO_RS22795		6.6623	0.1234	0.3141
SO_RS22805		-1.7261	0.5180	0.7285
SO_RS22810		-3.1695	0.0483	0.1921
SO_RS22815		3.9360	0.0882	0.2620
SO_RS22820		0.9120	0.2129	0.4315
SO_RS22830		-2.9230	0.0438	0.1814
SO_RS22835		0.0000	1.0000	1.0000
SO_RS22840		1.8159	0.0150	0.1106
SO_RS22845		4.0032	0.0025	0.0490
SO_RS22850		8.4554	0.0109	0.0960
SO_RS22855		0.0195	0.9724	1.0000
SO_RS22860		2.1885	0.0260	0.1373
SO_RS22865		-0.1306	0.8654	0.9990
SO_RS22870		-0.9874	0.5552	0.7563
SO_RS22875		-0.7456	0.2455	0.4673
SO_RS22880		0.4259	0.2568	0.4777
SO_RS22890		3.9782	0.1066	0.2890
SO_RS22895		-2.9645	0.1899	0.4026
SO_RS22900		-0.7347	0.6778	0.8599
SO_RS22905		-0.0121	0.9904	1.0000
SO_RS22935		-0.2875	0.6811	0.8626
SO_RS22940		2.0859	0.4460	0.6686
SO_RS22950	SO_0543	-0.8725	0.4662	0.6843
SO_RS22960	SO_0755	-1.3116	0.3775	0.6068
SO_RS22965	SO_0844	-3.2347	0.1631	0.3679
SO_RS22985	SO_1869	0.0000	1.0000	1.0000
SO_RS22990	SO_1984	-2.2006	0.3308	0.5595
SO_RS23000	SO_2273	2.9116	0.4111	0.6399
SO_RS23015	SO_2393	0.0000	1.0000	1.0000
SO_RS23030		2.9043	0.3275	0.5562
SO_RS23035	SO_2999	0.0000	1.0000	1.0000
SO_RS23065	SO_4430	0.0000	1.0000	1.0000
SO_RS23070	SO_4317	0.5207	0.1725	0.3802
SO_RS23085	SO_2704	5.9786	0.0452	0.1850
SO_RS23090	SO_2944	0.0277	0.9808	1.0000
SO_RS23100		0.0000	1.0000	1.0000
SO_RS23120	SO_4770	-1.9569	0.4237	0.6524
SO_RS23130		0.0000	1.0000	1.0000

SO_RS11160	SO_2434	-0.8187	0.5293	0.7353
SO_RS11165	SO_2435	0.3359	0.7165	0.8899
SO_RS11170	SO_2436	-2.3610	0.0116	0.0988
SO_RS11175	SO_2437	0.5492	0.6645	0.8493
SO_RS11180	SO_2438	2.7755	0.0782	0.2449
SO_RS11185	SO_2439	0.5589	0.2069	0.4236
SO_RS11190	SO_4782	0.5023	0.4475	0.6693
SO_RS11195	SO_2440	2.0105	0.2322	0.4533
SO_RS11200	SO_2441	-0.8823	0.5654	0.7649
SO_RS11205	SO_2442	0.0000	1.0000	1.0000
SO_RS11210	SO_2443	2.0909	0.4420	0.6664
SO_RS11215	SO_2444	5.3266	0.0665	0.2293
SO_RS11220	SO_2445	-3.3381	0.1534	0.3532
SO_RS11225	SO_2446	-2.6230	0.1768	0.3857
SO_RS11230	SO_2447	2.9517	0.1075	0.2903
SO_RS11235	SO_2448	5.4287	0.1128	0.2995
SO_RS11240	SO_2449	-1.5314	0.4430	0.6665
SO_RS11245	SO_2450	0.1984	0.9222	1.0000
SO_RS11250	SO_2451	-3.8893	0.0451	0.1850
SO_RS11255	SO_2452	1.4397	0.4197	0.6482
SO_RS11260	SO_2453	3.0371	0.0855	0.2571
SO_RS11265	SO_2454	5.1877	0.0064	0.0762
SO_RS11270	SO_2455	0.3841	0.6737	0.8565
SO_RS11280	SO_2457	3.7819	0.1812	0.3903
SO_RS11285	SO_2458	-4.4249	0.0117	0.0988
SO_RS11290	SO_2459	7.2269	0.0577	0.2142
SO_RS11295	SO_2460	2.0908	0.4473	0.6693
SO_RS11300		-7.3093	0.0194	0.1216
SO_RS11310		-1.9743	0.1503	0.3507
SO_RS11315		-0.7764	0.4759	0.6932
SO_RS11320	SO_2467	-4.2217	0.0498	0.1948
SO_RS11330	SO_2469	-1.7159	0.0547	0.2076
SO_RS11335	SO_2470	-0.2750	0.8363	0.9768
SO_RS11340	SO_2471	8.3631	0.0029	0.0512
SO_RS11345	SO_2472	-0.1833	0.9303	1.0000
SO_RS11350	SO_2473	2.4911	0.2707	0.4926
SO_RS11355	SO_2474	-1.2708	0.3196	0.5478
SO_RS11360	SO_2476	-1.0232	0.1170	0.3057
SO_RS11365	SO_2477	-0.1344	0.8184	0.9646
SO_RS11370	SO_2478	7.7543	0.0062	0.0742
SO_RS11375	SO_2479	2.9080	0.3346	0.5630
SO_RS11380	SO_2480	9.4377	0.0021	0.0459
SO_RS11385	SO_2481	-0.3928	0.6970	0.8743
SO_RS11390	SO_2483	-0.4192	0.6053	0.7987
SO_RS11395	SO_2484	7.1954	0.0594	0.2170
SO_RS11400	SO_2485	11.1874	0.0000	0.0052
SO_RS11405	SO_2486	1.3709	0.3367	0.5647
SO_RS11410	SO_2487	0.3467	0.4599	0.6799
SO_RS11415	SO_2488	1.7133	0.1505	0.3507
SO_RS11420	SO_2489	0.5712	0.5231	0.7318
SO_RS11425	SO_2490	-1.0215	0.2851	0.5089
SO_RS11430	SO_2491	0.2746	0.7062	0.8813
SO_RS11435	SO_2492	0.1801	0.6518	0.8408
SO_RS11440	SO_2493	2.2378	0.0900	0.2640
SO_RS11445	SO_2494	-0.3613	0.8579	0.9931
SO_RS11450	SO_2495	-0.6854	0.4073	0.6369
SO_RS11455	SO_4783	5.9779	0.0560	0.2105
SO_RS11460	SO_2497	5.3301	0.1454	0.3437
SO_RS11465	SO_2498	-0.7523	0.3983	0.6275
SO_RS11470	SO_2499	-2.0449	0.2770	0.4994
SO_RS11475	SO_2500	-0.9512	0.2083	0.4257
SO_RS11495	SO_2501	-2.6457	0.2693	0.4919
SO_RS11500	SO_2503	-1.1032	0.4498	0.6703
SO_RS11505	SO_2504	-8.7265	0.0149	0.1102
SO_RS11510	SO_2506	-0.6438	0.4140	0.6418
SO_RS11540	SO_2507	-2.4644	0.0090	0.0874
SO_RS11545	SO_2508	10.1304	0.0009	0.0304
SO_RS11550	SO_2509	3.8033	0.2151	0.4338

SO_RS23135		-4.1346	0.1284	0.3201
SO_RS23140		0.6455	0.2345	0.4550
SO_RS23150	SO_4820	3.4228	0.2527	0.4738
SO_RS23175		0.0000	1.0000	1.0000
SO_RS23180		-7.0802	0.0196	0.1217
SO_RS23185	SO_4759	-3.3363	0.2281	0.4500
SO_RS23190		3.6312	0.1547	0.3551
SO_RS23205		4.1153	0.2044	0.4200
SO_RS23215		3.4319	0.2891	0.5114
SO_RS23225		0.0000	1.0000	1.0000
SO_RS23230	SO_4819	-3.7504	0.1055	0.2880
SO_RS23235	SO_2468	0.0000	1.0000	1.0000
SO_RS23245		-1.9208	0.4270	0.6559
SO_RS23250		0.0000	1.0000	1.0000
SO_RS23255		-8.3043	0.0229	0.1291
SO_RS23260		0.1182	0.8765	1.0000
SO_RS23270	SO_3510	4.1130	0.2325	0.4533
SO_RS23280		1.5689	0.3719	0.6014
SO_RS23285		2.0907	0.5327	0.7386
SO_RS23290		0.0000	1.0000	1.0000
SO_RS23295		0.0000	1.0000	1.0000
SO_RS23300		-0.4318	0.8537	0.9896
SO_RS23310		0.0000	1.0000	1.0000
SO_RS23315	SO_4353	0.0000	1.0000	1.0000
SO_RS23325	SO_4412	2.0908	0.4590	0.6788
SO_RS23330	SO_4493	-3.1185	0.1360	0.3314
SO_RS23335		0.0000	1.0000	1.0000
SO_RS23340		0.0000	1.0000	1.0000
SO_RS23345		0.0000	1.0000	1.0000
SO_RS23350		-4.1346	0.1457	0.3439
