# Supplementary material for: Elucidating the physiological significance of iodate reduction in Shewanella species
Source: mLife. 2026 Aug 23;5(4):507–19. doi: 10.1002/mlf2.70096 (PMC13501091; doi:10.1002/mlf2.70096)
Supplement: Supplementary file 2 [file MLF2-5-507-s002.docx]

**Elucidating the physiological significance of iodate reduction in *Shewanella* species**

Ziyi Wang ^a^, Lingyu Hou ^a^, Yidan Hu ^a^, Zhou Jiang ^a^, Yiran Dong ^a,b^, Liang Shi ^a,b^, and Yongguang Jiang ^a,c#^

^a^ Department of Biological Sciences and Technology, School of Environmental Studies, China University of Geosciences, Wuhan, Hubei 430074, PR China

^b^ State Key Laboratory of Geomicrobiology and Environment, China University of Geosciences, Wuhan, Hubei 430078, PR China

^c^ Hubei Key Laboratory of Wetland Evolution & Eco-Restoration, Wuhan, Hubei 430078, PR China

6 figures

5 tables

12 pages

Figure S1 Continuous reduction of $\mathrm{IO}_{3}^{-}$ by *S. oneidensis* MR-1. One hundred percent of $\mathrm{IO}_{3}^{-}$ was equal to 0.25 mM. At 36 h, $\mathrm{IO}_{3}^{-}$ was supplemented to its initial concentration. Arrows indicate the time points when RNA samples were collected.

Figure S2 Growth of *S. oneidensis* MR-1 with fumarate as the terminal electron acceptor. (A) Optical density of bacterial cultures at 600 nm. (B) Concentration of total cellular protein. M1, M1 medium; LB, LB medium.

Figure S3 Transcriptomic analyses of *S. oneidensis* MR-1 cells during $\mathrm{IO}_{3}^{-}$ reduction. (A) The transcriptome heatmaps of differentially expressed genes between the fast $\mathrm{IO}_{3}^{-}$-reduction phase and the initial phase. (B) Volcano plot of differentially expressed genes. The differences were considered statistically significant when *p* value < 0.05, False Discovery Rate (FDR) < 0.05 and |log_2_Fold change| > 1.

Figure S4 Growth of mutants and wild type of *S. oneidensis* MR-1. (A) Optical density of bacterial cultures at 600 nm under aerobic conditions. (B) Optical density of bacterial cultures at 600 nm under fumarate-respiring conditions. (C) Optical density of bacterial cultures at 600 nm under DMSO-respiring conditions. Lactate was used as the electron donor.

Figure S5 $\mathrm{IO}_{3}^{-}$ reduction by complemented strains, mutants and wild type of *S. oneidensis* MR-1. (A) $\mathrm{IO}_{3}^{-}$ reduction by Δ*so0534* and complemented strains. (B) $\mathrm{IO}_{3}^{-}$ reduction by Δ*so1563* and complemented strains. (C) $\mathrm{IO}_{3}^{-}$ reduction by Δ*so1659* and complemented strains. One hundred percent of $\mathrm{IO}_{3}^{-}$ was equal to 0.25 mM. For points with no error bar, the error was smaller than the size of the symbol. WT, wild type; EV, empty vector.

Figure S6 Concentration of H_2_O_2_ over 60 minutes of $\mathrm{IO}_{3}^{-}$ reduction. (A) Traces of H_2_O_2_ concentration during $\mathrm{IO}_{3}^{-}$ reduction. (B) Maximum values of H_2_O_2_ concentration in the culture of each strain. WT, wild type. Statistical method: Student's *t*-test. ns, *p* > 0.05; ***, *p* < 0.001. The Δ*mtrCAB* strain was used as a positive control (Guo et al., 2022).

**Table S1** Bacterial strains and plasmids used in this study

| **Strains or plasmids** | **Relevant genotype or uses** | **Source or reference** |
| --- | --- | --- |
| **Strains** | | |
| *Shewanella oneidensis* MR-1 | | |
| WT | Wild type | Lab stock |
| Δ*mtrCAB* | *mtrCAB* deletion mutant of MR-1 | (20) |
| Δ*dmsEFAB* | *dmsEFAB* deletion mutant of MR-1 | (20) |
| Δ*so1563* | *so1563* deletion mutant of MR-1 | This study |
| Δ*so1659* | *so1659* deletion mutant of MR-1 | This study |
| Δ*so0534* | *so0534* deletion mutant of MR-1 | This study |
| Δ*so2052* | *so2052* deletion mutant of MR-1 | This study |
| Δ*so3420* | *so3420* deletion mutant of MR-1 | This study |
| *Shewanella livingstonensis* | CCTCC AB2020017 | China Center for Type Culture Collection |
| *Shewanella piezotolerans* | MCCC 1A01966 | Marine Culture Collection of China |
| *Escherichia coli* |  |  |
| WM3064 | Donor strain for conjugation, Δ*dapA* | Lab stock |
| DH5α | *E. coli* host for cloning | Takara Co., Ltd. |
| **Plasmids** |  |  |
| pDS3.2 | Suicide vector, Gm^r^ sacB, with multiple cloning sites | (27) |
| pBBR1MCS-2 | Expression vector, Kmr Plac | Lab stock |
| p*so1563* | Recombinant pBBR1MCS-2 containing *so1563* coding sequence | This study |
| p*so1659* | Recombinant pBBR1MCS-2 containing *so1659* coding sequence | This study |
| p*so0534* | Recombinant pBBR1MCS-2 containing *so0534* coding sequence | This study |
| p*so2052* | Recombinant pBBR1MCS-2 containing *so2052* coding sequence | This study |
| p*so3420* | Recombinant pBBR1MCS-2 containing *so3420* coding sequence | This study |

**Table S2** Transcriptional changes of genes during the fast iodate-reduction phase relative to the initial phase. FC, fold change; FDR, false discovery rate. Statistical method: Exact test (edgeR). (uploaded separately)

**Table S3** Transcriptional changes of extracellular electron transfer genes in transcriptome during the fast iodate-reduction phase relative to the initial phase. FC, fold change. Statistical method: Exact test (edgeR).

| **Gene name** | **Locus tag** | **Log_2_FC** | ***p* value** | **Description** |
| --- | --- | --- | --- | --- |
| *dmsE* | SO_1427 | − 2.49 | 0.01 | DmsE family decaheme c-type cytochrome |
| *dmsF* | SO_1428 | 0.50 | 0.49 | MtrB/PioB family decaheme-associated outer membrane protein |
| *dmsA* | SO_1429 | 1.60 | 0.06 | Molybdopterin-dependent oxidoreductase |
| *dmsB* | SO_1430 | 1.32 | 0.04 | Dimethylsulfoxide reductase subunit B |
| *dmsG* | SO_1431 | − 3.53 | 0.10 | Molecular chaperone TorD |
| *dmsH* | SO_1432 | − 3.93 | 0.07 | Hypothetical protein |
| *so4362* | SO_4362 | 0.00 | 1.00 | Oxidoreductase chaperone protein |
| *so4361* | SO_4361 | 0.00 | 1.00 | Oxidoreductase associated protein |
| *so4360* | SO_4360 | 0.00 | 1.00 | DmsE family decaheme c-type cytochrome |
| *so4359* | SO_4359 | − 7.89 | 0.00 | MtrB/PioB family decaheme-associated outer membrane protein |
| *so4358* | SO_4358 | − 0.09 | 0.95 | Dimethyl sulfoxide reductase subunit A |
| *so4357* | SO_4357 | 3.40 | 0.10 | Dimethylsulfoxide reductase subunit B |
| *mtrD* | SO_1782 | 5.21 | 0.12 | DmsE family decaheme c-type cytochrome |
| *mtrE* | SO_1781 | − 2.71 | 0.18 | MtrB/PioB family decaheme-associated outer membrane protein |
| *mtrF* | SO_1780 | − 1.47 | 0.27 | OmcA/MtrC family decaheme c-type cytochrome |
| *omcA* | SO_1779 | 1.62 | 0.00 | OmcA/MtrC family decaheme c-type cytochrome |
| *mtrC* | SO_1778 | 1.44 | 0.01 | OmcA/MtrC family decaheme c-type cytochrome |
| *mtrA* | SO_1777 | 0.42 | 0.34 | DmsE family decaheme c-type cytochrome |
| *mtrB* | SO_1776 | 2.28 | 0.00 | MtrB/PioB family decaheme-associated outer membrane protein |
| *cymA* | SO_4591 | − 0.29 | 0.52 | Cytochrome c |
| *cctA* | SO_2727 | − 0.25 | 0.70 | Cytochrome c3 family protein |
| *fccA* | SO_0970 | 2.31 | 0.00 | Flavocytochrome c |

**Table S4** Transcriptional changes of eighteen genes during the fast iodate-reduction phase relative to the initial phase. FC, fold change. SD, standard deviation. Statistical method: Exact test (edgeR) for transcriptomic analysis; Student's *t*‐test for qRT-PCR. Asterisks represent levels of statistical difference. *, 0.01 < *p* < 0.05; **, 0.001 < *p* < 0.01; ***, *p* < 0.001.

| **Gene name** | **Locus tag** | **Transcriptome** | **qRT-PCR** | **Description** |
| --- | --- | --- | --- | --- |
|  |  | **log_2_FC** | **Ratio of relative expression**  **(Mean + SD)** |  |
| *so0091* | SO_0091 | 4.10 | 0.18 ± 0.05 * | Cyclic nucleotide binding domain protein |
| *arsC2* | SO_0533 | 4.10 | 0.95 ± 0.07 | Thioredoxin-coupled cytoplasmic arsenate reductase ArsC2 |
| ***so0534*** | SO_0534 | 2.10 | 1.65 ± 0.16 * | Arsenate permease ACR3 family |
| *so0939* | SO_0939 | 3.43 | 1.47 ± 0.27 | Split-Soret Diheme cytochrome C |
| ***ahpC*** | SO_0958 | 1.20 | 1.26 ± 0.00 * | Alkyl hydroperoxide reductase peroxiredoxin component AhpC |
| *so1302* | SO_1302 | 8.84 ** | 0.70 ± 0.14 | Chloride channel protein ClC family |
| ***so1563*** | SO_1563 | 7.02 *** | 2.78 ± 0.12 ** | Cytoplasmic glutathione peroxidase, *cgpD* |
| ***so1659*** | SO_1659 | 2.26 *** | 2.53 ± 0.35 * | Surface localized decaheme cytochrome c lipoprotein |
| ***so2052*** | SO_2052 | 5.97 ** | 10.55 ± 0.62 *** | FAD-dependent oxidoreductase |
| *ccpA* | SO_2178 | 3.65 * | 1.12 ± 0.10 | Diheme cytochrome c5 peroxidase CcpA |
| *so2519* | SO_2519 | 6.89 | 0.59 ± 0.07 ** | Transcriptional regulator AraC family |
| ***tsaA*** | SO_2756 | 2.73 *** | 3.77 ± 0.46 ** | Peroxiredoxin TsaA |
| *arsC* | SO_2871 | 7.13 | 0.45 ± 0.01 * | Glutaredoxin-dependent arsenate reductase ArsC |
| ***sodB*** | SO_2881 | 1.55 * | 3.27 ± 0.12 ** | Fe/Mn superoxide dismutase SodB |
| *so3058* | SO_3058 | 11.06 ** | 0.22 ± 0.01 | Flavocytochrome c flavin subunit |
| ***so3420*** | SO_3420 | 11.51 *** | 3.21 ± 0.13 * | Monoheme cytochrome c' |
| *otr* | SO_4144 | 8.04 * | 0.43 ± 0.04 | Octaheme tetrathionate reductase Otr |
| ***katG*** | SO_4405 | 1.70 | 2.67 ± 0.00 ** | Catalase/peroxidase HPI |

**Table S5** Primers used in this study

| **Primer name** | **Sequences (5’- 3’)** | **Usage** |
| --- | --- | --- |
| so1563_5O | CATGCATGCAAGGCGGGAAATCTGTTATT | Construction of Δ*so1563* |
| so1563_5I | AAAATACGCAAGACACGGATAAGTATCCAGACGTCGATTA |  |
| so1563_3O | TTCATAGGTCTGCAGCTAATACTGGCGGTGTTTACAATCAA |  |
| so1563_3I | TCCGAGCTCAACGCTCGTCTAAAGGTAAG |  |
| so1563_F | CTTTTCGTGAACTTATCGCC |  |
| so1563_R | TTAAACTCAGCACAGTCACA |  |
| so1563_PF | CCGGAATTC*GAAAGAGGAGAAA*TACTAGATGACATCCACTATCTATCGC | Construction of p *so1563* |
| so1563_PR | TGCTCTAGATTACTTATTTAAAAGTGACTCTATTTTGTC |  |
| so1659_5O | CATGCATGC ACTCAGGGTGTTGAGACTAT | Construction of Δ*so1659* |
| so1659_5I | TATTAGCTGCAGACCTATGAATAGAAGTAACGGTGCGATTG |  |
| so1659_3O | TTCATAGGTCTGCAGCTAATACGGTTAAACACAAGGCACTA |  |
| so1659_3I | TCCGAGCTCTTCTCATCGAAAAACCACGA |  |
| so1659_F | CTGACTTATTTTCATCGGCG |  |
| so1659_R | ATCGATATGCACACCGTAAA |  |
| so1659_PF | CCGGAATTC*GAAAGAGGAGAAA*TACTAGATGATGAAAAACTACAATAAATCTCTA | Construction of p*so1659* |
| so1659_PR | TGCTCTAGACTAGTGTGCTACCTCTGC |  |
| so0534_5O | CGTTGCATGCGGGATGGCTGCTAAAGA | Construction of Δ*so0534* |
| so0534_5I | gactggcttaggtcgtctctAAACAGGGCGATGACTAA |  |
| so0534_3I | agagacgacctaagccagtcATTATCTCCCAGCCACAAA |  |
| so0534_3O | AGTTGAGCTCTCATACCTACCGCCACAT |  |
| so0534_F | GCCCTTGCCGATGAGAC |  |
| so0534_R | CGCCAGCAGACTTTCCA |  |
| so0534_PF | CCGGAATTC*GAAAGAGGAGAAA*TACTAGATGGGAATTTTTGAGCGTT | Construction of p*so0534* |
| so0534_PR | TGCTCTAGATTAAGATTGAAACCAGTGCC |  |
| so2052_5O | CATGCATGCATTGGTGGCAAAATAAACCG | Construction of Δ*so2052* |
| so2052_5I | TATTAGCTGCAGACCTATGAATTTTCAGACAATTCAACCGC |  |
| so2052_3O | TTCATAGGTCTGCAGCTAATAATCGATGGTCAAATGCAAGA |  |
| so2052_3I | TCCGAGCTCATTCGACTAGGTAAACTCGC |  |
| so2052_F | GATGACCTTGCAGTAAACAC |  |
| so2052_R | CTTTATGGTGCAGTATCCCA |  |
| so3420_5O | AGCCCTGCAGGCCACTCCCCAGTTTTAATCA | Construction of Δ*so3420* |
| so3420_5I | TATTAGCTGCAGACCTATGAACTGAATCCAGATTGGCGATA |  |
| so3420_3O | TTCATAGGTCTGCAGCTAATACTACCGTAGCTCAAAAAGGT |  |
| so3420_3I | TCCGAGCTCCCGATACCAGTACTTTCCTG |  |
| so3420_F | TTCTCTGCCTTAGCCATTTT |  |
| so3420_R | TTATCTGTGGTACTAGGGCA |  |
| pBBR-F | TTGGGTAACGCCAGGGTTTT | Detection of pBBR1MCS-2 |
| pBBR-R | TGTGTGGAATTGTGAGCGGA |  |
| ahpC_Fq | GCTGATAGTGCCTGTTGGGT | Quantitative detection of *ahpC* |
| ahpC_Rq | CGCCAAACTACAAGGCATGG |  |
| arsC_Fq | CGCGAAACCTTAGCCCTACT | Quantitative detection of *arsC* |
| arsC_Rq | GGCACTGAGTTGCAGTTTGG |  |
| arsC2_Fq | CGAAGCATTTTAGCGGAGGC | Quantitative detection of *arsC2* |
| arsC2_Rq | TCATGGGATGAACCTTGCCC |  |
| ccpA_Fq | TTGCCCAGTTCCACCTTCTC | Quantitative detection of *ccpA* |
| ccpA_Rq | ACCCTCACGGCTATATTCGC |  |
| katG_Fq | TAATCGACGCTCGGGATTGG | Quantitative detection of *katG* |
| katG_Rq | GGCGATGAAGATAGACCCCG |  |
| otr_Fq | TAGCTGTCACGCTGGCTATG | Quantitative detection of *otr* |
| otr_Rq | TCGGGATAGGCCATGGAAGA |  |
| so0091_Fq | TCGCCCTTCTTGGATGAGTT | Quantitative detection of *so0091* |
| so0091_Rq | GCCAGCGAAGAACAATTTAAGC |  |
| so0534_Fq | TGGTATTTGCCGACTGGGTT | Quantitative detection of *so0534* |
| so0534_Rq | GTTGGCTCCACACAAACACC |  |
| so0939_Fq | CTGTGCTTCAAGGGAGGGTT | Quantitative detection of *so0939* |
| so0939_Rq | GAGCCTCAGGACTGCCATTT |  |
| so1302_Fq | ACAACAGGATCACGCAGGAG | Quantitative detection of *so1302* |
| so1302_Rq | GCGATTCAAGACGCCATCAC |  |
| so1563_Fq | GTTCTGTGCCTAACATGCCG | Quantitative detection of *so1563* |
| so1563_Rq | TCCCTGTAACCAGTTTGGCG |  |
| so1659_Fq | TAAACCACCGTCAGTTGCGA | Quantitative detection of *so1659* |
| so1659_Rq | TCCGTAATGCTGCTGGTGAG |  |
| so2052_Fq | ATCTGTGTCTTGGTGAGGCG | Quantitative detection of *so2052* |
| so2052_Rq | TTTACCGTTCCTTTGGGCGT |  |
| so2519_Fq | GCCGCCTTTGCCATATTGAG | Quantitative detection of *so2519* |
| so2519_Rq | CCCAACCCGAGAATGTCGAT |  |
| so3058_Fq | CGCCTGTTGCCATAATCACG | Quantitative detection of *so3058* |
| so3058_Rq | CGTTGAAAGTTCGGGTGCTG |  |
| so3420_Fq | ACTCCTAACTCTCGTTGCCG | Quantitative detection of *so3420* |
| so3420_Rq | GCATCGCAAAGGTTTCAGCA |  |
| sodB_Fq | AAGGAACCGATTTCGCAGGT | Quantitative detection of *sodB* |
| sodB_Rq | GCGCTACCGAAGTTGTTGAC |  |
| tsaA_Fq | GCCTTTTTCCCAACCAGCAG | Quantitative detection of *tsaA* |
| tsaA_Rq | CCCCAGTTGAAAAGGGTGGT |  |
| 16S rRNAF | GCTAATACCGCATACGCCCT | Quantitative detection of 16S rRNA gene |
| 16S rRNAR | GCCTTGGTGAGCCATTACCT |  |

Note: Underline, restriction sites. SacⅠ, GAGCTC; SphI, GCATGC; SbfI, CCTGCAGG; EcoRI, GAATTC; XbaI, TCTAGA. Italic, ribosomal binding sites.
